# MORTALITY RISK INFORMATION, SURVIVAL EXPECTATIONS AND SEXUAL BEHAVIOURS *

**DOI:** 10.1093/ej/uead116

**Published:** 2024-01-25

**Authors:** Alberto Ciancio, Adeline Delavande, Hans-Peter Kohler, Iliana V. Kohler

**Affiliations:** University of Glasgow, UK; University of Technology Sydney, Australia & Nova School of Business and Economics, Portugal; University of Pennsylvania, USA; University of Pennsylvania, USA

## Abstract

We investigate the impact of a randomised information intervention about population-level mortality on health investment and subjective health expectations. Our focus is on risky sex in a high-HIV-prevalence environment. Treated individuals are less likely to engage in risky sexual practices one year after the intervention, with, for example, an 8% increase in abstinence. We collected detailed data on individuals’ subjective expectations about their own and population survival, as well as other important health outcomes. Our findings emphasise the significance of integrating subjective expectation data in field experiments to identify the pathways that lead to behavioural change.

Individuals face substantial uncertainty about their own and others’ health status, the relationship between health inputs and outcomes, and the prevalence of common diseases. Subjective expectations about these uncertain events are therefore important determinants of health-related behaviours. Despite the centrality of health-related expectations, there is evidence that many individuals have inaccurate beliefs. These misperceptions may be a significant driver of the underinvestment in health observed in low-income countries (LICs; Dupas and Miguel, 2017; Kremer *et al.*, 2019), suggesting a benefit of better knowledge. Pessimism about survival risk is particularly notable and widespread (Delavande and Kohler, 2009; Delavande *et al.*, 2017; Shrestha, 2020). This pessimism suggests a provocative question for health policy: could policies achieve longer, healthier and ‘better’ lives by simply correcting misperceptions about survival? In other words, is there a health benefit of accurately knowing mortality risks? In this paper, we investigate the impact of a randomised information intervention about population-level mortality on individuals’ subjective health expectations and health investment. The investment of interest is the adoption of safe sex practices in sub-Saharan Africa (SSA) where HIV prevalence is high.

Prior to this study, no population-based randomised controlled trials (RCTs) have directly evaluated hypotheses about the health benefits of more accurate population mortality expectations. To fill this niche, we designed a *benefits-of-knowledge health-information intervention* (‘BenKnow intervention’). A rare feature of our study is that we collected very detailed information on individuals’ subjective expectations about their own and population survival, as well as about other important health outcomes. This allows us to analyse which subjective expectations respond to the information intervention, and in turn lead to behavioural change. Understanding the mechanisms through which the intervention influences decision-making is critical for assessing the scope of potential scale-up of interventions or application to other contexts.

Accurate knowledge about population mortality risk might promote safe sex practices in a high-HIV-prevalence environment through two possible mechanisms. First, less pessimistic population survival expectations may lead to less pessimistic expectations about own survival. Theory predicts that overall improvements in life expectancies encourage human capital investments, such as safe sex practices, as individuals can reap the returns for a longer period (e.g., Ben-Porath, 1967; Becker, 1993). Indeed, several studies have documented that actual gains in life expectancy translate into more investments in schooling and health (Jayachandran and Lleras-Muney, 2009; Oster *et al.*, 2013). We call this the *own survival* mechanism.^1^ Second, more accurate knowledge about population mortality may lead individuals to realise that HIV+ individuals live longer, making the pool of available partners riskier. Such an increase in HIV prevalence has been documented in SSA among older adults, which is the focus of this study (Vollmer *et al.*, 2017), including in Malawi (see Section 2.2). This increase in prevalence in turn increases the HIV transmission risk associated with having multiple partners, augmenting the cost of risky sex, and hence encouraging safe sex practices (e.g., Dupas, 2011a, Delavande and Kohler, 2016). We call this the *transmission risk* mechanism.

Our BenKnow intervention consists of two components. First, respondents watched three videos delivering the narrative that people nowadays live longer in Malawi with an explanation for these gains (e.g., better access to health care, availability of antiretroviral treatment (ART), fewer food shortages). Second, they received visual statistical information about the survival chances of individuals of the same age and gender. The intervention and baseline data collection was implemented in June–August 2017, and follow-up data were collected in June–July 2018.

The intervention targets mature adults aged 45 and older in rural Malawi, a study population that is particularly relevant for the BenKnow health-information intervention. First, mature adults in HIV-affected SSA countries such as Malawi have survived through periods with significant mortality fluctuations during adult ages, making it difficult for individuals to make inference about mortality risks. Indeed, mature adults aged 45+ years in the Malawi Longitudinal Study of Families and Health (MLSFH) report average subjective five-year survival probabilities of 46%–58% in the years from 2006 to 2018, compared to 83%–87% suggested by current life tables.^2^ Second, mature adults contribute importantly to the spread of HIV because they continue to be sexually active and often have younger (extramarital) partners and/or risky sexual behaviours (Dupas, 2011a). Third, the number of mature adults in Africa is projected to more than triple between 2015 and 2050 (UN Population Division, 2016), and it is critical to develop health and social policies targeted at enhancing the health and well-being of this growing subpopulation.

Our key policy-relevant finding is that the BenKnow health-information intervention resulted in a statistically significant reduction in sexual risk-taking. The magnitude of the treatment effect is substantively important. For example, one year after the intervention, the predicted probability of having multiple partners without condom use is 7.6% in the control group and 6.4% in the treatment group, corresponding to a 19% reduction in the riskiest behaviour in terms of HIV transmission. Similarly, the predicted probability of abstinence in the last 12 months is 33.3% in the control group and 36.1% in the treatment group, i.e., an 8% increase in the safest behaviour. The results are robust to alternative specifications allowing for misreporting of sexual behaviour. We also document a reduction in pregnancies and births, an outcome not affected by misreporting of sexual behaviours, in the treatment villages subsequent to the BenKnow intervention. Individuals in the treatment villages are also more likely to be married at follow-up. Marriage may be seen as a risk-reduction strategy for singles who want to commit to a low-risk partner.

We take advantage of our rich expectation data to understand why our intervention was effective at changing behaviour. We start by looking at expectations about population survival that was the primary target of the information. Our analyses document a positive treatment effect on expectations about population survival one year after the intervention: there is a 6.1% increase in the subjective probability that a hypothetical healthy individual will survive in five years, given a baseline survival expectation of 70%. The magnitude of the effect is slightly larger when looking at the survival expectations for hypothetical individuals who are HIV+ (6.6%), and individuals who are sick with AIDS, but are on ART (7.1%). These findings are important because they indicate that individuals were able to understand, process and memorise the information we provided during the health-information intervention. Interestingly, the BenKnow treatment effects are not systematically different according to baseline beliefs’ accuracy, which suggests that the overall narrative of the BenKnow intervention about changing survival patterns in Malawi had more impact on individual’s revision of expectations than the statistical information.

The positive treatment effects on population survival expectations had ramifications for other health expectations. In particular, and consistent with the *transmission risk* mechanism discussed earlier, we find a positive effect of the BenKnow intervention on the subjective probability of contracting HIV conditional on having multiple sex partners. Importantly, there is no corresponding treatment effect on the subjective beliefs about the ‘technology’ of HIV transmission, that is, infection risk conditional on behaviours and partner HIV status. Hence, the increase in the subjective transmission risk associated with multiple partners appears driven by an increase in the perceived HIV prevalence of potential partners. The positive treatment effect on the survival probabilities of HIV+ people led individuals to realise that HIV+ people remain for longer in partners’ pools.

In addition, and contrary to the *own survival* mechanism discussed earlier, the BenKnow intervention did not appear to have changed *own* survival expectations, neither in the short run (two weeks after the intervention) nor in the long run (one year after the intervention). The updating of population-level survival expectations with limited updating of own survival expectations is plausibly driven by individuals having more private information about their own survival (i.e., health, behaviour) as well as holding traditional beliefs. These render expectations about own survival much less responsive to new information. This information asymmetry is important to take into account as interventions may try to scale up programs to inform individuals about mortality and disease risks.

Our paper contributes to a growing literature on the role of information provision on health behaviour in low-income countries (for reviews, see Dupas, 2011b; Dupas and Miguel, 2017). This literature is motivated by the fact that beliefs and misconceptions may be important determinants of health behaviour (Banerjee and Duflo, 2011; Kremer *et al.*, 2019). Information interventions have been found to have large effects in some settings, and small or no effects in others, but we still have limited understanding for these overall mixed findings (Hornik, 2002; Dupas and Miguel, 2017). Typically, we do not know whether an information treatment was effective in changing beliefs in the intended direction, whether it changed other beliefs than those targeted or whether it made certain behaviours salient. The main reason is that beliefs are rarely measured in studies investigating the role of information on health behaviours, which prevents researchers from testing directly whether information was effective at changing beliefs (Kremer *et al.*, 2019).^3^ In contrast, a distinctive aspect of our study is that it provides evidence on the full chain of impacts from information to various health expectations, and in turn, health behaviours.

Our findings reveal important lessons for how to conceptualise and operationalise information interventions in general, which is important as those are increasingly rolled out (Haaland *et al.*, 2023). First, our findings indicate that not all expectations are equally malleable and that the elasticity of expectations to information may depend on the extent of private information and other characteristics, such as religious beliefs. Second, information intervention may influence a wide range of beliefs beyond those specifically targeted. For example, the BenKnow treatment effects on sexual behaviours appear primarily driven by the upward revisions of the HIV transmission risk associated with risky sex, which is an aspect that the BenKnow intervention did not mention nor target to modify. This underscores the usefulness of collecting comprehensive expectation data that could be potentially affected by information to better understand why programs fail or succeed. It also highlights that the mechanisms underlying behavioural changes might be quite complex and should be carefully modelled, with information and feedback effects from behaviour possibly impacting a wide range of expectations.^4^ These insights are crucial for other researchers who want to enhance the effectiveness of information intervention by targeting specific expectations.

From a substantive point of view, our analyses suggest that expectations of population mortality risks are a possibly important and modifiable determinant of health behaviours. Pessimistic expectations are likely to occur in populations with rapid improvements in mortality. While possibly not as dramatic as in countries affected by HIV/AIDS, social, political and economic crises as well as other health crises such as the COVID-19 pandemic have also resulted in substantial increases in adult mortality rates, and subsequent rapid recoveries of life expectancy (Brainerd and Cutler, 2005; Ruhm, 2016; Schöley *et al.*, 2022). In contemporary high-HIV-prevalence contexts such as Malawi, a BenKnow health-information intervention that reduces misperceptions about mortality risks is a potentially useful policy tool to curtail HIV infection. As such, it complements recent information interventions focusing on reducing risky sex that have shown that information on the relative risk of HIV infection by partner’s age leads to decreases in unprotected sex and pregnancies among teenagers (Dupas, 2011a), information about the HIV transmission risk leads to a reduction in sexual risk-taking for fatalistic individuals (Kerwin, 2018) and information about the reductions in HIV risk resulting from male circumcision influences circumcision uptake and sexual behaviour (Chinkhumba *et al.*, 2014; Godlonton *et al.*, 2016).

This paper also belongs to the growing literature using subjective expectation data to better understand decision-making under uncertainty without the need to rely on assumptions about expectations (Manski, 2004), including in LICs (Delavande, 2014; 2023). This line of work has focused on a wide range of decisions, including education, contraception, crime, migration, occupation, voting and retirement (Hurd *et al.*, 2004; Lochner, 2007; Delavande, 2008b; Van der Klaauw and Wolpin, 2008; Delavande and Manski, 2012; Van der Klaauw, 2012; McKenzie *et al.*, 2013; Giustinelli, 2016; Delavande and Zafar, 2019). Of particular relevance is Delavande and Kohler (2016) whose results were the primary motivation for the present study. They estimated a structural model of risky sex in Malawi, emphasising the causal effect of expectations about HIV transmission and mortality. Based on simulations, they recommended providing information on mortality risk to reduce risky sex.

Within this literature, our study complements existing work focusing on how subjective expectations are updated in response to randomly provided information within surveys. This research is often conducted with surveys that elicit priors and posteriors about outcomes such as fertility, future earnings, inflation or housing (Delavande, 2008a; Wiswall and Zafar, 2015; Armantier *et al.*, 2016; Armona *et al.*, 2018).^5^ The advantage of our design is that we can investigate the impact of the information on a large set of expectations, while other studies typically focus solely on the targeted expectations, disregarding the possibility that information may have ramifications for a wider range of beliefs. Moreover, we observe the revised expectations one year after the provision of information—a time lag substantially larger than other studies—and link the change in expectations to real-life behaviour, as opposed to stated behaviour or behaviour in incentivised lab-style tasks. Our results call for encouragement and caution: individuals in low-income settings use the information we provided to make important life-cycle decisions, but not all expectations are equally malleable and thus modifiable by interventions.

## 1. Background

### 1.1. Context and Motivation

Malawi’s Human Development Index rank of 172 out of 189 countries and territories in 2018, and its per-capita GDP is equal to about 2% of the global average. In rural areas, where our study is based and most Malawians (85%) live, the majority of individuals engage in home production of crops, complemented by some market activities. Life expectancy at birth was 59.6 for men and 66.9 for women in 2017 (GBD Collaborators, 2018). HIV prevalence among 14–49 year olds is estimated at 10.4% (women, 12.2%; men, 8.3%) in 2018, with an incidence of 4.4 per 1,000 (Malawi DHS, 2017).^6^ Despite successes in reducing HIV incidence, the HIV epidemic had, and continues to have, major effects on virtually all aspects of life, many of which were documented by the MLSFH (Kohler *et al.*, 2015). Importantly, access to ART in Malawi expanded during the past decade, attaining a 79% coverage among adults in 2018, resulting in significant reductions in adult mortality.^7^

**Fig. 1.**
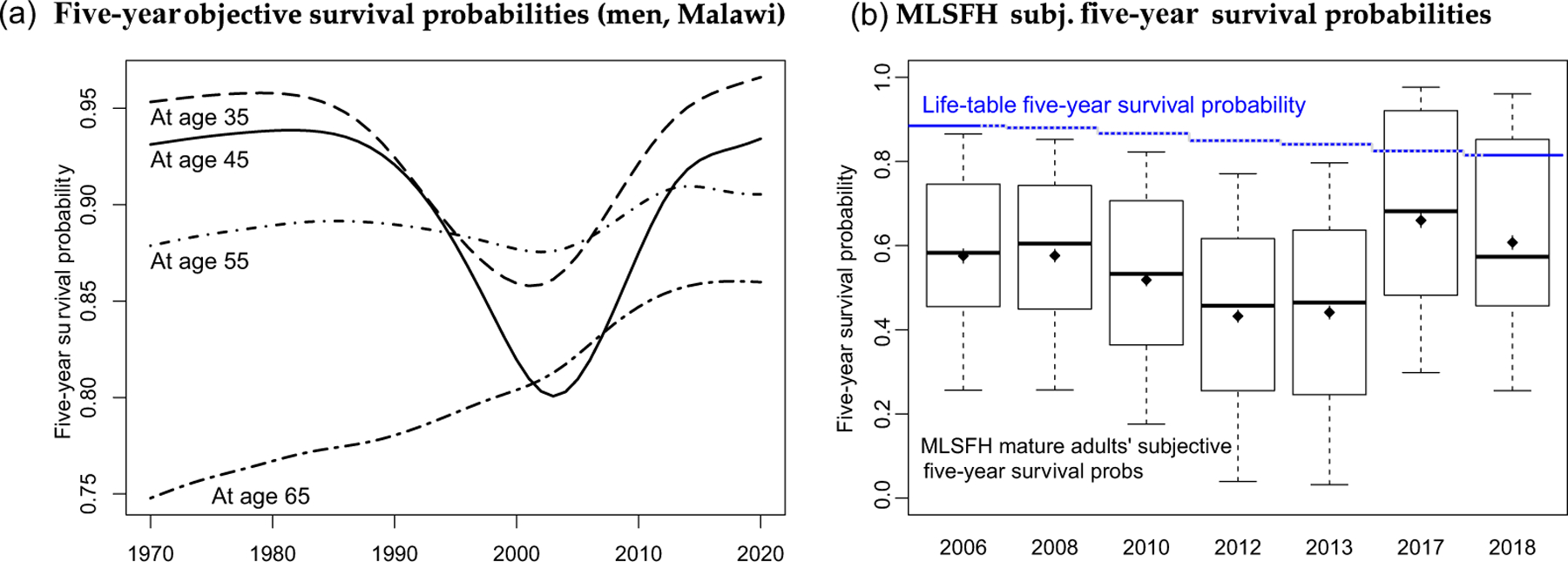
Five-Year Survival Probabilities 1970–2020 (Malawi), and Five-Year Subjective Survival Probabilities for MLSFH Mature Adults. *Notes:* Panel (a) is based on 2017 UN Word Population Prospects (UN Population Division, 2016). Panel (b) is for MLSFH mature adults (aged 45+) who participated in the 2012/13 and 2017/18 MLSFH mature adult data collections. The boxplot-like graph displays the mean (dot) and median (centre line) of the corresponding five-year survival expectations, as well as the 10th (lower whisker), 25th (bottom of box), 75th (top of box) and 90th (upper whisker) percentiles of the distribution. Life-table survival probabilities are merged by age and gender from the UN Malawi 2005–15 life tables (UN Population Division, 2016).

While reductions in multiple diseases have contributed to declining infant mortality and increasing adult life expectancy (GBD Collaborators, 2018), it is the widespread rollout of ART that is widely credited with reversing the decline of adult survival rates during the last decade (Bor *et al.*, 2013). During the HIV/AIDS epidemic, objective survival probabilities for adults changed immensely (Figure 1(a)): 35-year-old males attained a five-year survival probability of 95% around 1985, which dropped below 86% in 2002, having recovered to 96% by 2017.

In contrast to the recent trends that have given rise to a cautiously optimistic outlook about curtailing the consequences of the HIV/AIDS epidemic (UNAIDS, 2015), there is consistent evidence that mature adults in Malawi have distorted and overly pessimistic survival expectations: they substantially *underestimate* their own survival probabilities (Figure 1(b)). Until about 2013, while adult survival was improving significantly, MLSFH mature adults became increasingly pessimistic about own survival (Figure 1(b)), much more than is justified due to the respondents’ ageing. This trend was partially reversed in 2017, possibly as a result of favourable rains and an exceptionally good harvest, reverting again to more pessimistic assessments by 2018. Despite these year-to-year fluctuations and the significant variation across individuals (Figure 1(b)), the basic implication has remained unchanged: the vast majority of our mature adult study participants underestimate population survival, with pessimism being least pronounced, but still substantial (70%) in 2017 when our baseline was implemented.

The frequent experience of poverty-related and HIV/AIDS-related mortality and socioeconomic shocks during the last two decades and the overestimates of salient health risks such as HIV prevalence and HIV transmission probabilities are likely the driving factor behind the elevated mortality expectations in our study population, and the resulting pessimism about own and population-level survival rates (Figure 1).^8^ Also potentially contributing to mortality misperceptions are common cognitive biases such as denominator neglect or salience biases, often documented among health-care professionals, where individuals fail to accurately relate events (such as deaths) to exposures (denominator counts or person years lived; Tversky and Kahneman, 1973).

### 1.2. Mature Adults, Sexual Risk-Taking and the HIV-AIDS Epidemic

Our study focuses on mature adults aged 45 and older for several reasons. First, and most important for this study, mature adults continue to be sexually active (Online Appendix Figure C.1), engage in risky sexual behaviours (Freeman and Anglewicz, 2012) and importantly contribute to the spread of HIV across all age groups (Vollmer *et al.*, 2017). A substantial fraction of older men (‘sugar daddies’) engages in sex with younger women (Dupas, 2011a). Two-thirds of the MLSFH mature adults had sex in the last 12 months (51% for women and 85% for men), and 57% of respondents had sex exclusively with their spouse during the last 12 months (50% for women and 67% for men). Marriage and divorce/widowhood among mature adults are common, and remarriage is often swift (Reniers, 2003). Mature adults are also less likely to adopt safe sexual behaviours, discuss HIV prevention with partners or disclose an HIV-positive status within relationships compared to younger persons (Freeman and Anglewicz, 2012).

Second, and related to the transmission risk mechanism discussed in the introduction, the HIV prevalence is increasing among mature adults. In 2017, 8% of MLSFH mature adults tested HIV positive, corresponding to an increase of 40% since 2012 that is driven by respondents aged 45–49 years. Similar patterns occur more broadly across SSA due to increased survival of HIV+ persons in cohorts that obtained access to widespread ART in middle adulthood. For example, using data from 27 SSA countries between 2003 and 2012, Vollmer *et al.* (2017) found an average annual growth rate of HIV prevalence of 4.2% for older adults, while HIV prevalence is decreasing at a rate of 3.5% for adults aged below 45 years. Data from the Malawi DHS show that HIV prevalence is increasing for adults 45 and above (Online Appendix Figure C.2). These dynamics are changing the ‘hump-shaped’ age pattern of HIV prevalence with peak HIV prevalence for ‘young’ mature adults around age 45–50. The HIV risk associated with risky sexual behaviours of mature adults is further exacerbated by the fact that HIV+ mature adults are disadvantaged in terms of accessing ART. Most HIV testing campaigns, which provide a primary gateway to treatment for HIV+ individuals, focus on primary reproductive ages, and in rural Malawi, routine HIV testing of women and their partners is primarily conducted as part of pre-natal care.^9^ Importantly for our subsequent interpretation of our results, the imperfect ART uptake and adherence implies that HIV+ mature adults are often not virally suppressed. They remain sources of HIV infection to their partners, and as the HIV prevalence among mature adults increases due to increased survival of HIV+ adults, the sexual partners of mature adults face increased HIV risk.

Third, because ART was not available throughout much of their adult lives, MLSFH mature adults have a heightened awareness about the importance of sexual and marital behaviours as a critical aspect of investing in health across the life course. While the relationships between behaviours and health evolved as individuals got older, epidemiological contexts changed (e.g., increased relevance of non-communicable diseases) and new technologies became available (e.g., ART) for MLSFH mature adults, the triad between sexual/marital behaviours, health and survival continues to be closely intertwined. In perceptions as well as in reality, changes in sexual risk-taking continue to be primary mechanisms of reducing HIV infection risks and ensuring long-term health among mature adults.

Fourth, the (actual) mortality risks in contexts such as Malawi continue to be relatively high at mature adult ages (Figure 1). As a result, survival expectations are arguably important for life-course decision-making and well-being. Finally, mature adults are an essential subpopulation in SSA LICs because of their growing demographic relevance, their almost universal labour force participation with virtually no retirement, their important contributions to intergenerational transfers and their pivotal caretaking roles in families affected by HIV/AIDS (UN Population Division, 2016).

## 2. Data and BenKnow Health-Information Intervention

### 2.1. Mature Adult Cohort of the MLSFH

The MLSFH is an ongoing longitudinal panel study established in 1998 that examines how families and individuals cope with the social, economic, demographic and health consequences of the HIV/AIDS epidemic (Kohler *et al.*, 2015). Our BenKnow study is based on the MLSFH mature adult cohort (MLSFH-MAC), which was established by selecting in 2012 MLSFH respondents aged 45+ years, and enrolling them as part of an extensive ageing and health baseline survey with follow-up waves in 2013, 2017 and 2018 (Kohler *et al.*, 2020).^10^ In 2017 and 2018, the two waves that are primarily relevant for the BenKnow study, the MLSFH-MAC collected a broad range of information, including detailed data on probabilistic expectations and sexual behaviours. Prior to the 2017 waves, we also conducted qualitative interviews with about 35 respondents to get feedback on the information intervention and insights into respondents’ revision processes.

**Fig. 2.**
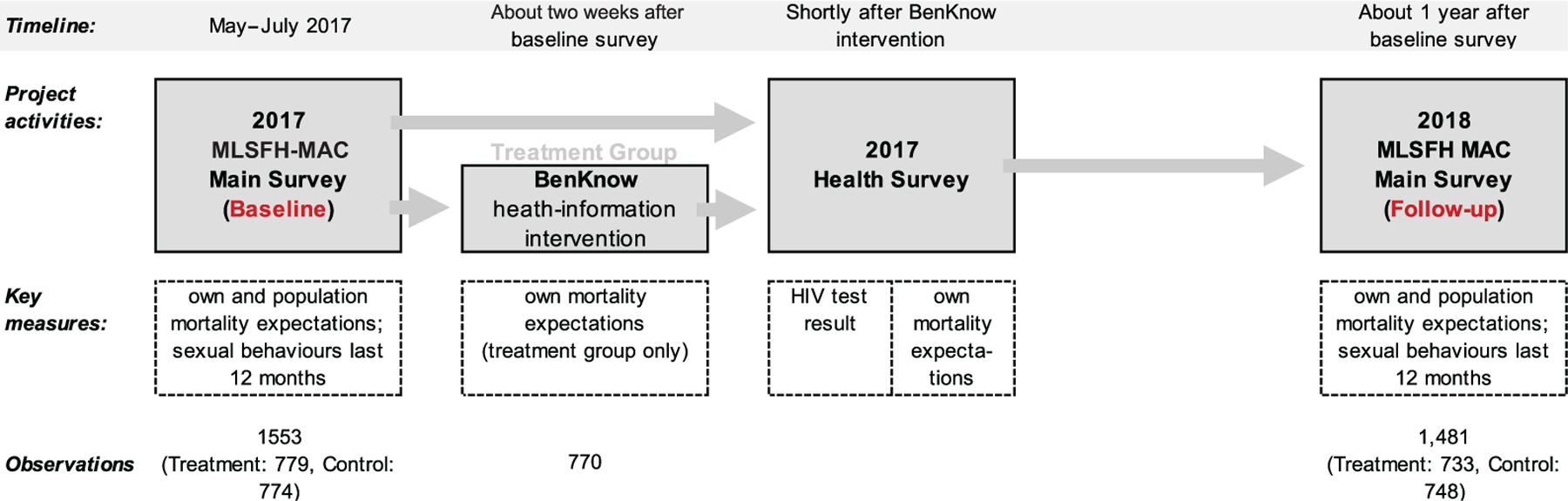
Research Design and Sequence of Study Activities.

The BenKnow health-information intervention was implemented by a separate team within two weeks subsequent to the 2017 MLSFH-MAC main survey. Shortly after the BenKnow intervention, an HIV testing and counselling (HTC) team visited the respondents in both the treatment and control groups to administer HIV testing and counselling sessions followed by a short survey.^11^ Take-up of the HIV test was essentially universal (97.4%), and virtually all respondents opted to receive the result of the HIV test.^12^ The 2018 MLSFH-MAC study population, fielded about one year after the 2017 wave, constitutes our *follow-up* survey. Our final analysis sample includes 1,481 respondents who completed all the required surveys (the 2017 and 2018 surveys and the intervention if in the treatment group). Attrition from 2017 to 2018 was less than 5%, and attrition rates are similar by treatment status.^13^ Figure 2 presents the timeline of data collection.

### 2.2. BenKnow Health-Information Intervention

The BenKnow intervention randomly assigned 2017 MLSFH-MAC respondents to a treatment and a control group, with randomisation occurring at the village level to avoid spill-over effects between groups. Within each of the three study regions, villages were paired by size starting from the two biggest villages, followed by the two second biggest, etc. Then we randomly assigned treatment status to one village in each pair. The procedure guaranteed a similar sample size in the treatment group ( *N =* 779 in 58 villages) and control group ( *N =* 774 in 57 villages). The response rate for the BenKnow intervention was more than 98% (among 2017 survey respondents), resulting in 770 respondents enrolled in the treatment group. The BenKnow intervention was implemented individually. It consisted of the following two core components, with the complete interviewer scripts and additional information provided in the Online Appendix.

- *Narratives about changing mortality provided by video clips.* Respondents were initially shown three video clips with a duration of about four minutes each. In these short video clips, individuals (trained local actors following a prepared script) explained how they noticed that people nowadays live longer in rural Malawi. The first video depicts a carpenter in his workshop, the second a female tailor in her shop sitting at a sewing machine and the third an old man sitting in front of his house. The videos emphasise overall that people live longer due to better access to food, health care and availability of ART. Studies support that video narratives are a useful way to convey scientific information to non-experts by increasing comprehension, interest and engagement (Bruner, 2009). Evidence presented via such narratives is also more likely to be memorised (Schank and Berman, 2002). Respondents related strongly to these videos. For example, in our qualitative interviews, they reported that ‘the videos tell the truth’ or that the ‘man reminds me of my late husband’ or that it made them think about ‘their brother and sister who both died from HIV’.
- *Life-table survival probabilities conveyed via visual aids.* Subsequent to the videos, respondents were shown a health-information sheet with visual information on five-year and ten-year life-table survival probabilities for individuals of the same gender and within the same five-year age group, with different figures conveying how many persons, out of ten alive at the time of the intervention, could be expected to be alive five or ten years in the future.^14^ A BenKnow health-information sheet is illustrated in Online Appendix Figure B.1, and Online Appendix Table B.1 reports the complete set of BenKnow age- and gender-specific five- and ten-year survival and death probabilities. The statistics purposely emphasised both the survival and mortality risk to avoid anchoring. While the videos conveyed a general narrative of improved survival, the life-table probabilities provided precise statistical information about mortality risk.

Data collected during the treatment reveal that 98% of the respondents reported understanding the information provided, and 79% state that the BenKnow information reflects correctly what happens in their community, with 15% stating that it reflects somewhat correctly.

### 2.3. Balance at Baseline

Column (1) in Table 1 reports summary statistics for the MLSFH-MAC cohort in 2017, the *baseline* for our BenKnow study. Respondents are 59 years old on average, 60% are female,^15^ they only have on average 3.5 years of schooling and 7.5% tested positive for HIV. Virtually all respondents have been married at least once in their lives, but separations and remarriages are frequent. At baseline, 73% are married, 18% are widowed and 9% are divorced or separated. Columns (2) and (3) of Table 1 confirm that the treatment and control groups are comparable on baseline observable characteristics. Importantly, own and population survival probabilities (which we describe in detail in the next section) are very similar, and the sample is well balanced according to age, gender, sexual behaviour, marital status and years of schooling. HIV prevalence is higher in the treatment group (8.7% versus 6.3%, statistically significant at the 10% level), and, as a result, we observe a slight imbalance in the subjective probability of being infected with HIV and also in the survival probability conditional on being HIV+. When we restrict our analysis to individuals who tested negative for HIV in 2017, all variables are balanced at conventional statistical levels, including beliefs about HIV status and survival probabilities conditional on being HIV+ (Columns (5) and (6) of Table 1). Our main analyses focus on the entire sample, but the Online Appendix presents results with interaction of HIV status and treatment, as well as for HIV− individuals only.

### 2.4. MLSFH Data on Subjective Expectations

Detailed subjective expectation data have been a hallmark of the MLSFH since 2006 (Delavande and Kohler, 2009; 2012; 2016), including expectations about mortality (own and population), HIV infection and transmission, and the experience of socioeconomic shocks. These expectations were elicited by asking respondents to allocate up to ten peanuts (prior to 2017, beans) on a plate to express the likelihood that an event will occur, allowing respondents to split a peanut in half when stating their expectations.^16^ The following MLSFH expectations are of particular relevance for the present study, with Online Appendix A providing the full text of the 2018 MLSFH expectation module and Figure 2 showing when these various expectations were collected.

- *Own mortality expectations,* reflecting respondents’ subjective expectations that they would die within a five-year and ten-year time horizon from the day of the interview (‘*Pick the number of peanuts to express the likelihood that you will die with a 5-year [10-year] period beginning today.*’). Own mortality expectations were elicited up to four times: during the 2017 MLSFH-MAC survey, during the BenKnow intervention (treatment group only), during the HTC survey shortly after the intervention (treatment and control groups) and about one year after the BenKnow intervention during the 2018 MLSFH-MAC survey.
- *Population mortality expectations,* measuring respondents’ perceived likelihood that the following hypothetical individuals of a specified health status would die within a five-year period: (*i*) a woman/man who is healthy and does not have HIV; (*ii*) a woman/man who is infected with HIV; (*iii*) a woman/man who is sick with AIDS; (*iv*) a woman/man who is sick with AIDS and is treated with ART. All hypothetical individuals were described as being of the same age and gender and living in the same context as the respondent (‘*Pick the number of peanuts that reflects how likely you think it is that one of the following persons will die within a five-year period beginning today: A man [woman] your age who is healthy and does not have HIV?*’, and variations thereof for (*ii*)–(*iv*)). We also ask about the five-year mortality expectations of ‘someone like you’ (‘*A person of your sex and age in your community*’). Population mortality expectations were elicited during the 2017 MLSFH-MAC survey and during the 2018 MLSFH-MAC survey.
- *HIV-related expectations,* measuring (*i*) the subjective probability of the respondent being currently infected with HIV; (*ii*) the perceived likelihood that his/her spouse is currently infected with HIV; and in 2010 and 2018 also (*iii*) the subjective expectation of becoming infected with HIV within the next 12 months conditional on various sexual behaviour, including if married to someone who is infected with HIV/AIDS and if one has several sexual partners in addition to the spouse (‘*Pick the number of peanuts that reflects how likely you think it is that you are infected with HIV/AIDS now*’, and variations thereof for (*ii*)–(*iii*)).

The above mortality expectations are converted to survival probabilities (= 1 minus #peanuts divided by 10) and respectively referred to as *own survival* probabilities and *population survival* probabilities. Survival probabilities are generally consistent with each other in terms of time horizon and health status (column (1) of Table 1). Respondents reported in 2017 on average a 67% chance of surviving for the next five years, and a 44% chance of surviving for the next ten years. They expect a hypothetical healthy individual to have a 70% chance of surviving for the next five years, compared to 62% for someone who is HIV+, 49% for someone who is sick with AIDS and 57% for someone who is treated with ART. The chance of surviving not conditional on health status is 69%, which is just below the average reported survival for healthy individuals. There is substantial variation in survival probabilities (Figure 1), with answers taking all values between 0 and 1, some heaping at 0.5 and 1, and few respondents taking advantage of the possibility to split the peanut (less than 3%) to indicate probabilities at 5-percentage-point intervals (Online Appendix Figure C.3). Importantly, there is substantive information conveyed in these probabilistic expectations: respondents who reported a lower probability of surviving to the next five or ten years in 2010 are less likely to be alive in 2017 (Online Appendix Figure C.4).

### 2.5. MLSFH Data on Sexual Behaviour

Sexual behaviour in the MLSFH MAC is captured via questions about whether the respondent had sex in the last 12 months, the number of sexual partners in the last 12 months and whether a condom was used in the last sexual intercourse. Based on these questions, we construct three indicators of risky sexual behaviour to look at both the extensive (being sexually active) and the intensive margins (multiple partners and condom use). Summary statistics for the variables entering the sexual risk indices are reported in Table 1.

- *Sexual risk index 1 (SRI1):* 0 = not sexually active in the last 12 months, 1 = sexually active in the last 12 months.
- *Sexual risk index 2 (SRI2):* 0 = not sexually active in the last 12 months, 1 = sex with one partner, 2 sex with multiple partners.
- *Sexual risk index 3 (SRI3):* 0 = not sexually active in the last 12 months, 1 = sex with one partner, 2 = sex with multiple partners and a condom at last intercourse, 3 = sex with multiple partners and no condom at last intercourse.

Self-reported sexual behaviour questions have been consistently shown to correlate with biomarker-based or pregnancy-based indicators of sexual behaviour (McClelland *et al.*, 2011).^17^ Yet, we recognise the fact that self-reported sexual behaviour is often difficult to measure through self-reports, and the above variables may be subject to measurement error. We discuss the robustness of our results to potential misreporting in Section 4.1, using analytic approaches that allow for measurement error in the reporting of sexual behaviours as well as newly collected 2019 MLSFH data on pregnancy outcomes in the BenKnow treatment and control villages.

## 3. Conceptual Framework

**Fig. 3.**
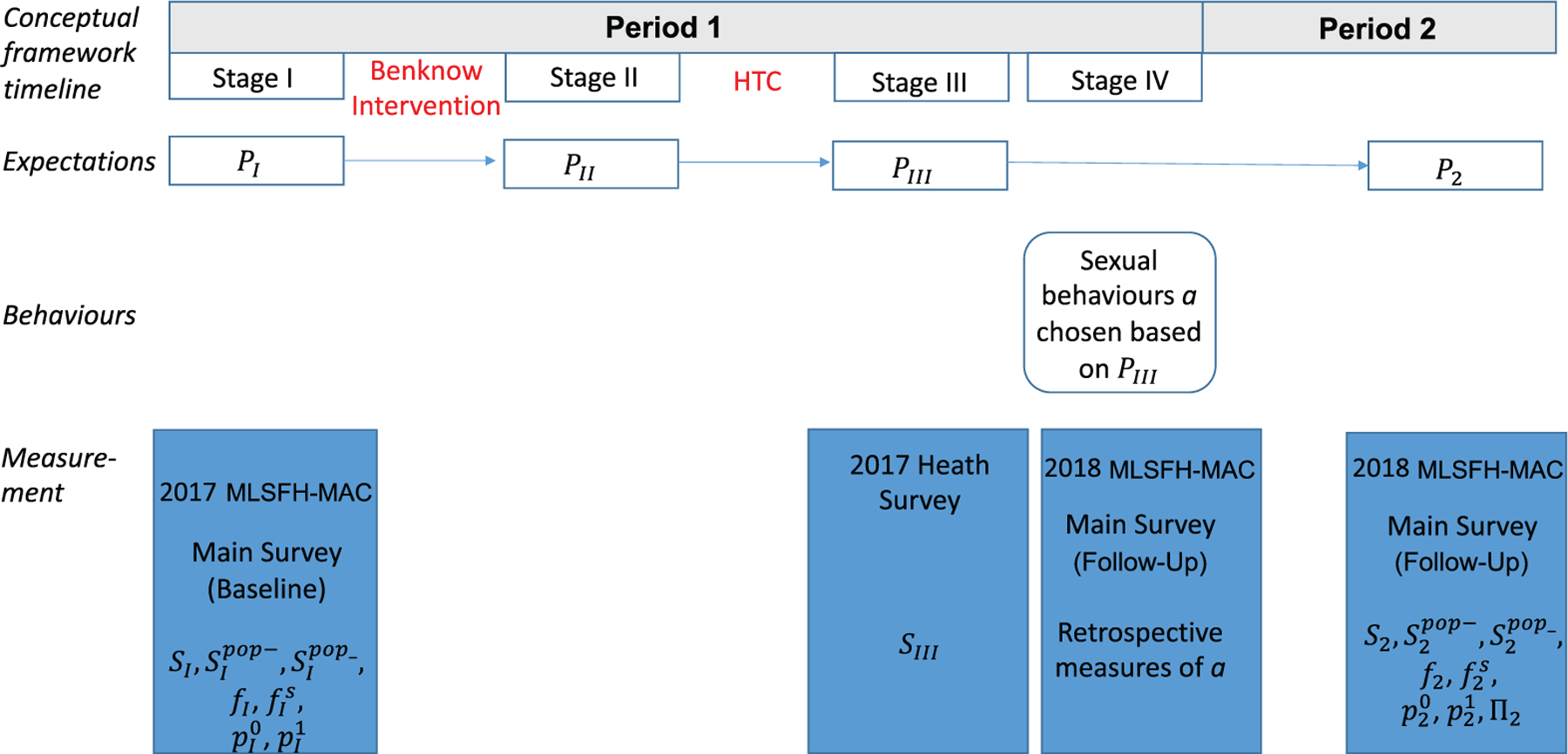
Model Timeline.

We present a simple conceptual framework that highlights the interrelations between subjective expectations and sexual behaviours within a life-cycle framework similar to that from Delavande and Kohler (2016). The periods and stages closely mirror the various data collection steps and are presented in Figure 3.

### 3.1. Sexual Behaviours, Subjective Expectations and Mortality Information

#### 3.1.1. Decision-making process

Consider an individual living for two periods. In period 1, the individual is endowed with a set of individual-specific subjective expectations P. The set P encompasses the following three aspects: (*1*) survival to the next period, (*2*) HIV status and (*3*) HIV transmission risks. At the end of period 1, she engages in sexual behaviour a. For tractability, we consider two levels of sexual behaviour: safe sex (such as sex with a spouse only), denoted by a=0, and risky sex (such as sex with extra-marital partners in addition to a spouse), denoted by a=1. The individual enjoys an immediate utility from sex $V\left(a\right)$ in period 1. In period 2, she makes no further decision and, if alive, enjoys a health-dependent utility equal to $U^{-}>0$ if HIV− and $U^{+}=U^{-}-c$, with $0<c<U^{-}$, if HIV+. The subjective expected lifetime utility if she chooses a is given by

(1) $$V(a)+fS^{+}U^{+}+(1-f)\left[\left(1-p^{a}\right)\left(S^{-}U^{-}\right)+p^{a}\left(S^{+}U^{+}\right)\right].$$

The period 2 utility is discounted by the subjective probability of surviving to period 2.^18^ With perceived probability f, the individual believes that she is HIV+ at the beginning of period 1, with the survival probability $\left(S^{+}\right)$ of an HIV+ person. With probability (1−f), she believes that she is HIV− and faces the risk of becoming infected at the end of period 1 with the transmission probability $p^{a}$, and a survival probability $\left(S^{+}\right)$. She can remain HIV− with probability $\left(1-p^{a}\right)$ and faces the survival probability $\left(S^{-}\right)$ of an HIV− person. Within this framework, engaging in risky sexual behaviour may increase the immediate pleasure derived from sex in period 1. However, it can also potentially increase the subjective risk of becoming HIV+, which in turn may decrease the subjective probability of surviving into the future and enjoying future period utility, while also decreasing the probability of enjoying $U^{-}$ rather than $U^{+}$.

Equation (1) is identical to the framework developed by Delavande and Kohler (2016). The main difference pertains to the beliefs that are used in this equation that are potentially revised in this context before making a decision. Before period 1, the individual is randomly allocated into a treatment or a control group. Period 1 is divided into four stages that closely match the data collection timeline (Figure 3). The individual starts at baseline (*stage I in period 1*) with some prior expectations $P_{\text{I}}$. In stages II and III, she may revise her expectations. In particular, at the BenKnow intervention stage (*stage II in period 1*), individuals in the treatment group T receive information about population mortality risk. This information may lead them to revise any of their baseline beliefs $P_{\text{I}}$ to $P_{\text{II}}^{T}$. At the HTC stage (*stage III*), the individual learns her HIV status and may revise her expectations. So the individuals’ stage III subjective expectations differ by both the HIV test result and BenKnow treatment assignment.

Subsequent to HTC, the individual chooses her sexual behaviour (*stage IV in period 1*). Subjective expected lifetime utility at the end of stage III depends on stage III subjective expectations and the sexual behaviour a. It is given by (1) using the expectations $f_{\text{III}}$, $S_{\text{III}}^{+}$, $S_{\text{III}}^{-}$ and $p_{\text{III}}^{a}$. The individual will choose risky sex a=1 if and only if the subjective expected lifetime utility associated with risky sex is greater than that associated with safe sex, i.e.


(2) $$V(1)-V(0)>\left(1-f_{\text{III}}\right)\left(p_{\text{III}}^{1}-p_{\text{III}}^{0}\right)\left(\left(S_{\text{III}}^{-}-S_{\text{III}}^{+}\right)U^{-}+S_{\text{III}}^{+}c\right).$$

We maintain the assumptions that (*a*) the perceived HIV transmission risk associated with safe sex is smaller than that associated with risky sex (i.e., $p_{\text{III}}^{1}-p_{\text{III}}^{0}\geq 0$) and (*b*) the subjective survival conditional on being HIV− is larger than that conditional on being HIV+ $\left(S_{\text{III}}^{-}-S_{\text{III}}^{+}\geq 0\right)$, to ensure that the right-hand side of (2) is positive.

In *period 2*, the individual makes no further decisions, but enjoys period 2 utility and revises her beliefs to $P_{2}^{T}$ if in the treatment group and $P_{2}^{C}$ if in the control group.

#### 3.1.2. Primitives of expectations

The behaviourally relevant expectations highlighted in (2) may be a function of other health expectations. Thinking about these other expectations is important when looking at the revision process. We are overall agnostic about these relations, but highlight potential inputs (or primitives) below. The set of expectations we discuss and their notation is presented in Table 2.

Population survival expectations may be an input into own survival expectations. We may have $S^{-}=S^{-}\left(S^{pop-},\omega \right)$ and $S^{+}=S^{+}\left(S^{pop-},\omega \right)$, where ω denotes other relevant inputs. We expect a positive relationship between population survival and own survival. A positive correlation between population and self-beliefs has been documented in the context of expected earnings (e.g., Wiswall and Zafar, 2015; Delavande and Zafar, 2019). Moreover, Wiswall and Zafar (2015) found that information about population earnings leads to a revision in expectations about own earnings, although somewhat inelastically. In our baseline data, the correlation between own survival expectation and healthy population survival expectation is 0.38 (*p*-value *<* 0.01) (and 0.32, *p*-value *<* 0.01, for HIV+ population survival).

We also highlight various primitives to the transmission risk expectations. The probability $p^{0}$ of contracting HIV associated with the safe action a=0 may depend on the probability Π of contracting HIV if having regular sex with an HIV+ partner (i.e., the technology of HIV transmission when holding the partner’s HIV+ status constant), and the probability $f^{s}$ that the spouse is infected with HIV. In particular, in a Bayesian setting, $p^{0}=\Pi \times f^{s}$.

We similarly expect the probability $p^{1}$ of contracting HIV with action a=1 to be an increasing function of both the HIV transmission technology Π and the perceived local HIV prevalence $L^{HIV}$. Importantly, the local HIV prevalence may itself be a function of population survival expectations. Indeed, there has been a 40% increase in the HIV prevalence of older individuals between 2012 and 2017 among MLSFH respondents (see Section 2.2) and a large increase in HIV prevalence among DHS respondents age 45 and above (Online Appendix Figure C.2), which is consistent with the actual increase in life expectancy of older HIV+ individuals. So we expect $p^{1}=p^{1}\left(\Pi ,S^{pop-},S^{pop+},\rho \right)$, where ρ denotes other relevant inputs.

### 3.2. Hypotheses on BenKnow Treatment Effects

**Fig. 4.**
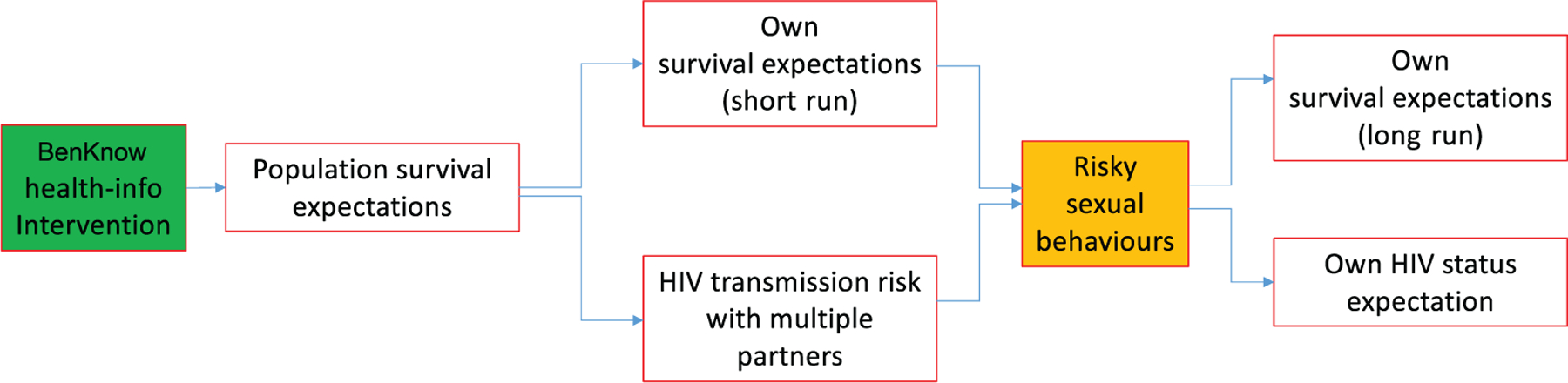
Hypotheses.

We now discuss the potential impact of the BenKnow information treatment on subjective expectations and sexual behaviour. The various hypotheses are also described in Figure 4.

#### 3.2.1. Subjective expectations

##### Class of expectations:

Note that we measure the expectations $P_{\text{I}}$ in stage I of period 1 (baseline), and $P_{2}$ in period 2 (follow-up; see Figure 3). With the exceptions of own survival expectations *S*_III_ measured by the HTC team, the other expectations $P_{\text{III}}$ are not observed. Given this timing, it is useful to consider two classes of expectations to conceptualise the effect of the information treatment.

1. Expectations about outcomes for which an individual has *no* control (population survival, local HIV prevalence, HIV transmission risk conditional on behaviour). For this class, the difference between $P_{\text{III}}^{T}$ and $P_{\text{III}}^{C}$ will be essentially the same as the difference between $P_{2}^{T}$ and $P_{2}^{C}$ and driven by the BenKnow information only.^19^
2. Expectations about outcomes for which individuals have *some* control through their behaviours (own survival expectations, the probability of being HIV+, which may be shaped by sexual behaviour). For this class, the difference between $P_{2}^{T}$ and $P_{2}^{C}$ is driven by both the BenKnow information and choices that took place after the BenKnow intervention that may differ by treatment status (e.g., risky sex). Importantly, the extra measurement $S_{\text{III}}$ enables us to identify the treatment effects on own survival expectation driven by the BenKnow information only.^20^

##### Main hypotheses on expectations:

We discuss our various hypotheses below.

*Population survival expectation*. The BenKnow treatment provided information on population survival emphasising gains for both HIV+ and HIV− individuals. Because population survival expectations are underestimated on average at baseline, we expect a positive treatment effect on population survival expectations for HIV− individuals $S_{2}^{pop-}$ and for HIV+ individuals $S_{2}^{pop+}$ (Hypothesis 1).

As described in the previous section, population survival expectations are likely to be an important input for other individual-specific health risks. The treatment effect on population survival expectations is thus likely to have a trickle-down effect for other health expectations, in particular own survival expectation and HIV transmission risk.

*Own survival expectation*. We expect a positive treatment effect on own survival expectations $S_{\text{III}}$ (Hypothesis 2a) in the short run. Such an increase would take place if individuals perceive a positive correlation between population survival and own survival. If the intervention reduced risky sexual behaviour (see the discussion below), we expect an even larger positive treatment effect on own survival expectations $S_{2}$ in the long run (Hypothesis 2b) as the change in behaviour would magnify the short-run effect.

*Transmission risk expectation*. We expect a positive treatment effect on the subjective probability of contracting HIV if one has extra-marital partners $p_{2}^{1}$ (Hypothesis 3). A positive treatment effect on the survival of HIV+ persons $S_{2}^{pop+}$ may lead respondents to realise that HIV+ individuals remain in the pool of sexual partners for longer, hence increasing the local HIV prevalence and, as a result, the transmission risk associated with having multiple partners.

*Probability of being infected with HIV*. If the intervention reduced risky sexual behaviour (see the discussion below), we also expect a negative treatment effect on the probability $f_{2}$ of being HIV+ (Hypothesis 4). Note that a lower probability of being HIV+ increases the motivation for safe sex practices (see (2)).

##### Heterogeneity by baseline perception gap:

Our information intervention had two components (Section 2.2). If respondents react to the precise life-table information provided in the intervention, we expect heterogeneity in the revision process, with more revisions for respondents who started with more inaccurate expectations. If respondents respond to the general narrative of improvement in survival, we expect a general upward revision. In our analysis, we investigate heterogeneity in revision to expectations according to the baseline perception gap (i.e., difference between subjective probability and the life-table probability provided in the intervention).

#### 3.2.2. Sexual behaviour

We anticipate overall a negative BenKnow treatment effect on the propensity to engage in risky sex (Hypothesis 5). The above BenKnow treatment effects on subjective expectations will be crucial for our ability to identify the mechanism(s) underlying changes in sexual behaviours, and we focus in particular on two potential mechanisms.

- *Own survival mechanism.* A positive treatment effect in own survival expectation $S_{\text{III}}$ (Hypothesis 2a) driven by a joint increase in $S_{\text{III}}^{-}$ and $S_{\text{III}}^{+}$ that leaves the relative mortality risk $\left(S_{\text{III}}^{-}-S_{\text{III}}^{+}\right)$ unchanged increases the right-hand side of (2), reducing the propensity for risky sex. Intuitively, a general improvement in own non-HIV and HIV survival risk increases the weight of future utility, and hence the benefits from safe sex (e.g., Becker, 1993). Although less likely because the intervention emphasised a variety of factors driving the gains in survival, it is still possible to have a joint increase in $S_{\text{III}}^{-}$ and $S_{\text{III}}^{+}$ leading to a rise in the relative survival risk $\left(S_{\text{III}}^{-}-S_{\text{III}}^{+}\right)$. This would also increase the right-hand side of (2) and promote safe sex practice. Intuitively, an increase in the relative survival risk by HIV status makes contracting HIV more costly in terms of mortality (Philipson and Posner, 1993; 1995).^21^
- *Transmission risk mechanism.* A positive treatment effect on the transmission risk associated with having multiple partners $p_{\text{III}}^{1}$ (Hypothesis 3) would increase the right-hand side of (2), reducing the propensity to engage in risky sex. Intuitively, an increase in the subjective risk of becoming HIV+ associated with the risky sex action makes it less appealing.

For illustration purposes, our model abstracts from partnership formation. It is possible that the BenKnow intervention affected marriage rates, although the effect is a priori ambiguous. For example, for single individuals, marriage may be perceived as an HIV risk-reduction strategy (Greenwood *et al.*, 2017), and the BenKnow intervention may thus encourage it. Conversely, an individual married to an unfaithful spouse may seek to adopt a safer behaviour such as abstinence, either within marriage or divorce. Other considerations may also be relevant. For example, higher survival rates of potential or current spouses increase the benefits of marriage.

## 4. Results

We initially analyse the effects of the BenKnow intervention on sexual behaviours (Section 4.1), and subsequently explore the mechanisms through which the BenKnow intervention affected sexual behaviours (Section 4.2).

### 4.1. Sexual Behaviours

The sexual risk indices 1–3 (SRI1–SRI3; see Section 2.4) are our primary categorical outcome variables for identifying BenKnow effects on sexual behaviours. Specifically, we estimate an ordered probit model for the 2018 sexual risk index $a_{ij\left(2018\right)}$ for individual i in village j as

(3) $$P\left(a_{ij(2018)}\right)=\text{Φ}\left(\beta T_{j}+\sum_{k}\delta_{k}a_{ij(2017)}^{k}+{\text{X}}_{ij}\gamma +\sum_{s=1}^{S}\tau_{s}I_{j\epsilon S}\right),$$

where, as in our previous analyses, $T_{j}$ is a dummy equal to 1 if village j is assigned to the BenKnow treatment group, the $\tau_{s}$ are strata fixed effects, $I_{j\epsilon S}$ is an indicator for whether village j is in strata s, S is the total number of strata and the $X_{ij}$ include individual baseline characteristics. The model also includes dummies $a_{ij(2017)}^{k}$ for each category k of the 2017 sexual risk index to control for baseline sexual behaviours. SEs are clustered at the village level.

We show additional results for pregnancy and marriage. For these binary outcomes, we use linear probability models in a specification similar to (3). For marriage, we control for baseline marital status, but we do not have a baseline for pregnancy.

Panel A in Table 3 reveals a key policy result: the BenKnow health-information intervention significantly reduced the propensity to engage in risky sexual behaviour across all three indices of risky sex (SRI1–SRI3), as is evidenced by a negative and precisely estimated treatment effect. To better assess the magnitude of the effect, panel B of Table 3 provides the predicted probabilities of sexual risk taking based on panel A, column (3). It shows that the intervention had a large impact on risky sex, and that it was effective at changing behaviour both at the extensive margin (being sexually active) and intensive margin (number of partners and condom use). For example, the predicted probability of having multiple partners with no condom is 7.6% in the control group and 6.4% in the treatment group, a reduction of 1.2 percentage points or 19%. Similarly, the predicted probability of not having sex is 33.3% in the control group and 36.1% in the treatment group, an increase of 3 percentage points or 8%. Focusing on HIV− respondents, we get a 12% reduction in the predicted probability of having multiple partners with no condom and a 7% increase in abstinence (Online Appendix Table C.1).

Online Appendix Table C.1 also reveals that, while men act on both margins as a response to treatment, women only adjust their extensive margin: the BenKnow intervention reduced the average predicted probability of having multiple partners by 3.4 percentage points for men and 0.4 points for women. There are no significant interactions of the treatment effect with HIV status (Online Appendix Table C.2, bearing in mind that the number of HIV+ respondents in our sample is small). Our main results hold even if we exclude polygamous men, who constitute 6.7% of our sample (Online Appendix Table C.3).^22^ We also unpack the SRI3 index into various conditional outcomes and find that most of the adjustment on the intensive margin is through the number of partners (Online Appendix Table C.4). Note that we are however under-powered to investigate condom use conditional on having multiple sex partners.

#### 4.1.1. Misreporting and pregnancies

Misreporting of sexual behaviour is a possible concern for the interpretation of our key findings on sexual risk-taking. To evaluate the robustness of our results to misreporting, we follow Hausman *et al.* (1998) to correct for misclassification error in a binary choice model. We assume that individuals report truthfully when they engage in safe sex practices and that there is a constant probability of misreporting safe sex when engaging in risky sex. Our result from this analysis shows a negative and precise BenKnow treatment effect.^23^

Our key finding about the BenKnow treatment effect on sexual behaviours is further corroborated by using an objective measure of sexual activity: pregnancies. MLSFH mature adults, i.e., the population to whom the BenKnow intervention was targeted, are generally too old to become pregnant. Instead, our robustness tests based on pregnancy outcomes focuses on younger members of the MLSFH cohort who have been interviewed in 2019 (one year after the 2018 MLSFH-MAC follow-up on which our primary results are based).^24^

The primary mechanism allowing us to identify BenKnow treatment effects on pregnancies among female MLSFH respondents younger than 45 years (and who were therefore not eligible for enrolment in the BenKnow study) is as follows: if MLSFH mature adults have sex with younger spouses or partners in their villages, as a substantial fraction in all likelihood does, then changes in sexual behaviours among mature adults in response to BenKnow can potentially reduce pregnancy risks among women *<* 45 years old. Specifically, since only two years have passed between the 2017 BenKnow intervention and the 2019 MLSFH survey, we expect a negative treatment effect on being pregnant in 2019 or having a baby less than a year old, while there should not be any treatment effect on having an infant who is one year or older.

Our results from a linear probability model in Table 4 confirm this hypothesis and provide additional support for the robustness of key results on sexual risk-taking. Specifically, column (1) shows the treatment effect on being currently pregnant or having a baby less than a year old for women in the 2019 survey and displays a precisely estimated negative treatment effect of 3.7 percentage points. Reassuringly, we do not find any significant treatment effects for infants 1–2 years or 3–4 years old (columns (2) and (3)), which also confirm balance in fertility across treatment and control villages prior to the intervention.

#### 4.1.2. Marriage

As discussed in Section 3.1, it is possible that the BenKnow intervention affected marriage rates during 2017–8. In our data, 73% of respondents are married and 7% of singles get married in between the two waves. Column (1) of Table 5 shows a positive BenKnow treatment effect of 1.6 percentage points on the probability of being married in 2018, controlling for 2017 marital status. This appears driven by transition into marriage as the effect on being divorced in 2018 for those who were married in 2017 is small in magnitude and not statistically significant (column (2)). If we look at sexual activity by marital status, we see more abstinence within and outside marriage in the treatment group (Online Appendix Table C.5). In addition to its substantive relevance, the positive BenKnow treatment effect on marriage is another potential robustness check of our main result on self-reported sexual behaviour, as marriage is extremely unlikely to suffer from reporting bias and respondents are likely to perceive marriage as an HIV risk-reduction strategy.

### 4.2. BenKnow Treatment Effect on Revisions to Subjective Expectations

To better understand the mechanisms underlying the behavioural changes subsequent to the BenKnow intervention, our analyses in this section build on our theoretical model in Section 3 and utilise the extensive MLSFH-MAC data on probabilistic expectations.

Our analyses for the BenKnow treatment effect on subjective expectations are specified as follows. Let $\Delta y_{ij}=y_{ij(2018)}-y_{ij(2017)}$ be the revisions of expectation y between follow-up and baseline for individual i in village j. We estimate

(4) $$\Delta y_{ij}=\beta_{0}+\beta T_{j}+\sum_{s=1}^{S}\tau_{s}I_{j\in s}+X_{ij}\gamma +\varepsilon_{ij},$$

where $T_{j}$ is a dummy that equals 1 if village j is in the BenKnow treatment group and 0 otherwise, and β is the BenKnow treatment effect. The model also includes a vector of observed predetermined individual characteristics $X_{ij}$ (age group, gender and years of schooling),^25^ and fixed effects for the randomisation strata s (within-region village pairs; Bruhn and McKenzie, 2009), where $\tau_{s}$ denotes strata fixed effects, $I_{j\epsilon S}$ is an indicator for whether village j is in strata s and S is the total number of strata. Because the strata *s* are within the three MLSFH study regions, the strata dummies also control for all region-specific differences. SEs are clustered at the level of the randomisation, i.e., at the village level.

To investigate whether the respondents update their expectations towards the life-table survival probabilities provided during the information treatment, we also estimate

(5) $$\Delta y_{ij}=\beta_{0}+\beta_{1}T_{j}\times \;Perc\text{.}gap_{i}+\beta_{2}T_{j}+\beta_{3}\times \;Perc\text{.}gap_{i}+\sum_{s=1}^{S}\tau_{s}I_{j\in s}+{\text{X}}_{ij}\gamma +\varepsilon_{ij},$$

where $Perc\text{.}gap_{i}$ is the Benknow objective population survival probability—baseline subjective population survival of ‘someone like you’. A positive (negative) gap reflects an underestimation (overestimation) of prior beliefs relative to the objective measure. As discussed in Haaland *et al.* (2023), $\beta_{1}$ measures the extent of belief updating toward the provided probabilities among respondents in the treatment group; $\beta_{2}$ measures the average treatment effect on respondents’ beliefs to the extent it does not depend on individual priors; $\beta_{3}$ measures the extent to which changes in beliefs in the control group depend on the perception gap.

#### 4.2.1. Population survival

We start by testing whether the BenKnow treatment had a positive effect on population survival expectations (Hypothesis 1) using the specification presented in (4). Panel A of Table 6 documents a positive, sustained and statistically significant BenKnow treatment effect on the 2018 population-level survival probabilities for individuals who are healthy, are HIV+ or are sick with AIDS and on ART (columns (1), (2) and (4)). In the treatment group, all of these subjective population-level survival probabilities increase by approximately 4 percentage points, displaying a 6.1% increase relative to the baseline survival probabilities for healthy individuals, 6.6% for HIV+ individuals and 7.1% for individuals who are sick with AIDS and on ART. There is no treatment effect on the perceived survival of individuals who are sick with AIDS (not on ART), consistent with the fact that the BenKnow intervention videos emphasised the contributions of ART to recent increases in life expectancy. These results are important and suggest that respondents were able to understand and retain the information provided to them.

We also investigate whether the respondents update their expectations towards the life-table survival probabilities using the specification presented in (5). Panel B of Table 6 reveals that the coefficient $\beta_{2}$ associated with treatment $T_{j}$ is precisely estimated and of similar magnitude than in panel A, while the coefficient $\beta_{1}$ associated with $T_{j}\times \;Perc\text{.}gap_{i}$ is never precisely estimated, suggesting that individuals do not revise their beliefs according to their baseline perception gap. This is illustrated in Online Appendix Figure C.5 that shows non-parametric estimates of the follow-up survival expectations as a function of the baseline perception gap. We see that the mean revision of beliefs for the treatment group is larger than that of the control group for all values of the perceptions gap, consistent with a general positive treatment effect in the treatment group regardless of the baseline perception gap. These findings are relevant as they imply that the overall narrative of the BenKnow intervention about changing survival patterns in Malawi had more impact on individual’s revision of population survival expectations than the numeric life-table information about age- and gender-specific survival probabilities.

#### 4.2.2. Own survival

We now test whether the BenKnow treatment had a positive effect on own survival expectations (Hypotheses 2a and 2b). Column (6) of Table 6 shows the BenKnow treatment effect on the revisions to own survival expectations between 2017 and 2018 for the five-year time horizon. This is the analogue of the treatment effect on population survival probabilities since the variables are measured at the same waves and refer to the same survival horizon. Importantly, this treatment effect is ten times smaller than for healthy population survival, and imprecisely estimated (treatment effect for own survival is 0.004 (0.014) as opposed to 0.043 (0.011) for healthy population survival). We similarly find no treatment effect on the ten-year own survival expectations and on the survival expectations of ‘someone like you’ (Online Appendix Table C.6). Interactions of treatment with HIV status do not show statistically significant differences in revision (Online Appendix Table C.6).^26^

As discussed in our conceptual framework (Section 3), own survival expectations measured one year after the intervention may be influenced by both the BenKnow information and feedback effect from own behaviour. The negative treatment effect on risky sex (documented in Section 4.1) should have magnified the positive effect of the BenKnow intervention on own survival. Indeed, consistent with this behavioural change and as specified in Hypothesis 4, we do find a negative and precisely estimated treatment effect about the chance of being infected with HIV (column (1) of Table 7) from baseline to the 2018 follow-up.^27^ As of 2018, however, these revisions in the subjective probability of being HIV+ have not yet translated into gains in expected survival.

To gain a better understanding of the process through which respondents update own survival expectations in response to BenKnow, we can further leverage the expectations measured approximately two weeks after the BenKnow intervention. Within this short period, it is very unlikely that any feedback effect from behaviour on own survival beliefs has occurred, so the treatment effect on short-run revisions of survival expectations identifies the effect of the BenKnow information only. Similar to the findings for the long-run revisions of survival expectations measured in 2018, the last column in Table 6 reveals no treatment effect $\left(\text{coefficient }=0.016\;(0.013)\right)$. BenKnow neither affected short-run nor long-run revisions of own survival expectations. Our results are therefore not consistent with Hypotheses 2a and 2b.

We also investigate whether there are heterogeneous treatment effects depending on the baseline perception gap, using the specification from (5) as we have done for population survival expectations. However, panel B of Table 6 shows again no heterogeneity.

This limited effect of the intervention on own survival expectations is in contrast with the sustained revision of population survival expectations. One may wonder if there is more measurement error in own mortality expectations compared to population expectations, which may bias the treatment effect toward zero. The item non-response rate and the rate of answers equal 0.5, which are often thought of as a way of expressing epistemic uncertainty (Bruine de Bruin *et al.*, 2000), are useful signals of measurement errors in expectation data. We find a comparable rate of item non-response for expectations about own survival (4.8%) and healthy population survival (2.5%). Focal answers of 0.5 are only slightly more common for own survival (19.7% versus 16.8%). Moreover, we do not find that the intervention had an effect on the propensity to provide 0.5s or any focal answers (0, 0.5 and 1; see Online Appendix Table C.7). To account for measurement error, we also evaluate the treatment effect on a more crude measure of revisions using a categorical variable for revising downward (=−1), upward (= 1) or no revision (= 0). Online Appendix Table C.8 shows the results of ordered probit specifications where we are more or less flexible in the definition of no revision (e.g., no revision means that the difference between the prior and posterior is 0 in panel A and +/− 0.1 in panel B). As in Table 6, there is again a precise positive treatment effect for population survival expectations for healthy, HIV+ and on ART, but no precise treatment effect for own survival. Note also that there is no treatment effect for other measures of own survival we have collected, including respondent’s expected age at death (table not shown). All in all, the evidence is not strong for the idea that the difference in measurement error by expectation type is an important driver of the difference in treatment effects between own and population expectations.

We propose three possible explanations for the limited updating of own survival expectations. First, it may be related to the extent of private information about own health and behaviour, and hence own survival. Individuals with more private information have tighter priors about their own survival, and any new information would lead to only limited updating. Although we do not have direct evidence about the precision with which these beliefs are held, Online Appendix Figure C.3 shows that more respondents express certainty by reporting 0s and 1s for own survival compared to healthy population survival (24% versus 16%).^28^ Another plausible explanation is that people perceive their own mortality in God’s hands and beyond their control. In the 2018 wave, 90% of the respondents strongly agreed that ‘The events in their life unfold according to a divine or greater plan’. Moreover, narratives of death attributed to witchcraft are not uncommon (Ashforth and Watkins, 2015). Finally, a third explanation is that the intervention led respondents to believe that other individuals have behaviours different than they initially believed (e.g., healthy people seek regular health care; HIV-positive people are frequently on ART) without implication for their own survival. During the qualitative interviews, respondents made comments consistent with these three explanations.^29^

#### 4.2.3. HIV transmission risk

We now test whether the BenKnow treatment has a positive effect on the subjective probability of contracting HIV associated with having multiple partners (Hypothesis 3). Because HIV transmission risk expectations were not elicited in the 2012 and 2017 MLSFH mature adult surveys, these analyses use 2010 expectations as baseline. Column (4) of Table 7 documents a positive and precisely estimated treatment effect on the subjective probability that one would become HIV+ when having multiple sex partners. The magnitude of the treatment effect is 5 percentage points, or 6.5% of the average baseline beliefs.^30^ There is however no treatment effect on the subjective probability Π of contracting HIV if married to an HIV+ spouse (see the small and imprecisely estimated coefficient in column (3) of Table 7). This suggests that there has been no change in the perception of the ‘technology of transmission of HIV’ when holding constant the partner’s HIV status.^31^ Indeed, we did not anticipate the information on population survival leading to revision about Π. Moreover, the treatment effect on the probability of the spouse being HIV+ is negative, but not statistically significant (column (2)). Consistent with these results, there is positive treatment effect on $p^{1}\left(\Pi ,L^{HIV}\right)-p^{0}(\Pi )$ (column (5) of Table 7), which is what is relevant for behaviour, as emphasised in our theoretical framework.^32^

Because the HIV risk associated with having multiple partners $p^{1}\left(\Pi ,L^{HIV}\right)$ is an increasing function of both the HIV transmission technology Π and the local HIV prevalence $L^{HIV}$, the positive treatment effect on $p^{1}\left(\Pi ,L^{HIV}\right)$ combined with no treatment effect on Π suggests that, as hypothesised in Section 3, individuals perceive that the pool of potential sexual partners includes more HIV+ individuals, or has become ‘riskier’.

### 4.3. Discussion

Overall, our analyses of the BenKnow treatment effects on subjective expectations in Section 4.2 allow us to understand the mechanisms that lead individuals to adopt safer sexual behaviour. As anticipated, we find a positive treatment effect on population survival expectations for individuals that are healthy, HIV+ and on ART (Hypothesis 1). In contrast, however, our results do not support the hypothesis that this led to an improvement in own survival expectations (Hypotheses 2a and 2b). As a corollary, the negative treatment effect on risky sex cannot be driven by an overall improvement in own survival expectations. Instead, we establish a positive treatment effect on the perceived HIV transmission risk associated with risky sex (Hypothesis 3). Note however that it was not directly targeted by the intervention, and despite being already over-estimated prior to our intervention, it was further heightened by the BenKnow health information.

#### How did the BenKnow information lead to an update in the perceived HIV prevalence and HIV transmission risk?

There are several possible reasons. First, there is an actual increasing trend in HIV prevalence among mature adults in these communities, discussed in Section 2.2, which may have been made more salient by the intervention. Statements from the qualitative interviews point to the fact that some people are aware of this, sometimes attributing it to the availability of ART (‘Now that there is ART, more people are infected’). Second, there is evidence that individuals use information about one health outcome to make inference about other health outcomes.^33^ In particular, Malawians have been found to make the connection between local HIV prevalence and the number of people who had died from AIDS (Godlonton and Thornton, 2013). In our data, there is a positive correlation $\left(\rho =0.16^{***}\right)$ between the survival expectations of HIV+ individuals and the differential infection transmission risk of having multiple sexual partners, consistent with respondents seeing the link between the two variables.^34^ Finally, the increase in the survival of HIV+ persons might be particularly salient to respondents who may link it to HIV prevalence and HIV transmission risk, due to availability bias (Tversky and Kahneman, 1973) or the tendency to overestimate the probability of negative events (Harris *et al.*, 2009).

#### How are expectations about HIV transmission risk related to risky sexual behaviours?

An important conclusion from our analysis, made possible only by the availability of health expectation data, is that the reduction in risky sex caused by the BenKnow intervention appears to be driven primarily by the increase in perceived transmission risk associated with having multiple partners, i.e., the *transmission risk* mechanism. Subjective transmission risk has been found to have a causal impact on risky sex in the same local context (Delavande and Kohler, 2016), and in other SSA countries (Dupas, 2011a). Note that the decrease in risky sex, triggered by the increase in the perceived transmission risk, also led to a negative treatment effect on the probability of being HIV+ (Table 6). This lower probability of being HIV+, which encourages safe sex (Section 3.1), may have further magnified the negative effect on risky sex between the two waves.

We also analyse the direct impact of the subjective HIV transmission risk on risky sex in Online Appendix Table C.13 using an instrumental variable strategy, whereby the HIV transmission risk is instrumented with the indicator for the BenKnow treatment, assuming that the intervention has an effect on sexual behaviour only through HIV transmission risk. Consistent with our earlier results, the first stage is strong (column (1)). In the second stage (columns (2) to (4)), we find that respondents who expect a higher HIV transmission risk engage in safer sex practices, including marriage. In terms of magnitude, we find, for example, that an increase in the HIV transmission risk of 5 percentage points (which is the magnitude displayed in Table 3) increases abstinence by 2.5 percentage points (which is similar to what we see in Table 4).

Note that, although there is a positive treatment effect on the population survival expectations of HIV+ individuals, our intervention does not appear to have decreased the perceived cost of contracting HIV on average. As shown in Table 6, there is a positive treatment effect on the population survival expectations for HIV− individuals of similar magnitude. Indeed, we find no treatment effect on the individual-specific difference in survival by HIV status $\left(S_{2}^{-}-S_{2}^{+}\right)$. Moreover, there is evidence that individuals in SSA are uninformed of the fact that ART reduces transmission rates (Derksen and van Oosterhout, 2019; Bor *et al.*, 2021). In addition, living with ART does not seem an attractive option to all. In the qualitative interviews, a respondent says one should ‘abstain from sex because it is very hard to take medicine everyday’.

#### What are the effects on well-being?

In light of these findings on population survival expectations and risky sexual behaviours, one might wonder whether the BenKnow intervention had broader effects on well-being. Importantly, we do *not* find any treatment effect on subjective well-being, and scores of physical and mental health (Online Appendix Table C.13), suggesting that safer sexual activity did not reduce respondents’ well-being in the short/medium term (one year). We also find no effect on the frequency of sex, conditional on having sex (table not shown). Future follow-ups are necessary to show if the BenKnow intervention had long-term well-being implications.

#### How do the BenKnow augment our knowledge from Delavande and Kohler (2016)?

In Delavande and Kohler (2016), we estimated a simple structural model of risky sex using rich data on probabilistic beliefs from the MLSFH. Our simulations of different information interventions revealed that providing information on HIV transmission risks would actually increase the likelihood of having multiple partners. However, providing information on mortality risks would lead to a decrease in risky sexual behaviour, which motivated the design of the BenKnow intervention. To conduct these simulations, we made assumptions about how individuals would revise their beliefs based on the new information. We considered scenarios where the information campaign was either fully successful, resulting in individuals aligning their beliefs with the information, or partially successful, where individuals’ beliefs were a weighted average of the new information and their prior beliefs. One key benefit of this study is that we do not need to make these assumptions. Instead, we present evidence that changing specific beliefs is a complex and nuanced process. Not all expectations are equally malleable and expectations about various health risks are interconnected, indicating that even expectations not directly targeted by an intervention may be revised.

Going into the field also provided a reality check about what types of information interventions can be implemented in practice. In Delavande and Kohler (2016), we simulated an intervention providing information on survival risk conditional on HIV status. However, we found this difficult in practice and ultimately decided to convey *unconditional* survival risk. This was because obtaining reliable estimates of survival by HIV status for Malawi proved to be difficult.^35^ Additionally, even if we had this information, we believed that presenting both HIV+ and HIV− survival risk could potentially overwhelm respondents.

#### How do the BenKnow findings compare with other studies?

It is useful to compare the magnitudes of our treatment effects to other studies that examine somewhat comparable outcomes, although samples will always be different. Godlonton *et al.* (2016) estimated the effect of receiving information about the partial protection of male circumcision against HIV transmission risk among a sample of young men (average age of 32) in Malawi. They found essentially no effect among circumcised men, and safer sex practices among uncircumcised men measured one year after the intervention (for example, a 26% reduction in sex acts per month, a 17% decrease in the number of partners, a 65% increase in condom use, but no effect on abstinence). Our intervention led to a 22% decrease in the propensity to have multiple partners among males (using results from Online Appendix Table C.1), and a 6% reduction in the number of partners if we use an OLS specification similar to Godlonton *et al.* (2016).^36^ The age difference between the sample might explain the difference in the effect of abstinence (8% decrease in abstinence in the last year in our study). Kerwin (2018) focused on an intervention providing information about the (mostly overestimated) HIV transmission risk from unprotected sex with an infected partner in Malawi among younger respondents (average age of 29). About one month after the information provision, he found an average increase of 10% for having sex in the last week, although his results point to heterogeneity in behavioural response and evidence of fatalism among respondents who held high prior beliefs. Regarding pregnancy, Dupas (2011a) found that providing information in school on the relative risk of HIV infection by partners’ age led to a 28% decrease in teen pregnancy incidence (62% for pregnancies from relations with older partners) within a year in Kenya, which is larger than the 23% decrease in our sample of reproductive-age women over a two-year period.

#### Pre-analysis plan and current analysis.

Our study was registered with the AEA RCT registry (AEARCTR-0004965) on November 2019, after the completion of the data collection, but the registration includes our analysis plan as submitted to the University of Pennsylvania IRB in January 2017, prior to the first wave of data collection. In our pre-analysis plan (PAP), our primary outcomes include survival and disease perceptions (i.e., the subjective expectations from Section 4.2); health-related behaviours, including sexual risk-taking, smoking, alcohol consumption, health-care utilisation; physical and mental health and life-cycle behaviours such as savings, work efforts, investments in children. To give the paper a more narrow focus and message, we have decided to concentrate our analysis on sexual risk-taking and expectations. In an earlier version of this paper (Ciancio *et al.*, 2020, available online as a working paper), we included the following outcomes in addition to sexual behaviour: savings and investments, labour supply, income, expenditure on children (medical expenditure, school fees and clothes for children), alcohol and tobacco consumption, own expenditure (medical and clothes) and married as outcomes. Importantly, even when adjusting for multiple hypothesis testing, the analyses document statistically significant treatment effects at the 5% level on sexual behaviours. Although not our main focus in the present paper, Online Appendix Table C.13, discussed above, shows no treatment effect on subjective well-being, and scores of physical and mental health. The PAP also underlines the use of the theoretical model of Delavande and Kohler (2016) to understand the pathways of how updated expectations affect outcomes.

## 5. Conclusions

While the centrality of health-related expectations for a broad range of health and other life-cycle behaviours is undisputed, there is evidence that misperceptions about health and related risks are common in LICs. In particular, many individuals are overly pessimistic about survival risks. In such contexts, survival expectations are a potentially modifiable determinant of health behaviours that can be targeted by health interventions. Our BenKnow study among mature adults in rural Malawi provides the first RCT-based evidence about possibilities to (*i*) improve the accuracy of population survival expectations by providing information about current population mortality risks and (*ii*) test the hypothesis that more accurate expectations improve health decision-making. Importantly, BenKnow increased sexual abstinence, reduced the propensity of having multiple sex partners and increased marriage in a high-HIV environment. The magnitudes of these BenKnow treatment effects are conceptually plausible and substantively relevant.

Without data on expectations, we could only speculate about why individuals changed their sexual behaviour as a response to the new information. Specifically, we find a positive treatment effect one year after its implementation on population survival expectations for healthy individuals, HIV+ individuals and HIV+ individuals on ART. These population survival expectations turned out to be important inputs for the formation of other relevant health risks. In particular, and consistent with HIV+ people being thought to live longer, which implies an increase in HIV prevalence in the pool of potential sexual partners, we find a positive treatment effect on the HIV transmission risk associated with having multiple partners. This renders risky sexual behaviour more costly in terms of HIV infection risks. Note that this transmission risk was not targeted by the intervention, and was overestimated at baseline. Such a positive treatment effect is nevertheless consistent with the actual increase in HIV prevalence of older adults in our setting. However (and contrary to our own priors), the BenKnow intervention had a very limited effect on own survival expectations, in the short-term and after one year, plausibly because of private information about one’s own health status or traditional beliefs.

From a methodological point of view, our findings highlight the importance of incorporating detailed subjective expectation data in field experiments, as our study would not have been able to identify the pathways through which BenKnow affected behaviour in the absence of such data. The type of expectations should be disciplined by theoretical considerations and ideally pre-specified before data collection. Our study also illustrates that, even if a specific health-information intervention is effective in terms of affecting the hypothesised outcomes, the actual pathways through which the intervention affects these outcomes may be quite complex as expectations about health risks are interrelated and not equally malleable. Information about the pathways, however, is critical for assessing the scope of potential scale-up of interventions, and an understanding of mechanisms is essential for future fine-tuning of study designs and information of follow-up, replication and/or effectiveness studies.

Throughout our analysis, we have maintained the implicit assumption that individuals hold precise subjective probabilities. We acknowledge that this may be a strong assumption, especially in changing environments as the one we study. Respondents may instead exhibit deep uncertainty and hold imprecise beliefs. One potentially fruitful avenue would be to allow respondents to report imprecise expectations, stated as a range (Giustinelli *et al.*, 2022). We leave this possibility for future research.

From a policy point of view, our analyses lend support to the development and further testing of cost-effective health-information programs focused on population survival expectations. Such BenKnow-inspired interventions are highly pertinent in HIV-affected countries in sub-Saharan Africa, where mortality levels and disease conditions have changed swiftly and non-monotonically in recent years, and may extend to other areas where survival risks are likely distorted due to rapid changes in socioeconomic development or health, or in populations affected by major epidemics—including possibly COVID-19—or political upheavals.

## Supplementary Material

Supplementary Material / Online Appendix

Zipped Data - Replication File

## Figures and Tables

**Table 1.** Descriptive Statistics by Treatment Status.

	All respondents				HIV− respondents only		
	Mean (1)	Control (2)	Treated (3)	*p*-val (4)	Control (5)	Treated (6)	*p*-val (7)
Age	59.1	58.8	59.4	0.300	59.3	59.9	0.384
Male (%)	40.0	40.0	40.0	1	40.5	39.3	0.653
Married (%)	73.4	74.1	72.7	0.557	75.4	73.3	0.391
Divorced (%)	8.8	7.9	9.7	0.222	7.0	9.2	0.148
Widow (%)	17.8	18.0	17.6	0.821	17.6	17.5	0.958
Years of schooling	3.5	3.5	3.6	0.547	3.5	3.6	0.694
HIV+ (%)	7.5	6.3	8.7	0.088			
Expectations (%)							
Own survival (five years)	67.0	66.9	67.0	0.964	67.3	67.7	0.763
Own survival (ten years)	44.1	43.6	44.6	0.577	44.1	45.1	0.586
Pop. survival (healthy)	70.0	70.7	69.4	0.321	71.0	69.9	0.399
Pop. survival (HIV+)	62.0	63.1	60.9	0.093	63.7	61.6	0.123
Pop. survival (AIDS)	49.2	50.2	48.1	0.212	50.9	48.7	0.195
Pop. survival (ART)	56.9	57.7	56.1	0.266	58.4	56.6	0.275
‘Someone like you’ survival	69.0	68.8	69.2	0.746	69.0	69.2	0.859
HIV probability	18.6	17.1	20.1	0.022	14.6	15.9	0.253
HIV probability spouse	18.2	16.9	19.5	0.064	15.3	16.4	0.387
Sexual behaviour (%)							
No sex	35.5	34.2	36.8	0.294	34.0	37.4	0.195
Single partner	56.9	57.6	56.2	0.583	57.9	56.4	0.586
Multiple partners, condom	1.2	1.5	1.0	0.366	1.0	0.6	0.405
Multiple partners, no condom	6.3	6.7	6.0	0.591	7.0	5.5	0.255
Max observations	1,481	748	733		682	652	

*Notes:* The table presents summary statistics for the main variables used in the empirical analysis for the whole sample and separately by treatment group and for individuals tested negative for HIV. The variables refer to the 2017 baseline survey. Control and treatment show the mean for the BenKnow control and the treatment groups. Here *p*-val shows the *p*-value of a *t*-test where the null hypothesis is that the difference in means between the treatment and control groups is zero. Balance tests are done assuming independence across individuals. The first four columns refer to the whole sample while the last three columns refer to those tested negative for HIV during HTC.

**Table 2.** Model Notation.

Individual-specific subjective expectations P
*Survival* Own survival S Own survival conditional on being HIV+ $S^{+}$ Own survival conditional on being HIV− $S^{-}$ Population survival for HIV+ $S^{pop+}$ Population survival for HIV− $S^{pop-}$ *HIV status* Probability of being infected with HIV f Probability of spouse being infected with HIV $f^{s}$ *HIV transmission risks* Probability of contracting HIV if having regular sex with an HIV+ partner Π Perceived local HIV prevalence $L^{HIV}$ Probability of contracting HIV conditional on action a=0, $p^{0}=p^{0}(\Pi )=\Pi \times f^{s}$ Probability of contracting HIV conditional on action a=1, $p^{1}=p^{1}\left(\Pi ,L^{HIV}\right)=\Pi \times L^{HIV}$

**Table 3.** BenKnow Treatment Effects on Sexual Behaviour.

*Panel A: treatment effects*	Sexual risk index (SRI)		
	Had sex (1)	Number of partners (0,1,2+) (2)	Sex and condom (no sex, 1 partner, 2+ w/condom, 2+ w/o condom) (3)
BenKnow treatment	−0.140** (0.067)	−0.156*** (0.057)	−0.159*** (0.056)
Observations	1,479	1,479	1,479
*Panel B: predicted probabilities*	BenKnow assignment		Difference
	Control	Treatment	

No sex (%)	33.3	36.1	2.8 [0.3; 5.5]
Single partner (%)	57.9	56.4	−1.5 [−3.0; −0.2]
Multiple partners with condom (%)	1.3	1.1	−0.2 [−0.3; −0.02]
Multiple partners without condom (%)	7.6	6.4	−1.2 [−2.3; −0.1]

*Notes:* Panel A shows the coefficient of the BenKnow treatment effect using the ordered probit specification in (3). Sexual risk indices are defined as follows. Had sex: 0 = not sexually active in the last 12 months, 1 = sexually active in the last 12 months. Number of partners: 0 = not sexually active in the last 12 months, 1 = sex with spouse only, 2 = sex with multiple partners. Sex and condom: 0 = not sexually active in the last 12 months, 1 = sex with spouse only, 2 = sex with multiple partners and a condom at last intercourse, 3 = sex with multiple partners and no condom at last intercourse. Predicted probabilities in panel B for each of the four categories are based on column (3) of panel A. Panel B also shows the differences in predicted probabilities and the corresponding 95% confidence intervals calculated with bootstrap. All analyses additionally control for age group, gender, years of schooling and randomisation strata. SEs, reported in parentheses, are clustered at the village level.

** *p* < 0.05

*** *p* < 0.01.

**Table 4.** BenKnow Treatment Effects on Pregnancies or Recent Births among Women Aged < 45 Years.

	Respondent is pregnant or has baby aged *<* one year (1)	Respondent has infant aged 1–2 years (2)	Respondent has infant aged 3–4 years (3)
BenKnow treatment	−0.037** (0.016)	0.023 (0.023)	−0.001 (0.021)

Observations	1,022	1,022	1,022
Mean	0.158	0.163	0.194

*Notes:* The table shows regression coefficients for the BenKnow treatment effect on births and pregnancies using an OLS specification. The sample includes all women of reproductive age (*<*45) who participated in the 2019 MLSFH survey. The dependent variable in column (1) is a dummy for being currently pregnant or having a baby less than one year old at the time of the interview in the 2019 MLSFH survey. Dependent variables in columns (2) and (3) are dummy variables for having a baby of the specified age range. All analyses additionally control for age group, years of schooling and randomisation strata. SEs, reported in parentheses, are clustered at the village level. The mean of each dependent variable is shown at the bottom of the table.

** *p* < 0.05.

**Table 5.** BenKnow Treatment Effects on Marriage.

	Being married in 2018 (1)	Divorced in 2018 (2)
BenKnow treatment	0.016** (0.007)	0.003 (0.005)

Sample	All	Married in 2017
Observations	1,479	1,087
Mean	0.731	0.017

*Notes:* The table shows regression coefficients for the BenKnow treatment effect on the likelihood of being married. Estimates are based on a linear probability model. Outcome variable $y_{ij(2018)}$ is being married (yes/no) in 2018, controlling for marital status (married yes/now) $y_{ij(2017)}$ in 2017. Divorced includes divorced and separations. All analyses additionally control for age group, years of schooling and randomisation strata. SEs, reported in parentheses, are clustered at the village level. The mean of each dependent variable is shown at the bottom of the table.

** *p* < 0.05.

**Table 6.** BenKnow Treatment Effects on Survival Expectations.

	Probability of surviving for individuals who are				Own surv. prob.	
	Healthy (1)	HIV+ (2)	Sick with AIDS (3)	Sick with AIDS and on ART (4)	Long-run revision (5)	Short-run revision (6)
*Panel A: Main effect*						
BenKnow treatment	0.043*** (0.011)	0.041*** (0.013)	0.017 (0.016)	0.035** (0.014)	0.004 (0.014)	0.016 (0.013)

Observations	1,382	1,382	1,382	1,382	1,375	1,388

*Panel B: Interaction with perception gap*						
BenKnow treatment	0.055*** (0.016)	0.054*** (0.018)	0.023 (0.024)	0.031 (0.020)	0.002 (0.020)	0.003 (0.020)
BenKnow × perception gap	−0.068 (0.069)	−0.073 (0.085)	−0.028 (0.089)	0.029 (0.080)	−0.001 (0.077)	0.076 (0.088)
Perception gap	0.239*** (0.059)	0.278*** (0.065)	0.296*** (0.065)	0.144** (0.064)	0.156*** (0.043)	0.072 (0.072)

Observations	1,382	1,382	1,382	1,382	1,369	1,382

Mean	0.654	0.537	0.367	0.567	0.615	0.665

*Notes:* The table shows the coefficient of the BenKnow treatment effect on the updating of survival expectations from baseline to the 2018 follow-up. The first four subjective survival probabilities are based on questions about hypothetical individuals, of the same age and gender as the respondent, with the specified health status; see Section 2.4 for additional detail. The last two columns refer to five-year own survival. *Long* refers to the updating from baseline to follow-up, while *Short* refers to the update from baseline to the HTC stage. Perception gap is the difference between the objective survival probabilities presented in the BenKnow information intervention and the baseline subjective population survival of ‘someone like you’. All analyses additionally control for age group, gender, years of schooling and randomisation strata. SEs, reported in parentheses, are clustered at the village level. ‘Mean’ is the mean of the 2018 (follow-up) expectations.

** *p* < 0.05

*** *p* < 0.01.

**Table 7.** BenKnow Treatment Effect on Expectations about HIV Status and HIV Transmission Risks.

	Probability of being HIV+		Probability of HIV infection if sex with		$p^{1}-p^{0}$ (5)
	Own (1)	Spouse (2)	HIV+ partner (3)	Multiple partners (4)	
BenKnow treatment	−0.042*** (0.013)	−0.023 (0.018)	0.017 (0.020)	0.048*** (0.016)	0.053*** (0.016)

Observations	1,454	1,240	1,417	1,418	1,298
Mean	0.196	0.206	0.505	0.565	0.474

*Notes:* The table shows regression coefficients for the BenKnow treatment effect on the updating of beliefs from baseline to the 2018 follow-up. ‘Probability of being HIV+’ is the updating from 2017 to 2018 in the subjective probability of being currently HIV+ for the respondent and the respondent’s spouse. ‘HIV+ partner’ is the updating from the 2010 MLSFH survey to 2018 in the probability of someone of the respondent’s gender becoming infected with HIV when having sex with an HIV+ spouse over a year. ‘Multiple partners’ is the updating from the 2010 MLSFH survey to 2018 in the probability of someone of the respondent’s gender becoming infected with HIV if having sex with multiple partners over a year. Here $p^{1}-p^{0}$ is the updating in the difference in the probability of becoming infected with HIV when having multiple sex partners and having sex with a spouse only; $p^{0}=f^{s}\Pi$ is the product of the probability of the spouse being HIV+ at baseline and the transmission risk of having sex with an HIV+ partner. All analyses additionally control for age group, gender, years of schooling and randomisation strata. SEs, reported in parentheses, are clustered at the village level. ‘Mean’ is the mean of the 2018 (follow-up) expectations.

*** *p* < 0.01.

## Footnotes

1 We expect a priori our intervention to lead to an improvement in own survival from all causes of death as we communicated to the respondents a variety of factors for the gains in life expectancy. However, it is also possible for the perceived gains to be larger for non-HIV mortality risks than for HIV mortality risk. This would still encourage safe sex practices. In a competing mortality risk model, there is more to gain from HIV avoidance behaviour if non-HIV mortality is lower (Philipson and Posner, 1993; 1995; Dow *et al.*, 1999). Indeed, Oster (2012) found that the reduction in risky sex as a response to higher HIV prevalence is larger in places with lower malaria prevalence or maternal mortality.

2 Similar patterns have been documented as part of an emerging literature in India and the Philippines, among migrants in Nepal, and in some higher-income contexts (Delavande and Kohler, 2009; Delavande and Rohwedder, 2011; Bago d’Uva *et al.*, 2017; Delavande *et al.*, 2017; Capuno *et al.*, 2019; Shrestha, 2020).

3 While rarely done in health, it is more commonly done for education information intervention. See, for example, Jensen (2010).

4 Similarly complex inference by decision-makers has been evidenced in the context of ART roll-out or HIV testing. For example, ART availability significantly reduces expectations about mortality risk conditional on being HIV+ and perceived HIV infection risk (Baranov *et al.*, 2015). Also, finding out one’s and others’ HIV statuses has been found to have an implication on perceived HIV transmission risks (Delavande and Kohler, 2012) and the number of people suspected to have died from AIDS in one’s village (Godlonton and Thornton, 2013).

5 For a review of information provision experiment, see Haaland *et al.* (2023).

6 HIV prevalence is lower in rural areas (7.4%), where the MLSFH study population is based, as compared to urban areas (14.6%) (Malawi DHS, 2017).

7 UNAIDS AIDS info database, https://aidsinfo.unaids.org, accessed January 2020.

8 For instance, in 2008–10, when the MLSFH last asked the respective questions, the 2017 MLSFH mature adult reported a 63% HIV infection risk during a single intercourse without a condom with an HIV+ person, as compared to an accurate average risk of less than 0.5% (Boily *et al.*, 2009); MLSFH mature adults also reported a 77% annual probability of becoming infected when married to an HIV+ spouse, as compared to an accurate risk of about 12% (de Walque, 2007). MLSFH mature adults in 2010 also estimated an average local HIV prevalence in their communities of 37%, as compared to a prevalence among all MLSFH respondents of about 6% (Kohler *et al.*, 2015); mature adults also expected in 2010 a continued substantial increase in HIV prevalence within the next five years, which did not materialise as rural HIV prevalence remained approximately constant during 2010–6 (Malawi DHS, 2017). Note that in all MLSFH analyses for 2008–10, observations are taken from 2010, and if not available, from 2008; analyses included MLSFH mature adults who participated in the 2017 baseline survey for this study.

9 Only 60% of the 2012 and 82% of the 2017 HIV+ MLSFH mature adults are on antiretroviral treatment. Of the 2017 HIV+ mature adults, 43% have been on treatment for four years or less.

10 The key inclusion criteria in 2012 for enrolment in the MLSFH-MAC were two-fold: (*i* ) being an MLSFH respondent aged 45 years or older in 2012 and (*i i* ) having been interviewed in both the 2008 and 2010 MLSFH data collection rounds. The second criteria ensured that at least three waves of mental health and subjective well-being data were available for each baseline participant in 2012. Baseline enrolment in the MLSFH-MAC included 1,266 individuals clustered in 130+ villages, representing more than 90% of the 1,402 eligible MLSFH respondents who met the enrolment criteria (= target sample). Migration out of the study areas and mortality were the primary reasons for not enrolling eligible respondents. At each follow-up, the study population was augmented with additional MLSFH respondents who newly reached eligibility. To ensure an adequate representation of HIV+ individuals in the cohort, age-eligible HIV+ respondents were enrolled if they participated in either the 2008 *or* 2010 MLSFH data collection. Through the ongoing enrolment and migration follow-ups, the MLSFH-MAC cohort expanded to 1,257 respondents in 2013, 1,606 in 2017 and 1,532 in 2018. A detailed description of the data, including analyses of data quality and attrition, is provided in the MLSFH-MAC cohort profile (Kohler *et al.*, 2020).

11 In 2013 and 2017, the HTC team also screened for blood pressure before the HIV test and for blood sugar a day after. Those who were measured with high blood pressure or high blood sugar were given a referral card for seeking care. Around 17% of the respondents received this card. The share of respondents who got the referral card for the first time in 2017 is not statistically different between treatment and control (a *t*-test for equality of the means gives a *p*-value equal to 0.19).

12 Among the HIV+ individuals, very few tested positive for the first time. Out of 104 HIV+ respondents, only eight respondents tested positive for the first time in 2017 as part of our survey procedure and were not currently under ART (in 2017, in rural Malawi, ART as prevention was not implemented). Among the HIV− individuals, 55% report a strictly positive probability of being infected with HIV in 2017 prior to the HTC visit.

13 The attrition rate is 3.4% for control and 4.8% for treatment (*p*-value of a *t*-test for equality is 0.185 when clustering at the village level).

14 Life-table survival probabilities were obtained from the Global Burden of Disease Collaborative Network (GBD-Network, 2017; available from http://ghdx.healthdata.org/gbd-results-tool).

15 The higher presence of females in the sample is related to the original MLSFH survey design that, in 1998, sampled ever-married women and their spouses. While subsequent waves have expanded the MLSFH sample, the original sampling frame continues to result in an over-representation of women in the sample.

16 This interactive approach for eliciting expectations has been applied to several other contexts (Delavande *et al.*, 2011; 2017). Prior to 2017, respondents allocated up to ten beans, thus being able to state probabilities in 10-percentage-point increments. Peanuts, which can be split in half, were introduced in 2017 to allow respondents to express subjective probabilities in 5-percentage-point increments. This relatively coarse measurement of probabilities could be a problem for very low probability events. However, mortality risks of our respondents are not that small. As shown in Figure 1(a), 55-year-old (respectively 60-year-old) respondents have a 90% (respectively 85%) chance of surviving in the next five years. Moreover, the gap between objective risk and population survival expectations in the next five years is strictly larger than 5 percentage points for 76% of the respondents, and strictly larger than 10 percentage points for 65% of the respondents, suggesting that the overestimation is not an artefact of the elicitation format.

17 Prior validity studies suggest that, while the level of these risky behaviours is potentially to be misreported, the self-reported indicators of risky behaviour very likely discriminate between respondents with different levels of risky behaviours. Moreover, while a quasi-experimental design in which respondents were randomly allocated to one of three interviewing modes—face-to-face interviews, paper and pencil self-administered interviews and audio computer-assisted self-interviewing (audio-CASI)—documented significant differences in reported rates of premarital sex across interview modes (Mensch *et al.*, 2008), the analyses could not conclude a ranking of different methods in terms of measurement error. In particular, respondents reported twice as much sexual activity in the interviewer mode as in the audio-CASI mode, contradicting the hypotheses that interviewer-administrated survey questions result in underreporting of sexual behaviours.

18 The individual may further discount period 2 utility with a discount factor β, in which case $U^{-}$ and $U^{+}$ can be interpreted as discounted period 2 utility. The discount factor β cannot be separately identified from the U. Note also that risk aversion does not influence the decision here. The uncertainty pertains to a binary event such that risk aversion cannot be defined through a concave utility function since one cannot become only partially HIV+.

19 It is possible that BenKnow prompted additional information search about these types of events. The opportunities for individuals in rural Malawi to learn about these events is limited, so we expect such an effect to be small. In other contexts, these considerations may be important.

20 Because the HTC and baseline were separated by less than two weeks, it is reasonable to assume that there are not (yet) feedbacks from individual behaviour on own survival expectations.

21 More generally, assuming an $\alpha^{-}$ percentage increase for $S^{-}$, and an $\alpha^{+}$ percentage increase of $S^{+}$, the right-hand side of (2) will increase if $\alpha^{-}S^{-}U^{-}>\alpha^{+}S^{+}U^{+}$. It is also possible that we observe a decline in the relative survival that would induce an increase in risky behaviour. Given that the intervention emphasised that both healthy and HIV+ individuals live longer, our hypothesis is that any change in the relative survival should not be big enough to outweigh the effects of a longer life and a higher transmission risk.

22 We also estimate the same specification with the baseline data as an outcome to see whether there is any imbalance at baseline. The *p*-values are all above 0.4, showing no imbalance at baseline.

23 We conduct separate analyses for two different binary indicators of risky sex: (*i*) being sexually active and (*ii*) having multiple partners. Both specifications show a negative treatment effect, statistically significant at the 10% level. The magnitude of the BenKnow effect is larger than in the same specification without misreporting, suggesting that misreporting leads to a downward bias of the treatment effect.

24 The 2019 sample includes respondents less than 45 years old, plus some older respondents that were excluded from the original mature adult sample because they did not meet the MLSFH-MAC eligibility criteria of having completed both the 2008 and 2010 MLSFH surveys; see Kohler *et al.* (2020) for additional details.

25 The age-group dummies correspond to the same five-year age groups that were used in BenKnow to provide age- and gender-specific life-table survival information.

26 The interaction with HIV status is positive and large, but the small sample size of HIV+ individuals does not provide sufficient statistical power to find significant differences in updating.

27 The magnitude of the treatment effect of the expectations of being HIV+ is 4.2 percentage points, or 23% of the baseline belief. We do not find statistically significant differences in the treatment effect between those that tested HIV+ or HIV− (Online Appendix Table C.9). Similarly, Thornton (2008) and Delavande and Kohler (2012) found that learning about HIV status had limited effect on beliefs about HIV status among HIV+ individuals.

28 In another context, Wiswall and Zafar (2015) similarly found that the revision of beliefs about own future earning is somewhat inelastic to information about population earnings, suggesting that expectations about own earnings are not entirely linked to the type of public population information they provide: a 1% error (under-estimation of population earnings) is associated with only a 0.079% (precisely estimated) upwards revision of self-earnings. It is comparable to our treatment effect for own survival expectations being ten times smaller than for population expectations (Table 6).

29 For example, related to the first explanation, some would say when asked about their own survival ‘I know I am strong and take good care of myself’ or ‘I am old and very weak’, but might add about population survival ‘I do not know how people are in their body’. Related to the second explanation, some would say ‘I am old but still strong so just waiting on God’s will’. Related to the third explanation, respondents would comment about the videos, saying: ‘The old man is strong because he takes good care of himself’, ‘these days, there is still life for those who care for their lives’.

30 Note that the 2010 subjective transmission risks are slightly imbalanced between treatment and control groups (Online Appendix Table C.10). We therefore assess the robustness of the main results in the paper in two different specifications that ensure balance of the 2010 subjective transmission risks. First, we drop a village pair that cause most of the imbalance (Online Appendix Table C.10). Second, we re-weight the sample using entropy weights to balance the sample on transmission risk. In both cases, the treatment effects on expectations and sexual behaviour are very similar to those from our current specification (Online Appendix Table C.11).

31 Derksen and van Oosterhout (2019) showed that individuals in Malawi are relatively uninformed about the consequences of ART on viral load and its associated reduction in HIV transmission risk. Similar results are found from studies in South Africa, even among younger and more educated populations (Bor *et al.*, 2021).

32 Our theoretical framework emphasises that it is the relative transmission risk between risky and safe sex $\left(p^{1}-p^{0}\right)$ that matters for behaviour. We construct this as $p^{0}=f^{s}\times \Pi$, where $f^{s}$ is the probability of the spouse being HIV+ at baseline and Π is the transmission risk of having sex with an HIV+ partner.

33 Eriksson and Sovero (2016) found that, when a wife’s husband learns his negative HIV status, there is an upward revision in a woman’s subjective mortality risk, consistent with a woman believing that her husband has increased his risky behaviour post-testing, placing her at higher HIV risk. Delavande and Kohler (2012) found that among HIV-positive respondents, learning one’s status resulted in lower perceptions about HIV transmission risk, presumably because some of them also learned their partners were HIV negative.

34 Note that there is also a positive treatment effect on the survival of HIV− persons. Theoretically, as long as the percentage increase in $S_{\text{III}}^{pop\text{+}}$ is larger than the percentage increase in $S_{\text{III}}^{pop-}$, or if they both increase by the same value in percentage points (since $S_{\text{III}}^{pop+}<S_{\text{III}}^{pop-}$), the local HIV prevalence $L_{\text{III}}^{HIV}$ increases.

35 In Delavande and Kohler (2016), we used statistics provided by Todd *et al.* (2007), which measure survival since sero-conversion based on four East African population cohort studies *before* the availability of ART.

36 We find a treatment effect of −0.070 (0.035) if we run an OLS specification similar to (4) using the 2018 number of partners in the last 12 months as the outcome, controlling for the 2017 number of partners, and excluding the four males who report more than four partners.

## References

[R1] Armantier O, Nelson S, Topa G, Van der Klaauw W and Zafar B (2016). ‘The price is right: Updating inflation expectations in a randomized price information experiment’, Review of Economics and Statistics, vol. 98(3), pp. 503–23.

[R2] Armona L, Fuster A and Zafar B (2018). ‘Home price expectations and behaviour: Evidence from a randomized information experiment’, Review of Economic Studies, vol. 86(4), pp. 1371–410.

[R3] Ashforth A and Watkins S (2015). ‘Narratives of death in rural Malawi in the time of AIDS’, Africa, vol. 85(2), pp. 245–68.

[R4] Bago d’Uva T, O’Donnell O and van Doorslaer E (2017). ‘Who can predict their own demise? Heterogeneity in the accuracy and value of longevity expectations’, Journal of the Economics of Ageing, vol. 17, 100135.

[R5] Banerjee A and Duflo E (2011). Poor Economics: A Radical Rethinking of the Way to Fight Global Poverty, New York: Public Affairs.

[R6] Baranov V, Bennett D and Kohler HP (2015). ‘The indirect impact of antiretroviral therapy: Mortality risk, mental health, and HIV-negative labor supply’, Journal of Health Economics, vol. 44, pp. 195–211. 26516983 10.1016/j.jhealeco.2015.07.008 PMC4688176

[R7] Becker GS (1993). Human Capital, Chicago: University of Chicago Press.

[R8] Ben-Porath Y (1967). ‘The production of human capital and the life cycle of earnings’, Journal of Political Economy, vol. 75(4, Part 1), pp. 352–65.

[R9] Boily MC, Baggaley RF, Wang L, Masse B, White RG, Hayes RJ and Alary M (2009). ‘Heterosexual risk of HIV-1 infection per sexual act: Systematic review and meta-analysis of observational studies’, Lancet Infectious Diseases, vol. 9(2), pp. 118–29. 19179227 10.1016/S1473-3099(09)70021-0 PMC4467783

[R10] Bor J, Herbst AJ, Newell ML and Bärnighausen T (2013). ‘Increases in adult life expectancy in rural South Africa: Valuing the scale-up of HIV treatment’, Science, vol. 339(6122), pp. 961–5. 23430655 10.1126/science.1230413 PMC3860268

[R11] Bor J, Musakwa N, Onoya D and Evans D (2021). ‘Perceived efficacy of HIV treatment-as-prevention among university students in Johannesburg, South Africa’, Sexually Transmitted Infections, vol. 97(8), pp. 596–600. 34510009 10.1136/sextrans-2021-055031 PMC8606435

[R12] Brainerd E and Cutler DM (2005). ‘Autopsy on an empire: Understanding mortality in Russia and the former Soviet Union’, Journal of Economic Perspectives, vol. 19(1), pp. 107–30.

[R13] Bruhn M and McKenzie D (2009). ‘In pursuit of balance: Randomization in practice in development field experiments’, American Economic Journal: Applied Economics, vol. 1(4), pp. 200–32.

[R14] Bruine de Bruin W, Fischhoff B, Millstein SG and Halpern-Felsher BL (2000). ‘Verbal and numerical expressions of probability: “It’s a fifty–fifty chance” ’, Organizational Behavior and Human Decision Processes, vol. 81(1), pp. 115–31. 10631071 10.1006/obhd.1999.2868

[R15] Bruner JS (2009). Actual Minds, Possible Worlds, Cambridge, MA: Harvard University Press.

[R16] Capuno J, Kraft AD, Kudymowa E and O’Donnell O (2019). ‘Risk perceptions, optimism bias and information response: Evidence from a cardiovascular risk experiment in the Philippines’, in 2019 International Health Economics Association, Basel, Switzerland, 13–17 July.

[R17] Chinkhumba J, Godlonton S and Thornton R (2014). ‘The demand for medical male circumcision’, American Economic Journal: Applied Economics, vol. 6(2), pp. 152–77.

[R18] Ciancio A, Delavande A, Kohler HP and Kohler IV (2020). ‘Mortality risk information, survival expectations and sexual behaviors’, Working Paper 2020–39, University of Pennsylvania Population Center (PSC/PARC). 10.1093/ej/uead116 PMC11065140 38707864

[R19] de Walque D (2007). ‘Sero-discordant couples in five African countries: Implications for prevention strategies’, Population and Development Review, vol. 33(3), pp. 501–23.

[R20] Delavande A (2008a). ‘Measuring revisions to subjective expectations’, Journal of Risk and Uncertainty, vol. 36(1), pp. 43–82.

[R21] Delavande A (2008b). ‘Pill, patch, or shot? Subjective expectations and birth control choice’, International Economic Review, vol. 49(3), pp. 999–1042.

[R22] Delavande A (2014). ‘Probabilistic expectations in developing countries’, Annual Review of Economics, vol. 6(1), pp. 1–20.

[R23] Delavande A (2023). ‘Expectations in development economics’, in (Bachmann R, Topa G and van der Klaauw W, eds.), Handbook of Economic Expectations, pp. 261–91, Cambridge, MA: Academic Press.

[R24] Delavande A, Gine X and McKenzie D (2011). ‘Measuring subjective expectations in developing countries: A critical review and new evidence’, Journal of Development Economics, vol. 94, pp. 151–63.

[R25] Delavande A and Kohler HP (2009). ‘Subjective expectations in the context of HIV/AIDS in Malawi’, Demographic Research, vol. 20(31), pp. 817–74. 19946378 10.4054/DemRes.2009.20.31 PMC2784667

[R26] Delavande A and Kohler HP (2012). ‘The impact of HIV testing on subjective expectations and risky behavior in Malawi’, Demography, vol. 49(3), pp. 1011–36. 22744765 10.1007/s13524-012-0119-7 PMC3906596

[R27] Delavande A and Kohler HP (2016). ‘HIV/AIDS-related expectations and risky behavior in Malawi’, Review of Economic Studies, vol. 83(1), pp. 118–64.

[R28] Delavande A, Lee J and Menon S (2017). ‘Eliciting survival expectations of the elderly in low-income countries: Evidence from India’, Demography, vol. 54(2), pp. 673–99. 28281273 10.1007/s13524-017-0560-8 PMC5371617

[R29] Delavande A and Manski CF (2012). ‘Candidate preferences and expectations of election outcomes’, Proceedings of the National Academy of Sciences, vol. 109(10), pp. 3711–5. 10.1073/pnas.1200861109 PMC3309779 22355121

[R30] Delavande A and Rohwedder S (2011). ‘Differential survival in Europe and the United States: Estimates based on subjective probabilities of survival’, Demography, vol. 48(4), pp. 1377–400. 22042664 10.1007/s13524-011-0066-8 PMC3609718

[R31] Delavande A and Zafar B (2019). ‘University choice: The role of expected earnings, nonpecuniary outcomes, and financial constraints’, Journal of Political Economy, vol. 127(5), pp. 2343–93.

[R32] Derksen L and van Oosterhout J (2019). ‘Love in the time of HIV: Testing as a signal of risk’, Unpublished Manuscript, University of Toronto Mississauga.

[R33] Dow WH, Philipson TJ and Sala-i Martin X (1999). ‘Longevity complementarities under competing risks’, American Economic Review, vol. 89(5), pp. 1358–71.

[R34] Dupas P (2011a). ‘Do teenagers respond to HIV risk information? Evidence from a field experiment in Kenya’, American Economic Journal: Applied Economics, vol. 3(34), pp. 1–34.22199993

[R35] Dupas P (2011b). ‘Health behavior in developing countries’, Annual Review of Economics, vol. 3(1), pp. 425–49.

[R36] Dupas P and Miguel E (2017). ‘Impacts and determinants of health levels in low-income countries’, in (Banerjee AV and Duflo E, eds.), Handbook of Economic Field Experiments, vol. 2, pp. 3–93, Amsterdam: North-Holland.

[R37] Eriksson K and Sovero V (2016). ‘The impact of HIV testing on subjective mortality and investments in children: Experimental evidence from Malawi’, Economics Letters, vol. 149, pp. 90–3.

[R38] Freeman E and Anglewicz P (2012). ‘HIV prevalence and sexual behavior at older ages in rural Malawi’, International Journal of STD & AIDS, vol. 23(7), pp. 490–6. 22844003 10.1258/ijsa.2011.011340 PMC3788708

[R39] GBD Collaborators. (2018). ‘Global, regional, and national disability-adjusted life-years (DALYs) for 359 diseases and injuries and healthy life expectancy (HALE) for 195 countries and territories, 1990–2017: a systematic analysis for the Global Burden of Disease Study 2017’, Lancet, vol. 392(10159), pp. 1859–922. 30415748 10.1016/S0140-6736(18)32335-3 PMC6252083

[R40] GBD-Network. (2017). ‘Global burden of disease study 2016 (GBD 2016) results’, Seattle, United States: Institute for Health Metrics and Evaluation (IHME).

[R41] Giustinelli P (2016). ‘Group decision making with uncertain outcomes: Unpacking child–parent choice of the high school track’, International Economic Review, vol. 57(2), pp. 573–602.

[R42] Giustinelli P, Manski CF and Molinari F (2022). ‘Precise or imprecise probabilities? Evidence from survey response related to late-onset dementia’, Journal of the European Economic Association, vol. 20(1), pp. 187–221. 35185399 10.1093/jeea/jvab023 PMC8848333

[R43] Godlonton S, Munthali A and Thornton R (2016). ‘Responding to risk: Circumcision, information, and HIV prevention’, Review of Economics and Statistics, vol. 98(2), pp. 333–49.

[R44] Godlonton S and Thornton RL (2013). ‘Learning from others’ HIV testing: Updating beliefs and responding to risk’, American Economic Review, vol. 103(3), pp. 439–44. 25067844 10.1257/aer.103.3.439 PMC4108206

[R45] Greenwood J, Kircher P, Santos C and Tertilt M (2017). ‘The role of marriage in fighting HIV: A quantitative illustration for Malawi’, American Economic Review, vol. 107(5), pp. 158–62. 29553239 10.1257/aer.p20171056

[R46] Haaland I, Roth C and Wohlfart J (2023). ‘Designing information provision experiments’, Journal of Economic Literature, vol. 61(1), pp. 3–40.

[R47] Harris AJ, Corner A and Hahn U (2009). ‘Estimating the probability of negative events’, Cognition, vol. 110(1), pp. 51–64. 19036359 10.1016/j.cognition.2008.10.006

[R48] Hausman JA, Abrevaya J and Scott-Morton FM (1998). ‘Misclassification of the dependent variable in a discrete-response setting’, Journal of Econometrics, vol. 87(2), pp. 239–69.

[R49] Hornik R (2002). Public Health Communication: Evidence for Behavior Change, Mahwah, NJ: Lawrence Erlbaum Associates.

[R50] Hurd MD, Smith JP and Zissimopoulos JM (2004). ‘The effects of subjective survival on retirement and social security claiming’, Journal of Applied Econometrics, vol. 19(6), pp. 761–75.

[R51] Jayachandran S and Lleras-Muney A (2009). ‘Life expectancy and human capital investments: Evidence from maternal mortality declines’, Quarterly Journal of Economics, vol. 124(1), pp. 349–97.

[R52] Jensen R (2010). ‘The (perceived) returns to education and the demand for schooling’, The Quarterly Journal of Economics, vol. 125(2), pp. 515–48.

[R53] Kerwin JT (2018). ‘Scared straight or scared to death? The effect of risk beliefs on risky behaviors’, Unpublished Manuscript, University of Minnesota.

[R54] Kohler HP, Watkins SC, Behrman JR, Anglewicz P, Kohler IV, Thornton RL, Mkandawire J, Honde H, Hawara A, Chilima B, Bandawe C and Mwapasa V (2015). ‘Cohort profile: The Malawi Longitudinal Study of Families and Health (MLSFH)’, International Journal of Epidemiology, vol. 44(2), pp. 394–404. 24639448 10.1093/ije/dyu049 PMC4469793

[R55] Kohler IV, Bandawe C, Ciancio A, Kämpfen F, Payne C, Mwera J, Mkandawire J and Kohler HP (2020). ‘Cohort profile: The Mature Adults Cohort of the Malawi Longitudinal Study of Families and Health (MLSFH-MAC)’, BMJ Open, vol. 10, e038232. 10.1136/bmjopen-2020-038232 PMC7569924 33067285

[R56] Kremer M, Rao G and Schilbach F (2019). ‘Behavioral development economics’, in (Bernheim DB, DellaVigna S and Laibson D, eds.), Handbook of Behavioral Economics: Applications and Foundations 1, pp. 345–458, Amsterdam: North-Holland.

[R57] Lochner L (2007). ‘Individual perceptions of the criminal justice system’, American Economic Review, vol. 97(1), pp. 444–60.

[R58] Malawi DHS. (2017). Malawi Demographic and Health Survey 2015–16, Zomba, Malawi: National Statistical Office and ICF.

[R59] Manski CF (2004). ‘Measuring expectations’, Econometrica, vol. 72(5), pp. 1329–76.

[R60] McClelland RS, Richardson BA, Wanje GH, Graham SM, Mutunga E, Peshu N, Kiarie JN, Kurth AE and Jaoko W (2011). ‘Association between participant self-report and biological outcomes used to measure sexual risk behavior in HIV-1-seropositive female sex workers in Mombasa, Kenya’, Sexually Transmitted Diseases, vol. 38(5), pp. 429–33. 21217420 10.1097/OLQ.0b013e31820369f6 PMC3155001

[R61] McKenzie D, Gibson J and Stillman S (2013). ‘A land of milk and honey with streets paved with gold: Do emigrants have over-optimistic expectations about incomes abroad?’, Journal of Development Economics, vol. 102, pp. 116–27.

[R62] Mensch BS, Hewett PC, Gregory R and Helleringer S (2008). ‘Sexual behavior and STI/HIV status among adolescents in rural Malawi: An evaluation of the effect of interview mode on reporting’, Studies in Family Planning, vol. 39(4), pp. 321–34. 19248718 10.1111/j.1728-4465.2008.00178.x PMC2670342

[R63] Oster E (2012). ‘HIV and sexual behavior change: Why not Africa?’, Journal of Health Economics, vol. 31(1), pp. 35–49. 22277285 10.1016/j.jhealeco.2011.12.006

[R64] Oster E, Shoulson I and Dorsey ER (2013). ‘Limited life expectancy, human capital and health investments’, American Economic Review, vol. 103(5), pp. 1977–2002. 29533058 10.1257/aer.103.5.1977

[R65] Philipson TJ and Posner RA (1993). Private Choices and Public Health: The AIDS Epidemic in an Economic Perspective, Cambridge: Harvard University Press.

[R66] Philipson TJ and Posner RA (1995). ‘The microeconomics of the aids epidemic in Africa’, Population and Development Review, vol. 21, pp. 835–48.

[R67] Reniers G (2003). ‘Divorce and remarriage in rural Malawi’, Demographic Research, Special Collection 1, pp. 175–206.

[R68] Ruhm CJ (2016). ‘Health effects of economic crises’, Health Economics, vol. 25(S2), pp. 6–24. 10.1002/hec.3373 27870301

[R69] Schank RC and Berman TR (2002). ‘The pervasive role of stories in knowledge and action’, in (Green MC, Strange JJ and Brock TC, eds.), Narrative Impact: Social and Cognitive Foundations, pp. 287–313, Mahwah, NJ: Lawrence Erlbaum Associates.

[R70] Schöley J, Aburto JM, Kashnitsky I, Kniffka MS, Zhang L, Jaadla H, Dowd JB and Kashyap R (2022). ‘Life expectancy changes since COVID-19’, Nature Human Behaviour, vol. 6(12), pp. 1649–59. 10.1038/s41562-022-01450-3 PMC9755047 36253520

[R71] Shrestha M (2020). ‘Get rich or die tryin’: Perceived earnings, perceived mortality rates, and migration decisions of potential work migrants from Nepal’, World Bank Economic Review, vol. 34, pp. 1–27.

[R72] Thornton R (2008). ‘The demand for, and impact of, learning HIV status’, American Economic Review, vol. 98(5), pp. 1829–63. 21687831 10.1257/aer.98.5.1829 PMC3115776

[R73] Todd J, Glynn JR, Marston M, Lutalo T, Biraro S, Mwita W, Suriyanon V, Rangsin R, Nelson KE, Sonnenberg P, Fitzgerald D, Karita E and Zaba B (2007). ‘Time from HIV seroconversion to death: A collaborative analysis of eight studies in six low and middle-income countries before highly active antiretroviral therapy’, AIDS, vol. 21(Suppl 6), pp. S55–63. 10.1097/01.aids.0000299411.75269.e8 PMC5784803 18032940

[R74] Tversky A and Kahneman D (1973). ‘Availability: A heuristic for judging frequency and probability’, Cognitive Psychology, vol. 5(2), pp. 207–32.

[R75] UN Population Division. (2016). ‘Sub-Saharan Africa’s growing population of older persons’, Population Facts No. 2016/1. https://www.un.org/en/development/desa/population/publications/pdf/popfacts/PopFacts_2016-1.pdf (last accessed: December 2023).

[R76] UNAIDS. (2015). On the Fast-Track to End AIDS. UNAIDS 2016–2021 Strategy, Geneva: United Nations.

[R77] Van der Klaauw W (2012). ‘On the use of expectations data in estimating structural dynamic choice models’, Journal of Labor Economics, vol. 30(3), pp. 521–54.

[R78] Van der Klaauw W and Wolpin KI (2008). ‘Social security and the retirement and savings behavior of low-income households’, Journal of Econometrics, vol. 145(1–2), pp. 21–42. 21566719 10.1016/j.jeconom.2008.05.004 PMC3091488

[R79] Vollmer S, Harttgen K, Alfven T, Padayachy J, Ghys P and Bärnighausen T (2017). ‘The HIV epidemic in Sub-Saharan Africa is ageing: Evidence from the Demographic and Health Surveys in Sub-Saharan Africa’, AIDS and Behavior, vol. 21(1), pp. 101–13. 27837426 10.1007/s10461-016-1591-7

[R80] Wiswall M and Zafar B (2015). ‘Determinants of college major choice: Identification using an information experiment’, The Review of Economic Studies, vol. 82(2 ), pp. 791–824.

