# Supplementary material for: MORTALITY RISK INFORMATION, SURVIVAL EXPECTATIONS AND SEXUAL BEHAVIOURS
Source: Econ J (London). Author manuscript; Available in PMC 2025 May 1. (PMC11065140; doi:10.1093/ej/uead116)
Supplement: Supplementary Material / Online Appendix [file NIHMS1979496-supplement-Supplementary_Material___Online_Appendix.pdf]

Mortality Risk Information, Survival Expectations and Sexual Behaviors: Online Appendix

Alberto Ciancio[1], Adeline Delavande[2], Hans-Peter Kohler[3], and Iliana V. Kohler[3]

[1]University of Glasgow

[2]University of Technology Sydney

[3]University of Pennsylvania

May 31, 2023

A Online Appendix A: Expectations Questions

Section 12: Expectations Questions

INTERVIEWER: Recount the number of peanuts and check that you have 10 peanuts in the plate [__]. As you provide the explanation below, add the peanuts into the plate to illustrate what you say.

"I will ask you several questions about the chance or likelihood that certain events are going to happen. There are 10 peanuts in the cup. I would like you to choose some peanuts out of these 10 peanuts and put them in the plate to express what you think the likelihood or chance is of a specific event happening. One peanut represents one chance out of 10. If you do not put any peanuts in the plate, it means you are sure that the event will NOT happen. As you add peanuts, it means that you think the likelihood that the event happens increases. For example, if you put one or two peanuts, it means you think the event is not likely to happen but it is still possible. If you pick 5 peanuts, it means that it is just as likely it happens as it does not happen (fifty-fifty). If you pick 6 peanuts, it means the event is slightly more likely to happen than not to happen. If you put 10 peanuts in the plate, it means you are sure the event will happen. There is not right or wrong answer, I just want to know what you think.

Let me give you an example. Imagine that we are playing Bawo. Say, when asked about the chance that you will win, you put 7 peanuts in the plate. This means that you believe you would win 7 out of 10 games on average if we play for a long time. If you think that you will win slightly more than 7 games but less than 8 games on average, then you can break the peanut in half and put 7 ½ peanuts on the plate.

INTERVIEWER: Report for each question the NUMBER OF PEANUTS put in the PLATE. After each question, replace the peanuts in the cup (unless otherwise noted).

Interviewer: Remind respondent that he/she can put ½ bean if respondent wants to pick value between two whole peanuts (e.g., respondent thinks 1 and 1/2 peanuts (1.5) is the best answer). If respondent is not able to break the peanut in ½, help him/her with this.

For question X1: If respondent puts 10 (or 0) peanuts, prompt "Are you sure that this event will almost surely (not) happen?" CIRCLE 1 in column P if you prompted the respondent, and report the final answer only.

X1 *Pick the number of peanuts that reflects how likely you think it is that…*	# of peanuts in plate	Prompt for 0 or 10?
A person of your sex and age in your community will die within 5 years.	[____]	1

For the subsequent questions, no longer prompt for "0" and "10" answers

X2 *Pick the number of peanuts that reflects how likely you think it is that…*	# of peanuts in plate
a) *you are infected with HIV/AIDS now*	[____]
**b)** INTERVIEWER: for polygamous men, ask for most recent spouse<br>*your spouse or romantic partner is infected with HIV/AIDS now*<br>(INTERVIEWER: If no spouse or romantic partner, write 66)	[____]

X3 Consider a healthy man/woman in your village who currently does not have HIV. Pick the number of peanuts that reflects how likely you think it is that he will become infected with HIV...	# of peanuts in plate
c) *within the next 12 months if he/she is married to someone who is infected with HIV/AIDS*	[____]
d) *within the next 12 months if he/she has several sexual partners in addition to his/her spouse*	[____]

I want you to think how likely it is that you will die in the near future. We believe that there is nothing bad that will happen to you. But something bad might happen in the near future years to come, even though you prevent it to happen. If you don't want, you can refuse to answer these questions.

INTERVIEWER: If respondent refuses to answer, skip to GS1

Pick the number of peanuts that reflects how likely you think it is that you will:	# OF PEANUTS in plate
X7 *Pick the number of peanuts that reflects how likely you think it is that you*	
a) *will die within a five-year period beginning today*<br>(LEAVE PEANUTS ON PLATE)	[_____]<br>if 10 → SKIP to X8a
Add the number of peanuts that reflecs how likely you think it is that you:<br>b) *will die within a ten-year period beginning today*<br>(IT IS POSSIBLE TO ADD ZERO ADDITIONAL PEANUTS)	[_____]

Finally, I would like you to consider the likelihood that somebody else dies as time goes by. I am going to ask you about an imaginary person living in the same context like you, and I am going to describe him/her to you.

INTERVIEWER: For each of questions X8a to X8d start with an empty plate and 10 peanuts. Do not leave peanuts on plate.

Pick the number of peanuts that reflects how likely you think it is that one of the following persons will die within a five-year period beginning today:	# of peanuts in plate
X8a<br>For men:<br>*A man your age who is healthy and does not have HIV?*<br>For women:<br>*A woman your age who is healthy and does not have HIV?*	[_____]
X8b<br>For men:<br>*A man your age who is infected with HIV?*<br>For women:<br>*A woman your age who is infected with HIV?*	[_____]
X8c<br>For men:<br>*A man your age who sick with AIDS?*<br>For women:<br>*A woman your age who is sick with AIDS?*	[_____]
X8d<br>For men:<br>*A man your age who is sick with AIDS and who is treated with antiretroviral treatments (ART)?*<br>For women:<br>*A woman your age who "is" sick with AIDS and who is treated with antiretroviral treatments (ART)?*	[_____]

B Online Appendix B: Information Intervention

B.1 Statistical Information

Figure B.1: Benefits-of-Knowledge Health-information Intervention: Health information sheet providing life-table-based information about 5-year and 10-year mortality probabilities for a woman aged 60-64 years old.

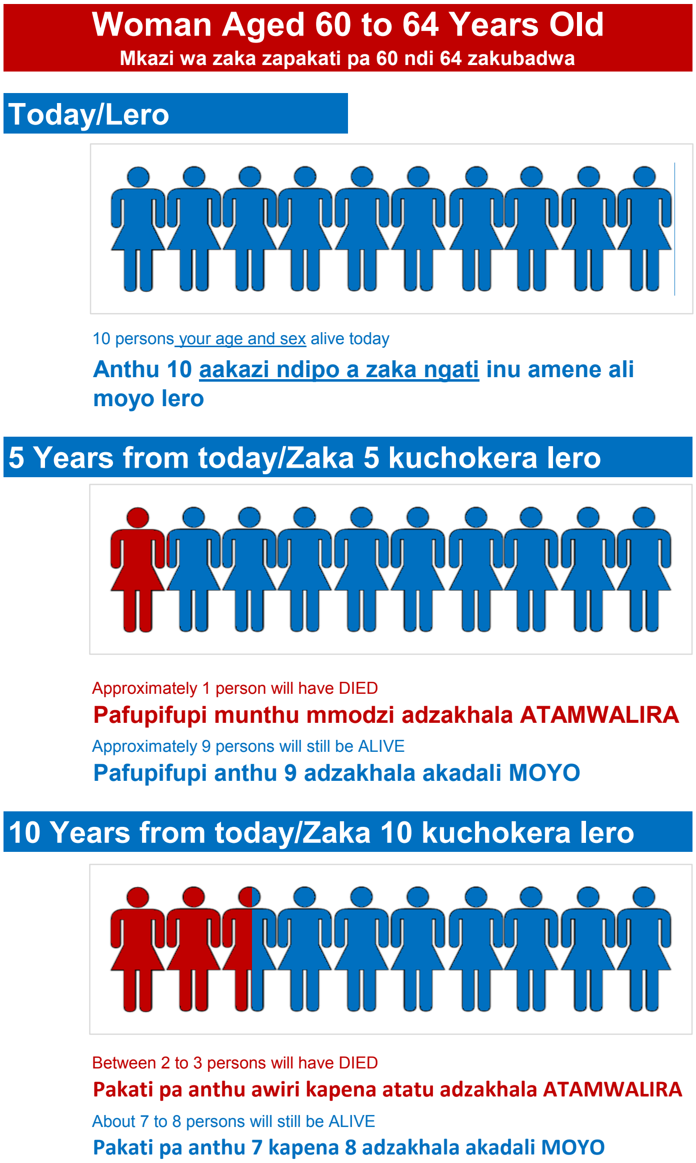

Table B.1: Life table probabilities of dying for BenKnow health-information intervention

	Probability of dying			
	Men		Women	
Age	within 5 years	within 10 years	within 5 years	within 10 years
< 45	0.06	0.13	0.04	0.08
45-49	0.07	0.15	0.05	0.1
50-54	0.08	0.18	0.06	0.13
55-59	0.1	0.23	0.07	0.17
60-64	0.14	0.31	0.11	0.25
65-69	0.2	0.43	0.16	0.37
70-74	0.28	0.58	0.24	0.53
75-79	0.41	0.71	0.38	0.68
80+	0.51	0.76	0.49	0.74

The table reports mortality probabilities for each demographic group that were conveyed during the Benefits-of-Knowledge Health-information Intervention using information sheets like the one shown in Figure B.1. Lifetable survival probabilities were obtained Global Burden of Disease Collaborative Network. Global Burden of Disease Study 2016 (GBD 2016) Results. Seattle, United States: Institute for Health Metrics and Evaluation (IHME), 2017. Available from http://ghdx.healthdata.org/gbd-results-tool

B.2 Intervention protocol

The Benefits of Knowledge: Mortality risk, Mental health and Life-cycle behavior

Protocol and Questionnaire for Health Information Intervention

Section 1---Background Information Pre-Intervention

When the survey team came to your house the other day, they asked you some questions about the chances that some people or you might die as time goes by using 10 peanuts.

**BK0** *Do you remember those questions?*	Yes……………….1<br>No…………………2

Let's look at your answers together.

INTERVIEWER: Verify the number of peanuts that respondent put when previously interviewed. Put the corresponding number of peanuts in the cup for 5 years probabilities and the corresponding number of peanuts in the cup for 10 years probabilities. Show the respondent the cup with [M9_X7A] peanuts for the 5 years probabilities and the cup with [M9_X7B] peanuts for the 10 years probabilities. Do not remove the peanuts from the cups and keep them in front of the respondent during the whole time of the interview!

*You allocated [M9_X7A] peanuts, meaning [M9_X7A] chances out of 10, when asked about the chances that you might die in the next **5 years**. [Interviewer: lay out M9_X7A peanuts for 5-year mortality risk on flat surface]*

*You allocated [M9_X7B] peanuts, meaning [M9_X7B] chances out of 10, when asked about the chances that you might die in the next **10 years**. [Interviewer lay out M9_X7B peanuts for 10-year mortality risk on flat surface, below the M9_X7A peanuts]*

**BK1** *Have you noticed lately that people in Malawi living in villages like yours tend to live longer than they used to 5 or 10 years ago?*	Yes ….. …………….1<br>No .... 2→ continue with videos following the exact sequence below; start with Video 1 (Story 1)
**BK2** *How did you notice that people tend to live longer than they used to 5 or 10 years ago?*<br>***[check all answers that apply]***<br>*Interviewer: probe if the respondent does not provide initially a response.*	I go to fewer funerals..1<br>I noticed that fewer of my friends and relatives are dying ....2<br>I notice that people are dying when they are older ………..3<br>AIDS treatment has become available nearby...4<br>Health services have improved, and this helps individuals...5<br>Other [________________________________]................6

Section 2---Videos

[CONTINUE WITH VIDEOS:]

I would like to show you a video showing that people in Malawi are living longer nowadays than 5 or 10 years ago. These videos have been recorded by ***actors*** *and the information in these videos is consistent with recent health and mortality trends in Malawi.*

Video 1 (Story 1---Davie the carpenter):

A middle-aged man, working it his carpenter's shop, talks: Hi, my name is Davie and I have a bit of land where I grow maize. I also know how to work with wood. I am lucky because both my parents are still alive. They are both in their 70ies and are doing well. They are taking care of themselves: they have enough food, they are in good health and they don't need to go often to the hospital and they actively participate in village activities. They also teach important things about life to me and my children. They knew that they could live longer than their parents and with the little they were earning they bought some livestock to support themselves in their old days. My brothers and I also help them sometimes. My aunties and uncle also died very old. They were more than 65. And I see a lot of other families in our village with old family members that are still alive. My grand-parents were not so lucky and they were dead when they were my age. Yes, I really notice that people are living longer nowadays. And it is a good thing for everyone.

Interviewer: continue with Video 2 --Rose

Video 2 (Story 2 -- Rose):

A middle-aged woman, working in her tailoring shop , talks: Hi, my name is Rose. I work in the field to plant cassava. When I have time, I do a bit of tailoring. I am married and I have four children who also help me in the field. The younger two go to school if they do not help at home. Five years ago, my husband got tested for HIV and he found out that he was HIV-positive. This was really a shock, and I was worried about the future of the family. How could we manage if my husband died soon? However, we have been lucky because my husband has had access to antiretroviral treatment (ART) in the local clinic. He takes his medicine regularly as the doctor explained him and I make sure he does not forget. He also often goes to the clinic for refill and check-ups. He looks really healthy and fit and does not show any sign of the disease. We do not know what will happen but we are very grateful for the availability of treatment. Ten years ago, my brother had HIV and he became very sick very quickly and died rapidly. Nowadays, there is more hope for people with HIV thanks to the availability of treatment. They can expect a longer life.

Interviewer: continue with Video 3 – the old man

Video 3 (Story 3 – old man):

An old man seating at home: I am lucky because I am more than 60 years old and I am still alive and feel healthy. I am not the only luck one. My neighbor next door is more than 70. And think about the popular musician Giddes Chalamanda. He is over 85 years old, and is still performing for the people. Last year, he even made is long-held dream of going to America come true, giving several shows across the USA. My parents were not so lucky because they died when they were in their 40ies. I think things are better nowadays. The kids, they do not die so frequently anymore. They get their immunization and many sleep under bed nets. They do not get sick so often. The adults, they do not die from HIV so rapidly anymore. The treatments, they really help. Also, people are not so hungry anymore and they eat more. When I was a kid, we were often hungry. My children and grand-children, they have almost always their meal on the table. It helps to build your health and keep you strong and prevent you from being unwell. Yes, things have changed quite a lot and people are less sick and live longer.

END OF VIDEO

Section 3--- Provision of Updated Mortality Information

[INTERVIEWER: SELECT THE MORTALITY INFORMATION SHEET CORRESPONDING TO THE RESPONDENT'S AGE AND SEX. USE THE INFORMATION ON THIS SHEET WHEN WE REFER TO 'MORTALITY INFO SHEET' BELOW]

Our research team has looked at some recent data showing how many individuals in Malawi are dying, and how long individuals your age and sex are likely to live. From these findings, it is possible to estimate how likely a person of your age and sex will die within five or ten years.

We would like to illustrate this to you with some pictures. In these pictures, blue persons indicate people who are alive, and red persons indicate people who have died.

*We begin with 10 hypothetical persons who are about your age and are of the same sex. These 10 persons are alive today, and they live in Malawi in a similar context as you do. You can see these 10 persons in this figure that shows 10 blue, or alive, persons [INTERVIEWER: SHOW **FIRST GRAPH** ON THE MORTALITY INFO SHEET].*

*We can now look five years into the future, and ask how many of the persons in the first figure will still be alive **5 years from today**. As you see on this picture [SHOW SECOND GRAPH "5 YEARS FROM TODAY" ON THE MORTALITY INFO SHEET], some of the persons will have died, and are shown in **red**, and others will still be alive, and are shown in **blue**, five years from today. How many persons are in red in this graph tells the chance out of 10 that a person your age and sex will die within the next **five** years: the more people we show in red (or the more red a person is), the higher is the risk of dying.*

Based on our knowledge today, we predict that [READ RED LINE IN 5-YEARS FROM TODAY SECTION] within 5 years from today, while [READ BLUE LINE IN 5-YEARS FROM TODAY SECTION] within 5 years from today.

*We can also look ten years into the future, starting today, and how many of the persons in the first figure will still be alive **10 years from today**. As you see on this picture [SHOW THIRD GRAPH "10 YEARS FROM TODAY" ON THE MORTALITY INFO SHEET], some of the persons will have died, and are shown in **red**, and others will still be alive, and are shown in **blue**, ten years from today. How many persons are in red in this graph tells you is the chance out of 10 that a person your age and sex will die within the next **ten** years. The more people we show in red (or the more red a person is), the higher is the risk of dying.*

Based on our knowledge today, we predict that [READ RED LINE IN "10-YEARS FROM TODAY" SECTION] within 10 years from today, while [READ BLUE LINE IN "10-YEARS FROM TODAY" SECTION] within 10 years from today.

Of course, nobody can predict what will happen to a specific individual, but this information can tell you about what is likely to happen if we look at a large group of people of your age and sex. And this information is helpful for you to think how likely you might die within the next 5 or 10 years. So, let's summarize this information: if we look at 10 persons your age and sex:

- *[READ RED LINE IN "5-YEARS FROM TODAY" SECTION] within 5 years from today, while [READ BLUE LINE IN "5-YEARS FROM TODAY" SECTION] within 5 years from today; and*
- *[READ RED LINE IN "10-YEARS FROM TODAY" SECTION] within 10 years from today, while [READ BLUE LINE IN "10-YEARS FROM TODAY" SECTION] within 10 years from today*

So based on this information, if I were to pick the number of peanuts that reflects how likely it is that a person your age and sex would die within 5 years, I would put **[INTERVIEWER: PICK THE NUMBER OF BEANS THAT CORRESPONDS TO THE NUMBER OF RED PEOPLE ON THE FIGURE WITH "5-YEARS FROM TODAY" MORTALITY INFO]** *peanuts on the plate.*
Interviewer: Put the number of beans infront of the cup with the 5-years chances of dying. Do not remove the peanuts but leave on the ground. So the respondent can see original answer in the cup, and new information on the ground until the end of the interview.

So based on this information, if I were to pick the number of peanuts that reflects how likely it is that a person your age and sex would die within 10 years, I would put **[INTERVIEWER: PICK THE NUMBER OF BEANS THAT CORRESPONDS TO**

THE NUMBER OF RED PEOPLE ON THE FIGURE WITH "10-YEARS FROM TODAY" MORTALITY INFO] *peanuts on the plate.*

Interviewer: Put the number of beans infront of the cup with the 5-years chances of dying. Do not remove the peanuts but leave on the ground. So the respondent can see original answer in the cup, and new information on the ground until the end of the interview.

[Interviewer: *The following is an example how to use ½ peanuts and whole peanuts if the figures are partially colored in red. 1) If the instructions on the mortality info sheet say "less than 1 person will have died" put ½ a peanut; 2) If the instructions on the mortality info sheet say "Between 2 and 3 persons will have died" or "About 2 and 3 persons will have died" then put 2½ peanut. In all other cases put a whole peanut (for example, if instructions say "almost [#] persons will have died", "about 1 person will have died", "approximately[#] persons will have died", "almost [#] persons will have died", "slightly more [#] persons will have died".*

**BK3:** *Do you understand this information?*	Yes ....1 → SKIP BK3a<br><br>No............2. → go back to beginning of Section 3 above, and explain again to respondent and ask BK3a;
**BK3a.** *Do you understand this information?*	Yes ....1<br><br>No.......................2
**BK3b.** Do you think this information reflects correctly what happens to people of your age and sex dying in your community nowadays?	Yes, reflects correctly...1<br>Yes, reflects somewhat..2<br>No, does not reflect correctly..3<br>Dont' Know...4

Of course, depending on your health and depending on your own family and economic context, you might be more or less likely to die than the average person your age and sex in a large group.

Now, I would like to ask you again about what you think about the chances that you might die in the next five or ten years. Look at the peanuts that you had put earlier for the chances that you will die within 5 years and 10 years. Based on what I have told you, and based on what you know about your own health, family and economic context please answer again the following questions below. Remember that you can break a peanut in ½ and put ½ peanut in addition to the whole peanuts if you want to pick a value between two whole peanuts.

Interviewer: Provide respondent with the empty 3rd cup in front of him/her. Give respondent 10 peanuts. Remind respondent that he/she can put ½ bean if respondent wants to pick value between two whole peanuts (e.g., respondent thinks 1 and 1/2 peanuts (1.5) is the best answer). If respondent is not able to break the peanut in ½, help him/her with this. If respondent used ½ peanut, do not substitute with a whole peanut.

Pick the number of peanuts that reflects how likely you think it is that you	**# OF PEANUTS in plate**

**BK_X7a:** *will die within a <u>five-year</u> period beginning today*<br><br>**(LEAVE PEANUTS ON PLATE)**	[_____]<br><br>**if 10 → ask BK_X8a, or BK_X8b, or BK_X8c, or BK_X8d and if the answer is yes and the respondent does not revise his/her answer then continue to BK4.**

**BK_X8a: If BK_X7a>M9_X7A:** Your answers show that you now think that the chance of dying within the next 5 years are larger than what you said before I gave you the information. Is that what you had in mind?	Yes 1<br>No 2<br><br>If No, go to **BK_X7a2**<br>If Yes, go to **BK_X7b**
**BK_X8b: If BK_X7a<M9_X7A:** Your answers show that you now think that the chance of dying within the next 5 years are smaller than what you said before I gave you the information. Is that what you had in mind?	Yes<br>No<br>If No, go to **BK_X7a2**<br>If Yes, go to **BK_X7b**
**BK_X8c: If BK_X7a=M9_X7A:** Your answers show that you now think that the chance of dying within the next 5 years are equal to what you said before I gave you the information. Is that what you had in mind?	Yes<br>No<br>If No, go to **BK_X7a2**<br>If Yes, go to **BK_X7b**
Pick the number of peanuts that reflects how likely you think it is that you	# OF PEANUTS in plate
**BK_X7a2:** will die within a five-year period beginning today<br><br>**(LEAVE PEANUTS ON PLATE)**	[_____]<br>if 10 go to BK4 or BK5 depending if they changed their answer compared to the initial number of peanuts in the main questionnaire

Add the number of peanuts that reflects how likely you think it is that you:<br><br>**BK_X7b.** *will die within a <u>ten-year</u> period beginning today*<br><br>**(IT IS POSSIBLE TO ADD ZERO ADDITIONAL PEANUTS)**	[_____]

**BK_X8d: If BK_X7b>M9_X7B:** Your answers show that you now think that the chance of dying within the next 10 years are larger than what you said before I gave you the information. Is that what you had in mind?	Yes<br>No<br>If No, go to **BK_X7b2**<br>If Yes, go to BK4
**BK_X8e: If BK_X7b<M9_X7B:** Your answers show that you now think that the chance of dying within the next 10 years are smaller than what you said before I gave you the information. Is that what you had in mind?	Yes<br>No<br>If No, go to **BK_X7b2**<br>If Yes, go to BK4
**BK_X8f: If BK_X7b=M9_X7B:** Your answers show that you now think that the chance of dying within the next 10 years are equal to what you said before I gave you the information. Is that what you had in mind?	Yes<br>No<br>If No, go to **BK_X7b2**<br>If Yes, go to BK4

Pick the number of peanuts that reflects how likely you think it is that you	**# OF PEANUTS in plate**
**BK_X7b2:** *will die within a ten-year period beginning today*<br><br>**(LEAVE PEANUTS ON PLATE)**	[____]<br><br>go to BK4 or BK5 depending if they changed their answer compared to the initial number of peanuts in the main questionnaire

Interviewer: Confirm if the respondent has changes the number of beans on the plate compared to his/her initial answer. If the respondent did NOT change his/her answer, continue with question BK4. If the respondent did change his/her answer, continue with question BK5.

**BK4** Why did you not want change your answer: **(select all that apply)**	I already knew that people live longer so I did not learn anything new …1<br>I do not believe the information you gave me ....2<br>The information you provided was not very clear .... 3<br>Nobody can predict their mortality.. 4<br>Other [______________________________].................................. 5
**BK5.** Why did you change your answer? **(select all that appy)**	I did not know that people live longer …………………………………… 1<br>I believe the information you gave me ……………………………………2<br>The information you provided to me was very convincing ………………3<br>Other ……………………………………………………………………………4

Finally, I would like you to consider the likelihood that somebody else dies as time goes by. I am going to ask you about an imaginary person living in the same context like you, and I am going to describe him/her to you.

INTERVIEWER: <u>Empty the 3rd cup infront of the respondent. For each of questions X8a to X8d start with an empty plate and 10 peanuts</u>. Do not leave peanuts on plate. If the respondent used ½ peanut, replace it after asking the question with one whole peanut and make sure that the respondent starts with 10 whole peanuts.

Pick the number of peanuts that reflects how likely you think it is that one of the following persons will die within a <u>five-year period</u> beginning today:	**# of peanuts in plate**
**BK_X8a**<br>**<u>For men:</u>**<br>*A man your age who is healthy and does not have HIV?*<br>**<u>For women:</u>**<br>*A woman your age who is healthy and does not have HIV?*	[_____]
**BK_X8b**<br>**<u>For men:</u>**<br>*A man your age who is infected with HIV?*<br>**<u>For women:</u>**<br>*A woman your age who is infected with HIV?*	[_____]
**BK_X8c**<br>**<u>For men:</u>**<br>*A man your age who sick with AIDS?*<br>**<u>For women:</u>**<br>*A woman your age who sick with AIDS?*	[_____]
**BK_X8d**<br>**<u>For men:</u>**<br>*A man your age who sick with AIDS and who is treated with antiretroviral treatments (ART)?*<br>**<u>For women:</u>**<br>*A woman your age who sick with AIDS and who is treated with antiretroviral treatments (ART)?*	[_____]

C Online Appendix C: Additional Tables and Figures

Figure C.1: Sexual behaviours and sexual risk taking among MLSFH mature adults

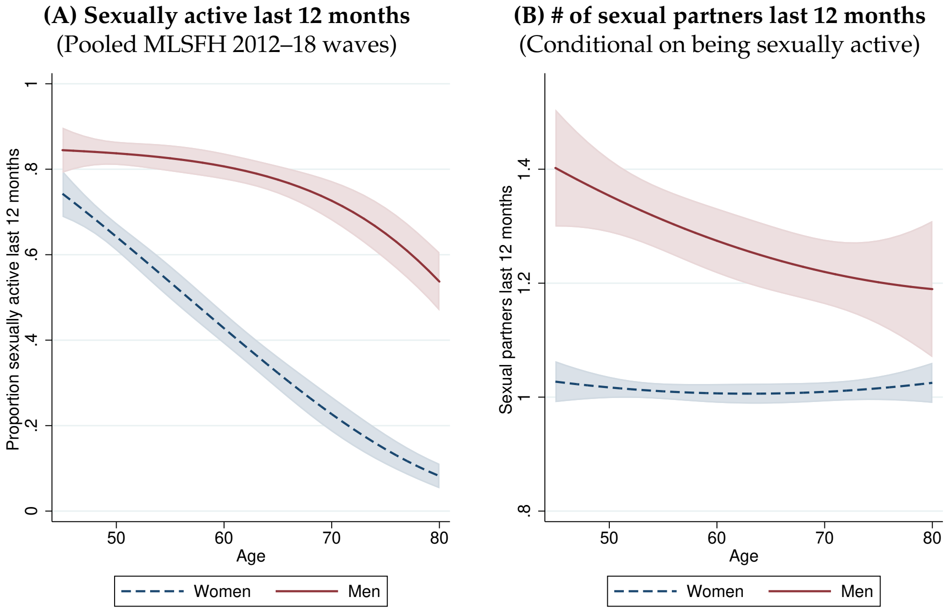

Notes: Marginal means (with 95% confidence intervals) obtained by regressing the outcome variables, sexual active in last 12 months (Panel A) and number of sexual partners in last 12 months (Panel B) on a quadratic function of age, separately by sex. Analyses are pooled across the 2012, 2013, 2017 and 2018 MLSFH mature adults surveys.

Figure C.2: HIV Prevalence by age group from DHS data.

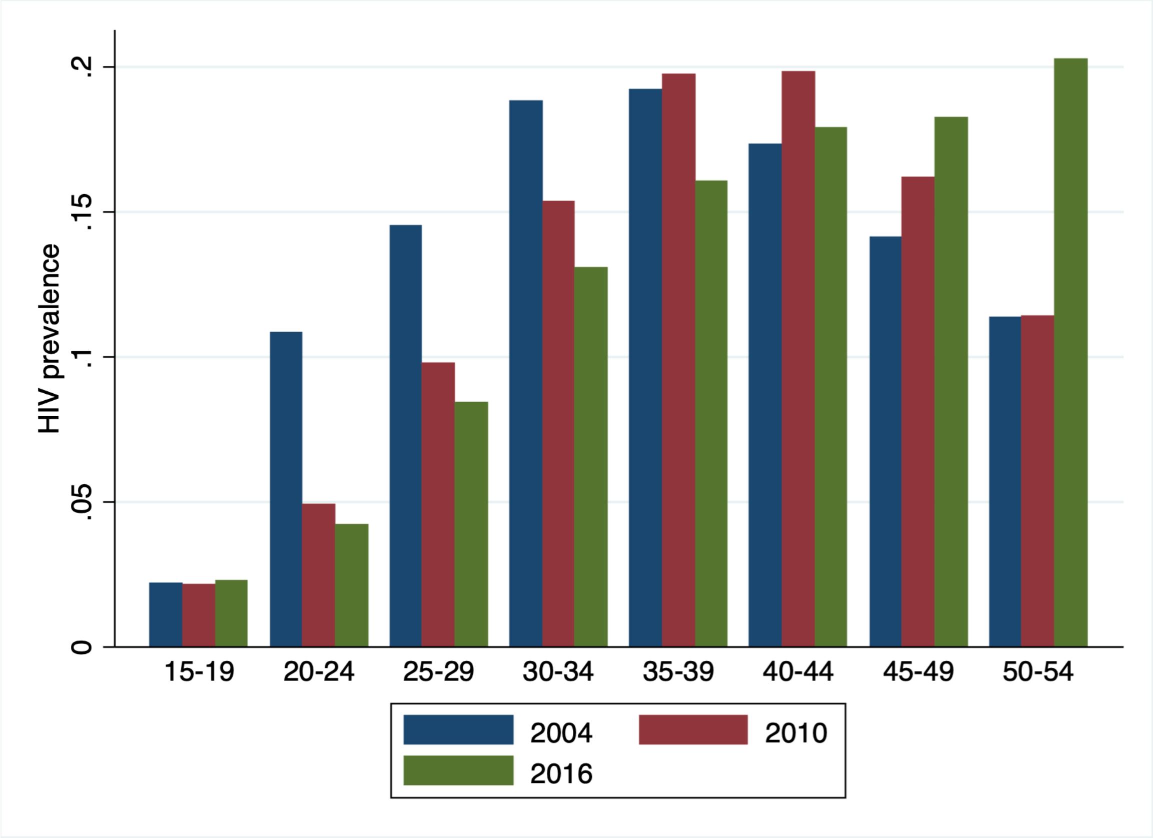

The figure shows HIV prevalence from three waves of the Demographic and Health Surveys (DHS) for Malawi. The DHS interviews women and men 15-54 years old and is meant to be nationally representative.

Figure C.3: Subjective survival probabilities at baseline.

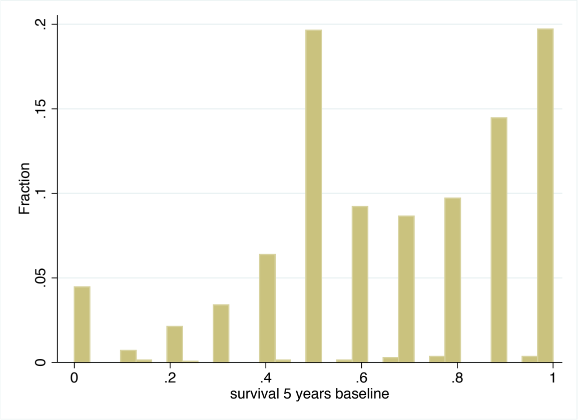

(a) Own

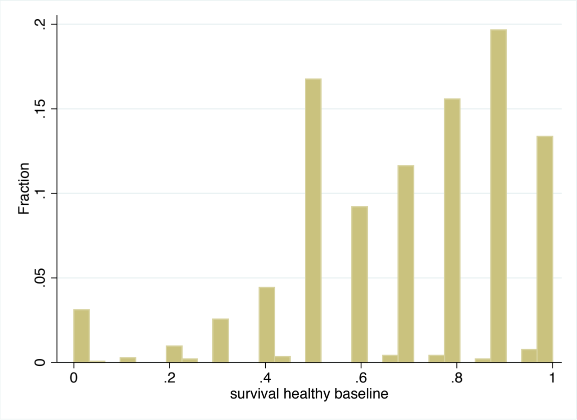

(b) Healthy

Panel (a) shows a histogram of the 5-year own subjective survival probability at the 2017 Intervention baseline. Panel (b) shows a histogram of the 5-year population subjective survival probability for healthy individuals at the 2017 Intervention baseline.

Figure C.4: Predictive power of own subjective survival probabilities.

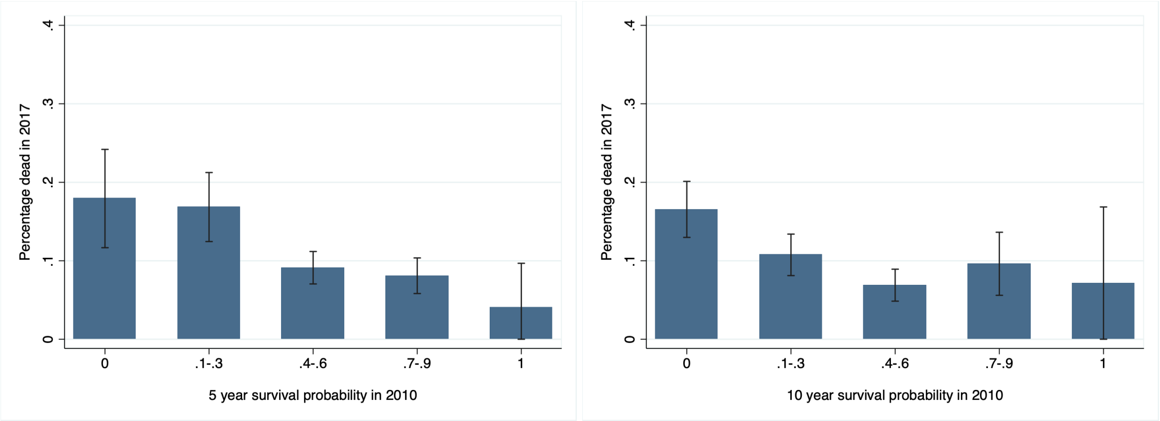

(a) 5 years **(b) 10 years**

The figures show the percentage of respondents who are dead in 2017 by different levels of subjective own survival probabilities elicited in 2010. The left figure uses 5 year survival probabilities while the right figure uses 10 year survival probabilities.

Figure C.5: Population survival expectations at follow-up by baseline perception gap

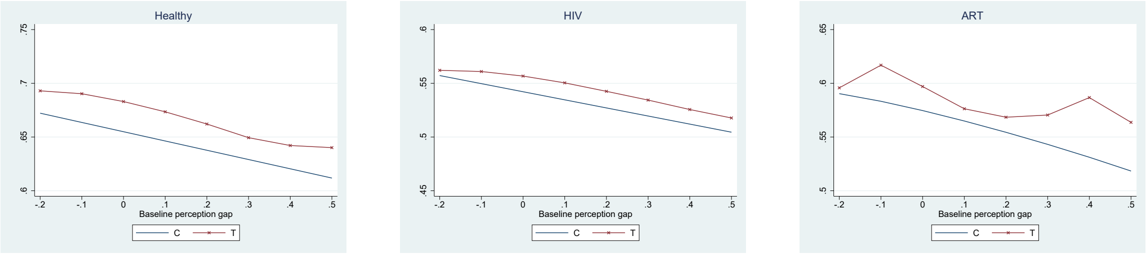

(a) Healthy **(b) HIV** **(c) ART**

Notes: The graph shows nonparametric estimates of the 2018 mean population survival expectation for individuals who are healthy (panel a), HIV+ (panel b) and sick with AIDS and on ART (panel c) using an Epanechnikov kernel function with a cross-validated optimal bandwidth. C refers to the control group and T to the treatment group. Baseline perception gap is defined as the difference between objective survival rates used in the BenKnow intervention and the baseline subjective population survival of "someone like you."

Table C.1: Predicted probabilities of sexual risk taking, by BenKnow assignment: subgroups

HIV– only	BenKnow Assignment		
	Control	Treatment	Difference
No sex (SRI3 = 0)	.334	.356	.022
Single partner (SRI3 = 1)	.581	.569	-.012
Multiple partners with condom (SRI3 = 2)	.01	.009	-.001
Multiple partners without condom (SRI3 = 3)	.075	.066	-.009

Men only	BenKnow Assignment		
	Control	Treatment	Difference
No sex (SRI3 = 0)	.166	.206	.04
Single partner (SRI3 = 1)	.685	.679	-.006
Multiple partners with condom (SRI3 = 2)	.025	.021	-.004
Multiple partners without condom (SRI3 = 3)	.124	.095	-.029

Women only	BenKnow Assignment		
	Control	Treatment	Difference
No sex (SRI3 = 0)	.373	.409	.036
Single partner (SRI3 = 1)	.617	.584	-.033
Multiple partners with condom (SRI3 = 2)	.002	.001	-.001
Multiple partners without condom (SRI3 = 3)	.009	.006	-.003

Notes: The table shows the predicted probabilities of being in each risky sex state calculated using the ordered probit model with four different states separately for selected subgroups. The top panel includes only HIV- respondents, the mid panel include only men and the bottom panel includes only women.

Table C.2: BenKnow treatment effects on sexual behaviours: HIV status interactions

	Sexual Risk Index (SRI)					
	Had sex		Number of partners (0,1,2+)		Sex and condom (no sex, 1 partner, 2+ w/ condom, 2+ w/o condom)	
	(1)	(2)	(3)	(4)	(5)	(6)
BenKnow treatment	-0.140**	-0.136*	-0.156***	-0.135**	-0.159***	-0.136**
	(0.067)	(0.077)	(0.057)	(0.067)	(0.056)	(0.066)
HIV+		0.007		0.168		0.149
		(0.343)		(0.268)		(0.264)
BenKnow treatment × HIV+		-0.253		-0.445		-0.421
		(0.408)		(0.350)		(0.337)
Observations	1,479	1,440	1,479	1,440	1,479	1,440

Notes: The table shows regression coefficients for the BenKnow treatment effect on risky sexual behaviour using an ordered probit specification. Estimates are based on (ordered) probit specification in Eq. (3). Sexual Risk Indices are defined as: Had Sex: 0 = not sexually active in the last 12 months, 1 = sexually active in the last 12 months; Number of Partners: 0 = not sexually active in the last 12 months, 1 = sex with spouse only, 2 = sex with multiple partners; Sex and Condom: 0 = not sexually active in the last 12 months, 1 = sex with spouse only, 2 = sex with multiple partners and condom at last intercourse, 3 = sex with multiple partners and no condom at last intercourse. All analyses additionally control for age group, years of schooling and randomization strata. Standard errors are clustered at the village level.

Table C.3: BenKnow treatment effects on sexual behaviours: excluding polygamous men

	Sexual Risk Index (SRI)		
	(1) Had sex	(2) Number of partners (0,1,2+)	(3) Sex and condom (no sex, 1 partner, 2+ w/ condom, 2+ w/o condom)
BenKnow treatment	-0.140**	-0.133**	-0.135**
	(0.070)	(0.062)	(0.061)
Observations	1380	1380	1380

Notes: The table shows regression coefficients for the BenKnow treatment effect on risky sexual behaviour using an ordered probit specification for individuals who are not polygamous. Estimates are based on (ordered) probit specification in Eq. (3). Sexual Risk Indices are defined as: Had Sex: 0 = not sexually active in the last 12 months, 1 = sexually active in the last 12 months; Number of Partners: 0 = not sexually active in the last 12 months, 1 = sex with spouse only, 2 = sex with multiple partners; Sex and Condom: 0 = not sexually active in the last 12 months, 1 = sex with spouse only, 2 = sex with multiple partners and condom at last intercourse, 3 = sex with multiple partners and no condom at last intercourse. All analyses additionally control for age group, years of schooling and randomization strata. Standard errors are clustered at the village level.

Table C.4: BenKnow treatment effects on sexual behaviours: Binary outcomes

	(1) sex active	(2) multiple partners	(3) multiple partners ‖ sex	(4) condom ‖ sex	(5) condom ‖ multiple partners
BenKnow treatment	-0.022**	-0.021**	-0.028**	0.018	0.054
	(0.011)	(0.009)	(0.012)	(0.017)	(0.132)
Control mean	0.676	0.094	0.139	0.107	0.143
Observations	1470	1311	874	765	46

Notes: The table shows regression coefficients for the BenKnow treatment effect on risky sexual behaviours using binary outcomes only. Estimates are based on probit specification in Eq. (3). Multiple partners refers to having sex with multiple partners in the last 12 months. Condom refers to condom use at last intercourse. In column 1, we control for being sexually active at baseline. In columns 2-3, we control for being sexually active and having multiple partners at baseline. In the last 2 columns we control for being sexually active and condom use at baseline. In columns 3 and 4, we restrict the sample to those who are sexually active at follow-up. In column 5, we restrict the sample to those who have multiple sexual partners at follow-up. All analyses additionally control for age group, years of schooling and randomization strata. Standard errors are clustered at the village level.

Table C.5: Predicted probabilities of sexual risk taking and marriage, by BenKnow assignment

	BenKnow Assignment		
	Control	Treatment	Difference
Married, no sex	.093	.107	.014
Single, no sex	.239	.248	0.09
Married, sex	.630	.631	.001
Single, sex	.038	.014	-.024

Notes: The table shows the predicted probabilities of being in each marriage and sex state calculated using a multinomial logit model with four different states for selected subgroups. Standard errors are clustered at the village level.

Table C.6: BenKnow treatment effects on own survival expectations

	Subjective probability of surviving				
	**Long run** (measured in 2018)			**Short run** (measured post-intervention 2017)	
	someone like you 5 years	own 5 years	own 10 years	own 5 years	own 10 years
	(1)	(2)	(3)	(4)	(5)
Panel A					
BenKnow treatment	-0.012	0.004	0.018	0.016	0.014
	(0.011)	(0.014)	(0.016)	(0.013)	(0.016)
Observations	1382	1375	1375	1388	1388
Panel B					
BenKnow treatment	-0.017	-0.008	0.007	0.019	0.028
	(0.012)	(0.014)	(0.016)	(0.014)	(0.017)
HIV+	-0.035	-0.070	-0.023	-0.003	0.059
	(0.048)	(0.052)	(0.065)	(0.041)	(0.050)
Treatment × HIV+	0.049	0.096	0.042	-0.002	-0.102
	(0.059)	(0.064)	(0.080)	(0.061)	(0.080)
Observations	1348	1340	1340	1366	1366

Notes: The table shows regression coefficients for the BenKnow treatment effect on own subjective survival probabilities. In the first three columns, the dependent variables are the updating of each probability from baseline to the 2018 follow-up. In the last two columns, the dependent variables are the update of each probability from baseline to the HTC stage. HIV+ is a dummy for being tested positive during the HTC exercise. Someone like you refers to the survival of "a person of your sex and age in your community". All analyses additionally control for age group, years of schooling and randomization strata. Standard errors are clustered at the village level.

Table C.7: BenKnow treatment effects on focal answers for own survival expectations

	(1) missing	(2) focal: 0%, 50%, 100%	(3) focal: 50%	(4) focal: 0%, 100%
BenKnow treatment	-0.006	-0.000	-0.001	0.001
	(0.006)	(0.022)	(0.019)	(0.015)
Observations	1479	1380	1380	1380

Notes: The table shows regression coefficients for the BenKnow treatment effect on missing and focal answers for own subjective survival probabilities. All analyses additionally control for age group, years of schooling and randomization strata. Standard errors are clustered at the village level.

Table C.8: BenKnow treatment effects on survival expectations: Categorical update

	Own survival		Population survival			
	Long	Short	Healthy	HIV+	AIDS	ART
	(1)	(2)	(3)	(4)	(5)	(6)
Panel A						
BenKnow treatment	-0.017	0.015	0.064*	0.158***	0.048	0.108**
	(0.043)	(0.047)	(0.038)	(0.052)	(0.051)	(0.042)
Observations	1382	1393	1423	1420	1421	1417
Panel B						
BenKnow treatment	-0.006	0.029	0.097***	0.151***	0.033	0.078*
	(0.041)	(0.045)	(0.037)	(0.051)	(0.046)	(0.041)
Observations	1382	1393	1423	1420	1421	1417

Notes: The table shows regression coefficients for the BenKnow treatment effect on subjective survival probabilities. Dependent variables are categorical: -1 for revising downward, 1 for revising upward, 0 for no revision. In Panel B downward and upward revisions require a more than 10 percentage points change in expectations. The models are ordered probit. All analyses additionally control for age group, years of schooling and randomization strata. Standard errors are clustered at the village level.

Table C.9: BenKnow treatment effect on expectations about being HIV+ (in 2018) and expectations about HIV transmission conditional on sexual behaviours

	Probability of being HIV+		Prob. of contracting HIV if sex with HIV+ partner		Prob. of contracting HIV if sex with multiple partners	
	(1)	(2)	(3)	(4)	(5)	(6)
BenKnow treatment	-0.042***	-0.034**	0.017	0.019	0.048***	0.039**
	(0.013)	(0.014)	(0.020)	(0.020)	(0.016)	(0.017)
HIV+		0.002		0.043		0.020
		(0.070)		(0.055)		(0.063)
BenKnow treatment × HIV+		-0.070		-0.105		0.039
		(0.084)		(0.075)		(0.072)
Observations	1454	1417	1417	1383	1418	1384

Notes: The table shows regression coefficients for the BenKnow treatment effect on the updating of beliefs over HIV-related probabilities from baseline to the 2018 follow-up. HIV probability is the subjective probability of being currently HIV+. HIV+ is a dummy for being tested positive during the HTC exercise. HIV+ partner is the update from baseline MLSFH survey in 2010 to the follow-up survey in 2018 in the probability of becoming infected with HIV having sex with an HIV+ spouse over a year. Multiple parrtners is the update from baseline MLSFH survey in 2010 to the follow-up survey in 2018 in the probability of becoming infected with HIV having sex with multiple partners over a year. All analyses additionally control for age group, years of schooling and randomization strata. Standard errors are clustered at the village level.

Table C.10: Balance in 2010 perceived HIV transmission risk

	Control	Obs	Treatment	Obs	P-value
Panel A: all respondents					
Sex with spouse	0.787	731	0.762	705	0.033
Sex with multiple partners	0.760	731	0.731	704	0.007
Panel B: drop a pair					
Sex with spouse	0.787	679	0.772	655	0.208
Sex with multiple partners	0.757	679	0.737	654	0.070

Notes: The table shows the balance between treatment and control group for the transmission risks variables measured in 2010. p-value shows the p-value of a t-test where the null hypothesis is that the difference in means between treatment and control group is zero. Panel A shows results for all respondents while panel B shows results excluding individuals living in the second biggest village pair which causes most of the imbalance.

Table C.11: Robustness tests for imbalance in perceived transmission risk

	Risky sex	Population survival		Own survival	HIV expectations	
		healthy	HIV	5 years	$p^1 - p^0$	HIV prob
Drop pair	(1)	(2)	(3)	(4)	(5)	(6)
BenKnow treatment	-0.164***	0.037***	0.047***	-0.001	0.030**	-0.038***
	(0.011)	(0.046)	(0.014)	(0.015)	(0.013)	(0.014)
Observations	1377	1320	1318	1283	1316	1354
Entropy weights	(1)	(2)	(3)	(4)	(5)	(6)
BenKnow treatment	-0.149**	0.039***	0.042***	-0.004	0.020	-0.044***
	(0.058)	(0.011)	(0.014)	(0.013)	(0.018)	(0.014)
Observations	1433	1379	1377	1339	1418	1409

Notes: The top panel shows regression coefficients for the BenKnow treatment effect on selected outcomes excluding individuals living in the second biggest village pair which causes most of the imbalance. The bottom panel shows regression coefficients for the BenKnow treatment effect on selected outcomes reweighting the sample using entropy weights to balance treatment and control group on transmission risk with having sex with multiple partners. Healthy and HIV refer to the updating in population survival probabilities. 5 years refers to the updating in own survival probabilities. Risky sex is a dummy variable taking value 0 if sexually passive, 1 if having sex with the spouse only, 2 if having multiple sexual partners and using condom during the last intercourse, 3 if having multiple sexual partners and not using condom during the last intercourse. $p^1 - p^0$ is the difference in transmission risk betweeen having sex with multiple partners and having sex with the spouse only. HIV prob is the update in the subjective probability of being HIV+ from baseline to the 2018 follow-up. All analyses additionally control for age group, years of schooling and randomization strata. Standard errors are clustered at the village level.

Table C.12: Instrumental variable estimates of the effect of transmission risk on sexual behaviour

	First stage	Second stage		
	Transmission risk multiple partners	Had sex	Sex and condom	Married
	(1)	(2)	(3)	(4)
BenKnow treatment	0.048***			
	(0.016)			
Transmission risk with multiple partners		-2.309***	-2.319***	2.490***
		(0.557)	(0.614)	(0.453)
Marginal effects	[0.048]	[-0.565]	[0.523]	[0.297]
			[-0.184]	
			[-0.021]	
			[-0.317]	
F-stat	7.1			
Observations	1418	1410	1420	1412

Notes: The table shows regression coefficients for the effect of transmission risk with multiple partners on sex related behaviours using BenKnow treatment as an instrument. The first column shows the first stage. Estimates from columns 2,3 and 4 are based on (ordered) probit specification where transmission risk is instrumented with BenKnow treatment. Sexual Risk Indices are defined as: Had Sex: 0 = not sexually active in the last 12 months, 1 = sexually active in the last 12 months; Sex and Condom: 0 = not sexually active in the last 12 months, 1 = sex with spouse only, 2 = sex with multiple partners and condom at last intercourse, 3 = sex with multiple partners and no condom at last intercourse; Married is an indicator equal to 1 if respondent is married in the follow-up. Marginal effects for Sex and Condom show one coefficient for each level of risky sexual behaviour. Analyses control for baseline levels. All analyses additionally control for age group, years of schooling and randomization strata. Standard errors are clustered at the village level. Standard errors in parentheses * $p < 0.1$, ** $p < 0.05$, *** $p < 0.01$

Table C.13: BenKnow treatment effects on subjective health and wellbeing

	(1) **Subjective Wellbeing**	(2) **SF12 Physical Score**	(3) **SF12 Mental Score**
BenKnow treatment	-0.032	-0.006	-0.005
	(0.055)	(0.031)	(0.049)
Observations	1,478	1,466	1,466

Notes: The table shows regression coefficients for the BenKnow treatment effect on subjective health and wellbeing. SF12 physical and mental score are constructed using a 12 item questionnaire on general health, physical activity and includes emotional health. Subjective wellbeing is based on the question "How satisfied are you with your life, all things considered?," with responses ranging from 1 = very unsatisfied to 6 = very satisfied. Analyses control for baseline levels. All analyses additionally control for age group, years of schooling and randomization strata. Standard errors are clustered at the village level.
