# Supplementary material for: MORTALITY RISK INFORMATION, SURVIVAL EXPECTATIONS AND SEXUAL BEHAVIOURS
Source: Econ J (London). Author manuscript; Available in PMC 2025 May 1. (PMC11065140; doi:10.1093/ej/uead116)
Supplement: Zipped Data - Replication File [file NIHMS1979496-supplement-Zipped_Data_-_Replication_File.zip › 3-replication-package/2017-06-comrec-benefits-of-knowledge-approval.pdf]

UNIVERSITY OF MALAWI

CERTIFICATE OF ETHICS APPROVAL

This is to certify that the College of Medicine Research and Ethics Committee (COMREC) has reviewed and approved a study entitled:

P.04/17/2160 - The benefits of knowledge: Mortality risks, Mental Health and Life – Cycle Behaviors version 1 dated 2 April 2017 by Dr. H. Kohler

On

As you proceed with the implementation of your study, we would like you to adhere to international ethical guidelines, national guidelines and all requirements by COMREC as indicated on the next page

14-Jun-17

Dr. YB. Mlombe - Chairperson (COMREC)

Date
