# Supplementary material for: MORTALITY RISK INFORMATION, SURVIVAL EXPECTATIONS AND SEXUAL BEHAVIOURS
Source: Econ J (London). Author manuscript; Available in PMC 2025 May 1. (PMC11065140; doi:10.1093/ej/uead116)
Supplement: Zipped Data - Replication File [file NIHMS1979496-supplement-Zipped_Data_-_Replication_File.zip › 3-replication-package/Benknowprocedure.pdf]

Experimental Procedure for
Mortality Risk Information, Survival Expectations and Sexual Behaviours

by Alberto Ciancio, Adeline Delavande, Hans-Peter Kohler and Iliana Kohler.

September 2023

Selection and eligibility of criteria of participants

The Malawi Longitudinal Study of Families and Health (MLSFH) is an ongoing longitudinal panel study established in 1998. Participants for this study come from the MLSFH Mature Adult Cohort (MLSFH-MAC), which was established by selecting in 2012 MLSFH respondents aged 45+ years, and enrolling them as part of an extensive aging and health baseline survey with follow-up waves in 2013, 2017, and 2018. The key inclusion criteria in 2012 for enrollment in the MLSFH-MAC were twofold: (i) being a MLSFH respondent aged 45 years or older in 2012; and (ii) having been interviewed in both the 2008 and 2010 MLSFH data collection rounds.
Baseline enrollment in the MLSFH-MAC included 1,266 individuals clustered in 130+ villages, representing more than 90% of the 1,402 eligible MLSFH respondents who met the enrollment criteria (= target sample). Migration out of the study areas and mortality were the primary reasons for not enrolling eligible respondents. At each follow-up, the study population was augmented with additional MLSFH respondents who newly reached eligibility. To ensure an adequate representation of HIV+ individuals in the cohort, age-eligible HIV+ respondents were enrolled if they participated in either the 2008 or 2010 MLSFH data collection. Through the ongoing enrollment and migration follow-ups, the MLSFH-MAC cohort expanded to 1,257 respondents in 2013, 1,606 in 2017, and 1,532 in 2018.

The respondents from the 2017 MLSFH-MAC wave were enrolled in our experimental study, and were followed one year later in 2018.

Random assignment to treatment and control groups

The Benefits of Knowledge (BenKnow) intervention randomly assigned 2017 MLSFH-MAC respondents to a treatment and a control group, with randomisation occurring at the village level to avoid spill-over effects between groups. Within each of the three study regions, villages were paired by size starting from the two biggest villages, followed by the two second biggest, etc. Then we randomly assigned treatment status to one village in each pair. The procedure guaranteed a similar sample size in the treatment group (N=779 in 58 villages) and control group (N=774 in 57 villages). The response rate for the BenKnow intervention was more than 98% (among 2017 survey respondents), resulting in 770 respondents enrolled in the treatment group.

Implementation of the health-information intervention

The Benefits of Knowledge (BenKnow) health-information intervention was implemented face-to-face and one-on-one by a separate survey team within two weeks subsequent to the 2017 MLSFH-MAC Main Survey.

Health-information intervention content

The intervention consisted of the following two core components:

(i) Narratives about changing mortality provided by video clips

Respondents were initially shown three video clips with a duration of about four minutes each. In these short video clips, individuals (trained local actors following a prepared script) explained how they noticed that people nowadays live longer in rural Malawi. The first video depicts a carpenter in his workshop, the second a female tailor in her shop sitting at a sewing machine and the third an old man sitting in front of his house. The videos emphasize overall that people live longer due to better access to food, health care, and availability of ART. The script of each video clip is provided in the intervention protocol.

(ii) Life-table survival probabilities conveyed via visual aids

Subsequent to the videos, respondents were shown a health-information sheet with visual information on 5-year and 10-year life-table survival probabilities for individuals of the same gender and within the same 5 year age group, with different figures conveying how many persons, out of 10 alive at the time of the intervention, could be expected to be alive five or ten years in the future. The Life table survival probabilities were obtained from the Global Burden of Disease Collaborative Network. Global Burden of Disease Study 2016 (GBD 2016) Results. Seattle, United States: Institute for Health Metrics and Evaluation (IHME), 2017. Available from http://ghdx.healthdata.org/gbd-results-tool. A BenKnow health-information sheet is illustrated Figure 1, and the complete set of probabilities for all gender and age group is reported in Table 1.

	Probability of dying			
	Men		Women	
Age	within 5 years	within 10 years	within 5 years	within 10 years
< 45	0.06	0.13	0.04	0.08
45-49	0.07	0.15	0.05	0.1
50-54	0.08	0.18	0.06	0.13
55-59	0.1	0.23	0.07	0.17
60-64	0.14	0.31	0.11	0.25
65-69	0.2	0.43	0.16	0.37
70-74	0.28	0.58	0.24	0.53
75-79	0.41	0.71	0.38	0.68
80+	0.51	0.76	0.49	0.74

The table reports mortality probabilities for each demographic group that were conveyed during the Benefits-of-Knowledge Health-information Intervention using information sheets like the one shown in Figure B.1. Lifetable survival probabilities were obtained Global Burden of Disease Collaborative Network. Global Burden of Disease Study 2016 (GBD 2016) Results. Seattle, United States: Institute for Health Metrics and Evaluation (IHME), 2017. Available from http://ghdx.healthdata.org/gbd-results-tool

Table 1: Life table probabilities of dying for BenKnow health-information intervention

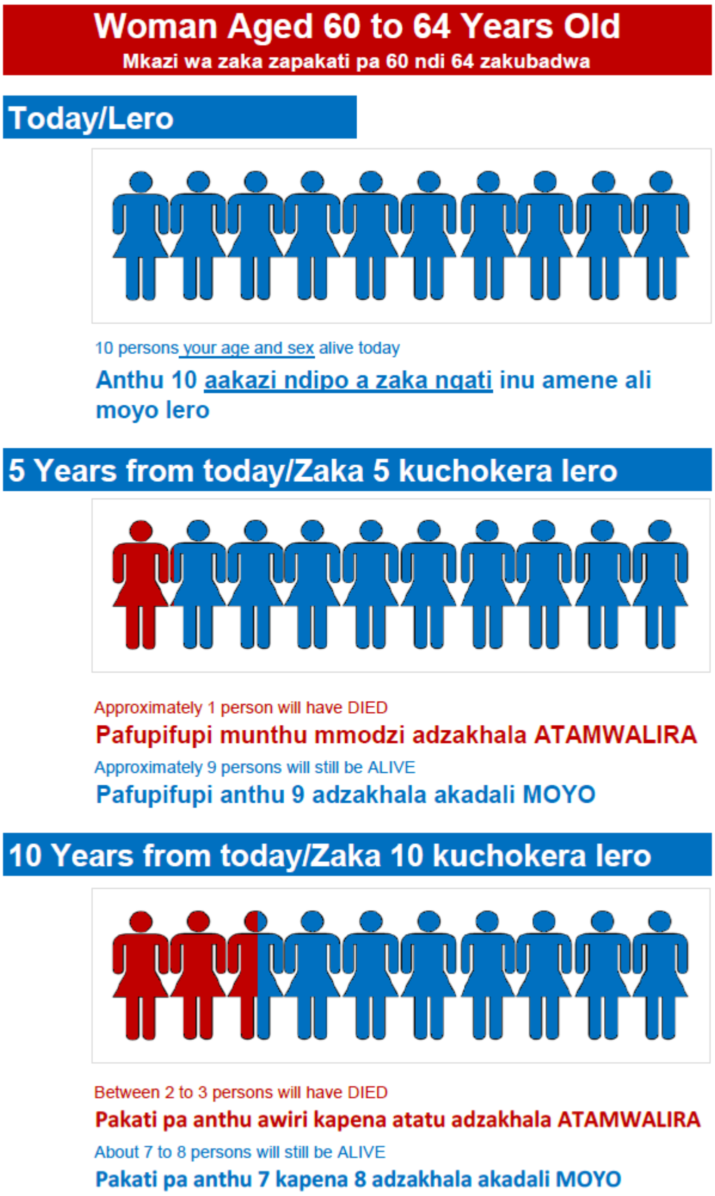

Figure 1: Health information sheet providing life-table-based information about 5-year and 10-year mortality probabilities for a woman aged 60-64 years old.
