# Supplementary material for: MORTALITY RISK INFORMATION, SURVIVAL EXPECTATIONS AND SEXUAL BEHAVIOURS
Source: Econ J (London). Author manuscript; Available in PMC 2025 May 1. (PMC11065140; doi:10.1093/ej/uead116)
Supplement: Zipped Data - Replication File [file NIHMS1979496-supplement-Zipped_Data_-_Replication_File.zip › 3-replication-package/intervention-protocol.pdf]

The Benefits of Knowledge: Mortality risk, Mental health and Life-cycle behavior

Protocol and Questionnaire for Health Information Intervention

Section 1---Background Information Pre-Intervention

Pamene a kafukufuku anabwera tsiku lina, anafunsa mafunso okhudza chiyembekezo cha mmene anthu ena kapena inu mungayembekezere kumwalira pamene nthawi ikudutsa pogwiritsa ntchito mtedza khumi (10).
When the survey team came to your house the other day, they asked you some questions about the chances that some people or you might die as time goes by using 10 peanuts.

**BK0**	**Kodi mukukumbukila mafunso amene aja?**<br>*Do you remember those questions?*	**Inde** Yes……………….1<br>**Ayi** No…………………2

Tiyeni tione mayankho anu limodzi
Let's look at your answers together.

INTERVIEWER: Verify the number of peanuts that respondent put when previously interviewed. Put the corresponding number of peanuts in the cup for 5 years probabilities and the corresponding number of peanuts in the cup for 10 years probabilities. Show the respondent the cup with [M9_X7A] peanuts for the 5 years probabilities and the cup with [M9_X7B] peanuts for the 10 years probabilities. Do not remove the peanuts from the cups and keep them in front of the respondent during the whole time of the interview!

Munayika mtedza [___], kutanthauza kuti pali kuthekera kokwana [__] pa maulendo khumi (10) aliwonse, pamene munafunsidwa za kuthekera koti mukhoza kumwalira mu zaka zisanu (5) zikubwerazi. [Interviewer lay out M9_X7A peanuts for 5-year mortality risk on flat surface]
*You allocated [M9_X7A] peanuts, meaning [M9_X7A] chances out of 10, when asked about the chances that you might die in the next **5 years**. [Interviewer: lay out M9_X7A peanuts for 5-year mortality risk on flat surface]*

Munayika mtedza [___], kutanthauza kuti pali kuthekera kokwana [__] pa maulendo khumi (10) aliwonse, pamene munafunsidwa za kuthekera koti mukhoza kumwalira mu zaka khumi (10) zikubwerazi. [Interviewer lay out M9_X7B peanuts for 10-year mortality risk on flat surface, below the M9_X7A peanuts]
*You allocated [M9_X7B] peanuts, meaning [M9_X7B] chances out of 10, when asked about the chances that you might die in the next **10 years**. [Interviewer lay out M9_X7B peanuts for 10-year mortality risk on flat surface, below the M9_X7A peanuts]*

BK1	**Kodi mwaonako kuti anthu okhala kumudzi ku Malawi kuno akukhala moyo nthawi yaitali kuyelekeza ndi mmene zinalili zaka zisanu kapena khumi zapitazo?**<br>*Have you noticed lately that people in Malawi living in villages like yours tend to live longer than they used to 5 or 10 years ago?*	**Inde** Yes.................................1<br>**Ayi** No .... 2→ continue with videos following the exact sequence below; start with Video 1 (Story 1)
BK2	**Kodi munazindikira bwanji kuti anthu akukhalitsa kusiyana ndi mmene zinalili zaka zisanu kapena khumi zapitazo?**<br>*How did you notice that people tend to live longer than they used to 5 or 10 years ago?*<br>***[check all answers that apply]***<br>*Interviewer: probe if the respondent does not provide*	**Ndimapita ku maliro owelengeka** I go to fewer funerals...1<br>**Ndinaona kuti anzanga ndi abale anga ochepa ndi amene akumwalira** I noticed that fewer of my friends and relatives are dying ....2<br>**Ndaona kuti anthu akumwalira atakalamba** I notice that

initially a response.	people are dying when they are older ……………..………3<br>**Chithandizo cha Edzi chikupezeka pafupi** AIDS treatment has become available nearby............................4<br>**Ntchito zaumoyo zapita patsogolo, ndipo izi ndizothandiza anthu** Health services have improved, and this helps individuals...5<br>**Zina** Other [_________________________________]........6

Section 2---Videos

[CONTINUE WITH VIDEOS:]

Tsopano ndikufuna ndikuonetseni kanema amene akuonetsa kuti masiku ano anthu ku Malawi akukhalitsa ndi moyo kuyelekeza ndi zaka zisanu kapena khumi zapitazo. Kanemayu wajambulidwa ndi anthu a zisudzo ndipo nkhani zili mkatimu zikugwilizana ndi mmene za umoyo ndi imfa zikuyendera ku Malawi kuno.

*I would like to show you a video showing that people in Malawi are living longer nowadays than 5 or 10 years ago. These videos have been recorded by **actors** and the information in these videos is consistent with recent health and mortality trends in Malawi.*

Video 1 (Story 1---Davie the carpenter):

A middle-aged man, working it his carpenter's shop, talks: Hi, my name is Davie and I have a bit of land where I grow maize. I also know how to work with wood. I am lucky because both my parents are still alive. They are both in their 70ies and are doing well. They are taking care of themselves: they have enough food, they are in good health and they don't need to go often to the hospital and they actively participate in village activities. They also teach important things about life to me and my children. They knew that they could live longer than their parents and with the little they were earning they bought some livestock to support themselves in their old days. My brothers and I also help them sometimes. My aunties and uncle also died very old. They were more than 65. And I see a lot of other families in our village with old family members that are still alive. My grand-parents were not so lucky and they were dead when they were my age. Yes, I really notice that people are living longer nowadays. And it is a good thing for everyone.

A middle-aged man, working in his carpenter's shop, talks: Moni, dzina langa ndine Davie ndipo ndili ndi malo pang'ono omwe ndimalimapo chimanga komanso ndili ndi luso lopala matabwa. Ndili ndi mwayi chifukwa makolo anga onse adakali moyo. Onse ali mu zaka za mma 70 ndipo ali ndi moyo wabwino. Akuzisamalira okha, ali ndi chakudya chokwanira, umoyo wao ndi wabwino, sapita kuchipatala pafupipafupi, ndipo amatenga nao gawo kwambiri muzochitika za mmudzi muno. Amatiphunzitsanso makhalidwe abwino ine ndi ana anga. Ankadziwa kuti atha kukhala moyo wautali kuyelekeza ndi makolo awo ndipo ndi ndalama zochepa zomwe amapeza anagula ziweto zoti zidzawathandize akadzakalamba. Ine ndi azibale anga timawathandiza nthawi zina. Atsibweni ndi azakhali anga anamwaliranso atakalamba. Anali ndi zaka zoposera 65 zakubadwa. Komanso, ndimaona mabanja ambiri mmudzi mwathu muno amene ali ndi achibale okalamba omwe adakali moyo. Agogo anga sanachite mwayi okhala ndi moyo nthawi yayitali ndipo anamwalira ali ndi msinkhu ngati wangawu. Inde, ndikutha kuona kuti anthu masiku ano akukhalitsa ndi moyo wautali. Ndipo ichi ndi chinthu chabwino kwa wina aliyense.

Interviewer: continue with Video 2 --Rose

Video 2 (Story 2 -- Rose):

A middle-aged woman, working in her tailoring shop , talks: Hi, my name is Rose. I work in the field to plant cassava. When I have time, I do a bit of tailoring. I am married and I have four children who also help me in the field. The younger two go to school if they do not help at home. Five years ago, my husband got tested for HIV and he found out that he was HIV-positive. This was really a shock, and I was worried about the future of the family. How could we manage if my husband died soon? However, we have been lucky because my husband has had access to antiretroviral treatment (ART) in the local clinic. He takes his medicine regularly as the doctor explained him and I make sure he does not forget. He also often goes to the clinic for refill and check-ups. He looks really healthy and fit and does not show any sign of the disease. We do not know what will happen but we are very grateful for the availability of treatment. Ten years ago, my brother had HIV and he became very sick very quickly and died rapidly. Nowadays, there is more hope for people with HIV thanks to the availability of treatment. They can expect a longer life.

A middle-aged woman, working in her tailoring shop , talks: Moni. Dzina langa ndiine Rose. Ndimagwira ntchito ya ulimi ndipo ndimalima chinangwa. Ndikakhala ndi nthawi, ndimasoka zovala. Ndili pa banja ndipo ndili ndi ana anayi amene amandithandizanso kulima. Ana awiri aang'ono amapita ku sukulu pa nthawi imene samandithandiza pakhomo. Zaka zisanu zapitazo, amuna anga anakayezetsa HIV ndipo anapezeka kuti ali ndi kachilombo ka HIV. Ichi chinatizizimutsa kwambiri ndipo ndinali ndi nkhawa ndi tsogolo la banja lathu. Kodi tidzakwanitsa bwanji ngati amuna anga angamwalire posachedwa? Komabe, takhala ndi mwayi chifukwa amuna anga anapeza mwayi olandira mankhwala otalikitsa moyo a ARV kuchokera ku chipatala cha mdera lino. Iwowa amamwa mankhwala pafupipafupi molingana ndi mmene a dokotala amawalangizila ndipo ndimaonetsetsa kuti asaiwale. Amapitanso ku chipatala kukatenga mankwala ena komanso kuti akawaunike m'thupi. Iwowa amaoneka a thanzi ndi mphamvu ndipo samaonetsa zizindikilo zina zilizonse za matendawa. Sitikudziwa kuti kudzachitika chiani kutsogoloku koma tili othokoza chifukwa cha kupezeka kwa mankhwala otalikitsa moyo. Zaka khumi zapitazo, mchimwene wanga anali ndi kachilombo ka HIV ndipo anayamba kudwala mofulumira mpaka anamwalira mosachedwetsa. Masiku ano pali chiyembekezo kwa anthu amene ali ndi kachilombo ka HIV chifukwa cha kupezeka kwa mankhwala otalikitsa moyo. Anthuwa akhoza kukhala moyo wautali.

Interviewer: continue with Video 3 – the old man

Video 3 (Story 3 – old man):

An old man seating at home: I am lucky because I am more than 60 years old and I am still alive and feel healthy. I am not the only luck one. My neighbor next door is more than 70. And think about the popular musician Giddes Chalamanda. He is over 85 years old, and is still performing for the people. Last year, he even made is long-held dream of going to America come true, giving several shows across the USA. My parents were not so lucky because they died when they were in their 40ies. I think things are better nowadays. The kids, they do not die so frequently anymore. They get their immunization and many sleep under bed nets. They do not get sick so often. The adults, they do not die from HIV so rapidly anymore. The treatments, they really help. Also, people are not so hungry anymore and they eat more. When I was a kid, we were often hungry. My children and grand-children, they have almost always their meal on the table. It helps to build your health and keep you strong and prevent you from being unwell. Yes, things have changed quite a lot and people are less sick and live longer.

An old man seating at home: Ndiine odala chifukwa ndili ndi zaka zopyola 60 ndipo ndikadali moyo komanso ndimdziona wa thanzi. Sindili ndekha odala. Anzanga oyandikana nawo ali ndi zaka zopyola 70. Ndipo taganizilani za oyimba uja Giddes Chalamanda; ali ndi zaka zopyola 85 koma akadaimbilabe anthu. Chaka chatha, anakwanilitsa maloto ake a nthawi yaitali popita ku America, ndipo anakaimba madera osiyanasiyana ku America. Makolo anga analibe mwayi umenewu chifukwa anamwalira ali ndi zaka za mma 40'chakuti. Ndikuona kuti zinthu zili bwino masiku ano. Ana anasiye kumwalira pafupipafupi masiku ano. Amalandira katemera ndipo amagona m'masikito. Sadwaladwala kawirikawiri. Pamene akuluakulu samwalira mwamsanga akakhala ndi kachilombo ka HIV masiku ano. Mankhwala otalikitsa moyo akuthandiza kwambiri. Komanso, anthu sakukhala a njala ndipo akumadya muchulukirapo. Pamene ndinali wang'ono, timakhala a njala kawirikawiri. Ana ndi zidzukulu zanga amalandira chokudya pafupifupi nthawi zones. Izi zimathandiza kuti thupi likhale la thanzi ndi la mphamvu komanse kuteteza kuti usadwale. Inde, zinthu zasintha kwambiri ndipo anthu sadwaladwala komanso akukhala moyo wautali.

END OF VIDEO

Section 3--- Provision of Updated Mortality Information

[INTERVIEWER: SELECT THE MORTALITY INFORMATION SHEET CORRESPONDING TO THE RESPONDENT'S AGE AND SEX. USE THE INFORMATION ON THIS SHEET WHEN WE REFER TO 'MORTALITY INFO SHEET' BELOW]

Gulu lathu la kafukufuku linayang'ana zotsatila za kafukufuku zoonetsa mmene anthu ku Malawi akumwalilira komanso mmene anthu aakazi/aamuna a zaka ngati inu akuyembekezekera kukhala moyo. Kuchokera ku zotsatira za kafukuzi, ndi zotheka kuyerekeza mmene anthu aakazi/aamuna a zaka ngati inu angayembekezere kumwalira mu zaka zisanu kapena khumi zikubwerazi
Our research team has looked at some recent data showing how many individuals in Malawi are dying, and how long individuals your age and sex are likely to live. From these findings, it is possible to estimate how likely a person of your age and sex will die within five or ten years.

Tikufuna tikuonetseni zimenezi poonetsa zithunzi. Pa zithunzizi, anthu a blue ndi chizindikilo cha anthu amene ali moyo pamene anthu ofiila ndi chizindikilo cha anthu amene amwalira.
We would like to illustrate this to you with some pictures. In these pictures, blue persons indicate people who are alive, and red persons indicate people who have died.

Tiyamba ndi anthu khumi aakazi/aamuna a zaka ngati inu. Anthu khumi amenewa ali moyo pakadali pano ndipo akukhala m'Malawi muno mu dera ngati lanuli. Mukuona anthu khumi mu chithunzichi amene onse ali a blue, kapena kuti amoyo [INTERVIEWER: SHOW FIRST GRAPH ON THE MORTALITY INFO SHEET].
*We begin with 10 hypothetical persons who are about your age and are of the same sex. These 10 persons are alive today, and they live in Malawi in a similar context as you do. You can see these 10 persons in this figure that shows 10 blue, or alive, persons [INTERVIEWER: SHOW **FIRST GRAPH** ON THE MORTALITY INFO SHEET].*

Tsopano tiyeni tione zaka zisanu zikubwerazi ndipo tiunike kuti ndi anthu angati mwa anthu amene ali pa chithunzipa adzakhale ndi moyo zaka zisanu kuchokera lero. Mmene mukuonera pa chithunzipa [SHOW SECOND GRAPH "5 YEARS FROM TODAY" ON THE MORTALITY INFO SHEET], ena mwa anthuwa adzakhala atamwalira, ndipo akuoneka ofiila, pamene ena adzakhala ali moyo, ndipo akuoneka a blue, zaka zisanu kuchokera lero. Chiwerengero cha anthu ofiila mu chithunzichi chikuonetsa kuti nkotheka bwanji kuti munthu wa mkazi/mwamuna wa zaka ngati inu atha kumwalira mu zaka zisanu zikubwerazi; pamene chiwerengero cha anthu ofiila chikukwera, zikuonetsa kuti kuthekera ndi kochuluka kuti munthu akhoza kumwalira.
*We can now look five years into the future, and ask how many of the persons in the first figure will still be alive **5 years from today**. As you see on this picture [SHOW SECOND GRAPH "5 YEARS FROM TODAY" ON THE MORTALITY INFO SHEET], some of the persons will have died, and are shown in **red**, and others will still be alive, and are shown in **blue**, five years from today. How many persons are in red in this graph tells the chance out of 10 that a person your age and sex will die within the next **five** years: the more people we show in red (or the more red a person is), the higher is the risk of dying.*

Kutengera pa zimene tikudziwa lero, tikuona kuti [READ RED LINE IN 5-YEARS FROM TODAY SECTION] mu zaka zisanu zikubwerazi kuchokera lero, pamene [READ BLUE LINE IN 5-YEARS FROM TODAY SECTION].
Based on our knowledge today, we predict that [READ RED LINE IN 5-YEARS FROM TODAY SECTION] within 5 years from today, while [READ BLUE LINE IN 5-YEARS FROM TODAY SECTION] within 5 years from today.

Tikhonzanso kuwunika zaka khumi zikubwerazo kuchokera lero ndikuonanso kuti ndi anthu angati mu chithunzi choyamba amene adzakhale akadali moyo zaka khumi kuchokera lero. Mmene mukuonera pa chithunzipa [SHOW THIRD GRAPH "10 YEARS FROM TODAY" ON THE MORTALITY INFO SHEET] ena mwa anthuwa adzakhala atamwalira, ndipo akuoneka ofiila, pamene ena adzakhala ali moyo, ndipo akuoneka a blue, zaka khumi kuchokera lero. Chiwerengero chan anthu ofiila mu chithunzichi chikuonetsa kuti nkotheka bwanji kuti munthu wa mkazi/mwamuna wa zaka ngati inu atha kumwalira mu zaka khumi zikubwerazi; pamene chiwerengero cha anthu ofiila chikukwera, zikuonetsa kuti kuthekera ndi kochuluka kuti munthu akhoza kumwalira.
*We can also look ten years into the future, starting today, and how many of the persons in the first figure will still be alive **10 years from today**. As you see on this picture [SHOW THIRD GRAPH "10 YEARS FROM TODAY" ON THE MORTALITY INFO SHEET], some of the*

*persons will have died, and are shown in **red**, and others will still be alive, and are shown in **blue**, ten years from today. How many persons are in red in this graph tells you is the chance out of 10 that a person your age and sex will die within the next **ten** years. The more people we show in red (or the more red a person is), the higher is the risk of dying.*

Kutengera pa zimene tikudziwa lero, tikuona kuti [READ RED LINE IN "10-YEARS FROM TODAY" SECTION] mu zaka khumi zikubwerazi kuchokera lero, pamene [READ BLUE LINE IN "10-YEARS FROM TODAY" SECTION] mu zaka khumi zikubwerazi kuchokera lero
Based on our knowledge today, we predict that [READ RED LINE IN "10-YEARS FROM TODAY" SECTION] within 10 years from today, while [READ BLUE LINE IN "10-YEARS FROM TODAY" SECTION] within 10 years from today.

Inde, palibe amene angalosere zimene zingamuchitikire munthu wina, koma zotsatira za kafukufuku wathu zikhoza kukuonetserani kuti nkothekwa bwanji kuti chinthu chikhoza kuchitika powunika gulu lalikulu la anthu aakazi/aamuna ofanana nao zaka. Ndipo zotsatirazi ndi zothandiza kwa inu poona kuti nkotheka bwanji kuti mukhoza kumwalira mu zaka zisanu kapena khumi zikubwerazi. Izi ndi zimene takhala tikuchita. Choncho, tikawunika anthu khumi aakazi/aamuna a zaka ngati inu, tikuona kuti:
Of course, nobody can predict what will happen to a specific individual, but this information can tell you about what is likely to happen if we look at a large group of people of your age and sex. And this information is helpful for you to think how likely you might die within the next 5 or 10 years. So, let's summarize this information: if we look at 10 persons your age and sex:

- **[READ RED LINE IN "5-YEARS FROM TODAY" SECTION] mu zaka zisanu zikubwerazi kuchokera lero, pamene [READ BLUE LINE IN "5-YEARS FROM TODAY" SECTION] mu zaka zisanu zikubwerazo kuchokera lero, ndipo**
- *[READ RED LINE IN "5-YEARS FROM TODAY" SECTION] within 5 years from today, while [READ BLUE LINE IN "5-YEARS FROM TODAY" SECTION] within 5 years from today; and*
- **[READ RED LINE IN "10-YEARS FROM TODAY" SECTION] mu zaka khumi zikubwerazo kuchokera lero, pamene [READ BLUE LINE IN "10-YEARS FROM TODAY" SECTION] mu zaka khumi zikubwerazo kuchokera lero**
- *[READ RED LINE IN "10-YEARS FROM TODAY" SECTION] within 10 years from today, while [READ BLUE LINE IN "10-YEARS FROM TODAY" SECTION] within 10 years from today*

Choncho potsatira zomwe ndakuuzanizi, nditati nditenge mtedza omwe uyimire kuti nkotheka bwanji kuti munthu wa mkazi/mwamuna wa zaka ngati inu angamwalire mu zaka zisanu, ndingaike mtedza [INTERVIEWER: PICK THE NUMBER OF BEANS THAT CORRESPONDS TO THE NUMBER OF RED PEOPLE ON THE FIGURE WITH 5-YEARS MORTALITY INFO] m'balemu.
So based on this information, if I were to pick the number of peanuts that reflects how likely it is that a person your age and sex would die within 5 years, I would put **[INTERVIEWER: PICK THE NUMBER OF BEANS THAT CORRESPONDS TO THE NUMBER OF RED PEOPLE ON THE FIGURE WITH "5-YEARS FROM TODAY" MORTALITY INFO]** *peanuts on the plate.*
Interviewer: Put the number of beans infront of the cup with the 5-years chances of dying. Do not remove the peanuts but leave on the ground. So the respondent can see original answer in the cup, and new information on the ground until the end of the interview.

Choncho potsatira zomwe ndakuuzanizi, nditati nditenge mtedza omwe uyimire kuti nkotheka bwanji kuti munthu wa mkazi/mwamuna wa zaka ngati inu angamwalire mu zaka khumi, ndingaike mtedza [INTERVIEWER: PICK THE NUMBER OF BEANS THAT CORRESPONDS TO THE NUMBER OF RED PEOPLE ON THE FIGURE WITH 10-YEARS MORTALITY INFO] m'balemu.
So based on this information, if I were to pick the number of peanuts that reflects how likely it is that a person your age and sex would die within 10 years, I would put **[INTERVIEWER: PICK THE NUMBER OF BEANS THAT CORRESPONDS TO THE NUMBER OF RED PEOPLE ON THE FIGURE WITH "10-YEARS FROM TODAY" MORTALITY INFO]** *peanuts on the plate.*

Interviewer: Put the number of beans infront of the cup with the 5-years chances of dying. Do not remove the peanuts but leave on the ground. So the respondent can see original answer in the cup, and new information on the ground until the end of the interview.

***[Interviewer:** The following is an example how to use ½ peanuts and whole peanuts if the figures are partially colored in red. 1) If the instructions on the mortality info sheet say "less than 1 person will have died" put ½ a peanut; 2) If the instructions on the mortality info sheet say "Between 2 and 3 persons will have died" or "About 2 and 3 persons will have died" then put 2½ peanut. In all other cases put a whole peanut (for example, if instructions say "almost [#] persons will have died", "about 1 person will have died", "approximately[#] persons will have died", "almost [#] persons will have died", "slightly more [#] persons will have died".*

**BK3:** **Kodi mukumvetsetsa nkhaniyi?**<br>*Do you understand this information?*	**Inde** Yes ....1 → SKIP BK3a<br>**Ayi** No.............2 → go back to beginning of Section 3 above, and explain again to respondent and ask BK3a;
**BK3a. Kodi mukumvetsetsa nkhaniyi?**<br>*Do you understand this information?*	**Inde** Yes ....1<br>**Ayi** No........................2
**BK3b. Kodi mukuganiza kuti mfundo zomwe zakambidwazi zikusonyeza molondola zomwe zimachitika kwa anthu a zaka ngati inu omwe akumamwalira mmudzi muno masiku ano?** Do you think this information reflects correctly what happens to people of your age and sex dying in your community nowadays?	**Inde, zikusonyeza molondola** Yes, reflects correctly...1<br>**Inde, zikusonyeza pang'ono** Yes, reflects somewhat...2<br>**Ayi, sizikusonyeza molondola** No, does not reflect correctly...3<br>**Sindikudziwa** Dont' Know...4

Zindikirani kuti kutengera mmene mulili umoyo wanu komanso mmene lilili banja lanu ndi kapezedwe kanu, zikhozakupangitsa kuti kukhale kotheka kapena kosatheka kuti mumwalire kuyerekeza ndi munthu wa mkazi/mwamuna amene ali mu gulu lalikulu la anthu
Of course, depending on your health and depending on your own family and economic context, you might be more or less likely to die than the average person your age and sex in a large group.

Tsopano, ndikufuna ndikufunseninso zokhudza kuthekera koti inu mukhoza kumwalira mu zaka zisanu kapena khumi zikubwerazi. Taonani mtedza omwe munaika poyamba paja kuimirira kuthekera koti inu mukhoza kumwalira ku zaka zisanu kapena khumi zikubwerazi. Kutengera zinthu zomwe ndakuuzani, komanso zomwe mukudziwa zokhudza umoyo wanu, banja lanu ndi kapezedwe ka pakhomo panu, chonde yankhaninso mafunso otsatirawa. Kumbukirani kuti mutha kuswa mtedza pakatikati ndipo mutha kuika theka la mtedza moonjezera mtedza watunthu ngati mukufuna kusankha nambala ya pakati pa mtedza uwiri watunthu.
Now, I would like to ask you again about what you think about the chances that you might die in the next five or ten years. Look at the peanuts that you had put earlier for the chances that you will die within 5 years and 10 years. Based on what I have told you, and based on what you know about your own health, family and economic context please answer again the following questions below. Remember that you can break a peanut in ½ and put ½ peanut in addition to the whole peanuts if you want to pick a value between two whole peanuts.

Interviewer: Provide respondent with the empty 3rd cup in front of him/her. Give respondent 10 peanuts. Remind respondent that he/she can put ½ bean if respondent wants to pick value between two whole peanuts (e.g., respondent thinks 1 and 1/2 peanuts (1.5) is the best answer). If respondent is not able to break the peanut in ½, help him/her with this. If respondent used ½ peanut, do not substitute with a whole peanut.

**Tengani mtedza womwe uyimire m'mene inu mukuganizira kuti**<br>*Pick the number of peanuts that reflects how likely you think it is that you*	**# OF PEANUTS in plate**
**BK_X7a: mumwalira m'zaka zisanu (5) zikubwerazi kuyambira lero**<br>*will die within a <u>five-year</u> period beginning today*<br><br>**(LEAVE PEANUTS ON PLATE)**	[_____]<br><br>**if 10 → ask BK_X8a, or BK_X8b, or BK_X8c, or BK_X8d and if the answer is yes and the respondent does not revise his/her answer then continue to BK4.**

**BK_X8a: If BK_X7a>M9_X7A: Mayankho anu akusonyeza kuti panopa mukuganiza kuti kuthekera kwanu koti mutha kumwalira muzaka zisanu zikubwerazi ndikwakukulu kusiyana ndi komwe munanena poyamba paja ndisanakuonetseni mauthengawa. kodi izi ndi zomwe zinali mmaganizo mwanu?** Your answers show that you now think that the chance of dying within the next 5 years are larger than what you said before I gave you the information. Is that what you had in mind?	Yes 1<br>No 2<br><br>If No, go to **BK_X7a2**<br>If Yes, go to **BK_X7b**
**BK_X8b: If BK_X7a<M9_X7A: Mayankho anu akusonyeza kuti panopa mukuganiza kuti kuthekera kwanu koti mutha kumwalira muzaka zisanu zikubwerazi ndikwakung'ono kusiyana ndi komwe munanena poyamba paja ndisanakuonetseni mauthengawa. kodi izi ndi zomwe zinali mmaganizo mwanu?** Your answers show that you now think that the chance of dying within the next 5 years are smaller than what you said before I gave you the information. Is that what you had in mind?	Yes<br>No<br>If No, go to **BK_X7a2**<br>If Yes, go to **BK_X7b**
**BK_X8c: If BK_X7a=M9_X7A: Mayankho anu akusonyeza kuti panopa mukuganiza kuti kuthekera kwanu koti mutha kumwalira muzaka zisanu zikubwerazi ndikofanana ndi komwe munanena poyamba paja ndisanakuonetseni mauthengawa. kodi izi ndi zomwe zinali mmaganizo mwanu?** Your answers show that you now think that the chance of dying within the next 5 years are equal to what you said before I gave you the information. Is that what you had in mind?	Yes<br>No<br>If No, go to **BK_X7a2**<br>If Yes, go to **BK_X7b**
**Tengani mtedza womwe uyimire m'mene inu mukuganizira kuti**<br>Pick the number of peanuts that reflects how likely you think it is that you	# OF PEANUTS in plate
**BK_X7a2: mumwalira m'zaka zisanu (5) zikubwerazi kuyambira lero**<br>will die within a five-year period beginning today<br><br>**(LEAVE PEANUTS ON PLATE)**	[_____]<br>if 10 go to BK4 or BK5 depending if they changed their answer compared to the initial number of peanuts in the main questionnaire

Add the number of peanuts that reflects how likely you think it is that you:<br><br>**BK_X7b. wonjezerani mtedza m'balemu womwe uyimirire m'mene inu mkuganizira kuti mumwalira m'zaka khumi(10) zikubwerazi kuyambira lero**<br>*will die within a ten-year period beginning today*<br><br>**(IT IS POSSIBLE TO ADD ZERO ADDITIONAL PEANUTS)**	[_____]
**BK_X8d: If BK_X7b>M9_X7B: Mayankho anu akusonyeza kuti panopa mukuganiza kuti kuthekera kwanu koti mutha kumwalira muzaka khumi zikubwerazi ndikwakukulu kusiyana ndi komwe munanena poyamba paja ndisanakuonetseni mauthengawa. kodi izi ndi zomwe zinali mmaganizo mwanu?**Your answers show that you now think that the chance of dying within the next 10 years are larger than what you said before I gave you the information. Is that what you had in mind?	Yes<br>No<br>If No, go to **BK_X7b2**<br>If Yes, go to BK4
**BK_X8e: If BK_X7b<M9_X7B: Mayankho anu akusonyeza kuti panopa mukuganiza kuti kuthekera kwanu koti mutha kumwalira muzaka khumi zikubwerazi ndikwakung'ono kusiyana ndi komwe munanena poyamba paja ndisanakuonetseni mauthengawa. kodi izi ndi zomwe zinali mmaganizo mwanu?**Your answers show that you now think that the chance of dying within the next 10 years are smaller than what you said before I gave you the information. Is that what you had in mind?	Yes<br>No<br>If No, go to **BK_X7b2**<br>If Yes, go to BK4
**BK_X8f: If BK_X7b=M9_X7B: Mayankho anu akusonyeza kuti panopa mukuganiza kuti kuthekera kwanu koti mutha kumwalira muzaka khumi zikubwerazi ndikofanana ndi komwe munanena poyamba paja ndisanakuonetseni mauthengawa. kodi izi ndi zomwe zinali mmaganizo mwanu?** Your answers show that you now think that the chance of dying within the next 10 years are equal to what you said before I gave you the information. Is that what you had in mind?	Yes<br>No<br>If No, go to **BK_X7b2**<br>If Yes, go to BK4

**Tengani mtedza womwe uyimire m'mene inu mukuganizira kuti**<br><br>*Pick the number of peanuts that reflects how likely you think it is that you*	**# OF PEANUTS in plate**
**BK_X7b2: mumwalira m'zaka zisanu (5) zikubwerazi kuyambira lero**<br>*will die within a ten-year period beginning today*<br><br>**(LEAVE PEANUTS ON PLATE)**	[_____]<br><br>go to BK4 or BK5 depending if they changed their answer compared to the initial number of peanuts in the main questionnaire

Interviewer: Confirm if the respondent has changes the number of beans on the plate compared to his/her initial answer. If the respondent did NOT change his/her answer, continue with question BK4. If the respondent did change his/her answer, continue with question BK5.

**BK4**	**Kodi ndi chifukwa chiyani simunafune kusintha yankho lanu?** Why did you not want change your answer: **(select all that apply)**	**Ndimadziwa kale kuti anthu akukhala ndi moyo kwa nthawi yayitali, choncho sindinaphunzirepo china chilichonse chatsopano** I already knew that people live longer so I did not learn anything new …………1<br>**Sindikukhulupirira za nkhani zomwe mwandiuzazi** I do not believe the information you gave me 2

	**Nkhani zomwe mwandiuzazi sizinamveke kwenikweni** The information you provided was not very clear .... 3<br>**Palibe amene angalosere za imfa yake** Nobody can predict their mortality .... 4<br>**Zina** Other [_________________________________]...................... 5
**BK5. Kodi ndi chifukwa chiyani mwasintha yankho lanu?** Why did you change your answer? **(select all that appy)**	**Sindimadziwa kuti anthu akukhalitsa ndi moyo** I did not know that people live longer ……………………………………………………… 1<br>**Ndikukhulupirira za nkhani zomwe mwandiuza** I believe the information you gave me …………………………………………………2<br>**Nkhani zomwe mwandiuza ndizogwira mtima kwambiri** The information you provided to me was very convincing ……………………3<br>**Zina** Other …………………………………………………………………..4

Pomaliza, ndikufuna muganizire kuti nkotheka bwanji kuti munthu wina amwalire pamene nthawi ikudutsa. Ndikufunsani zokhudza munthu ongopeka yemwe akukhala mdera lanu, ndipo ndimulongosola munthuyu kwa inu.

Finally, I would like you to consider the likelihood that somebody else dies as time goes by. I am going to ask you about an imaginary person living in the same context like you, and I am going to describe him/her to you.

INTERVIEWER: Empty the 3rd cup infront of the respondent. For each of questions X8a to X8d start with an empty plate and 10 peanuts. Do not leave peanuts on plate. If the respondent used ½ peanut, replace it after asking the question with one whole peanut and make sure that the respondent starts with 10 whole peanuts.

**Tengani mtedza umene uyimire mmene mukuganizira kuti nkotheka bwanji kuti mmodzi mwa anthu awa akhoza kumwalira mu zaka zisanu kuchokera lero:**<br>*Pick the number of peanuts that reflects how likely you think it is that one of the following persons will die within a five-year period beginning today:*	**# of peanuts in plate**
**BK_X8a**<br>**For men:**<br>**Mwamuna wa zaka ngati inu wa thanzi ndipo alibe kachilombo ka HIV?**<br>*A man your age who is healthy and does not have HIV?*<br><br>**For women:**<br>**Mkazi wa zaka ngati inu wa thanzi ndipo alibe kachilombo ka HIV?**<br>*A woman your age who is healthy and does not have HIV?*	[_____]
**BK_X8b**<br>**For men:**<br>**Mwamuna wa zaka ngati inu amene ali ndi kachilombo ka HIV koma sanayambe kudwala?**<br>*A man your age who is infected with HIV?*<br><br>**For women:**<br>**Mkazi wa zaka ngati inu amene ali ndi kachilombo ka HIV koma sanayambe kudwala?**<br>*A woman your age who is infected with HIV?*	[_____]
**BK_X8c**<br>**For men:**<br>**Mwamuna wa zaka ngati inu amene ali ndi kachilombo ka HIV ndipo akudwala Edzi?**<br>*A man your age who sick with AIDS?*<br><br>**For women:**<br>**Mkazi wa zaka ngati inu amene ali ndi kachilombo ka HIV ndipo akudwala Edzi?**<br>*A woman your age who sick with AIDS?*	[_____]

BK_X8d<br>For men:<br>**Mwamuna wa zaka ngati inu amene akudwala Edzi ndipo akulandira mankhwala otalikitsa moyo a ARV?**<br>*A man your age who sick with AIDS and who is treated with antiretroviral treatments (ART)?*<br><br>For women:<br>**Mkazi wa zaka ngati inu amene akudwala Edzi ndipo akulandira mankhwala otalikitsa moyo a ARV?**<br>*A woman your age who sick with AIDS and who is treated with antiretroviral treatments (ART)?*	[_____]
