# Supplementary material for: MORTALITY RISK INFORMATION, SURVIVAL EXPECTATIONS AND SEXUAL BEHAVIOURS
Source: Econ J (London). Author manuscript; Available in PMC 2025 May 1. (PMC11065140; doi:10.1093/ej/uead116)
Supplement: Zipped Data - Replication File [file NIHMS1979496-supplement-Zipped_Data_-_Replication_File.zip › 3-replication-package/ReadMe.pdf]

Replication package for
Mortality Risk Information, Survival Expectations and Sexual Behaviours
by Alberto Ciancio, Adeline Delavande, Hans-Peter Kohler and Iliana Kohler.
September 2023

Overview

This file describes the instructions to replicate the results in the paper "**Mortality Risk Information, Survival Expectations and Sexual Behaviours**" by Alberto Ciancio, Adeline Delavande, Hans-Peter Kohler and Iliana Kohler.

Data Availability Statement

The data for the main analysis come from the Malawi Longitudinal Study of Family and Health https://web.sas.upenn.edu/malawiresearch/. We provide all the raw data needed for the analysis in this paper.

The data of the Malawi Longitudinal Study of Families and Health (MLSFH), from which the analyses data for this paper is created, is available according to the data sharing policy outlined in the MLSFH Cohort Profile (Kohler et al. 2015). This cohort profile is the main documentation for the general study design and sampling framework, and it summarizes some key findings as well. Until these MLSFH data have been released publicly, which is anticipated in the near future, the complete MLSFH data can requested by emailing Professor Hans-Peter Kohler hpkohler@pop.upenn.edu.

Only one appendix figure (Figure C.2) is created using data from the Demographic and Health Survey (DHS): https://dhsprogram.com/data/. DHS does not allow for redistribution, but access is free to any academic researcher. After registering for dataset access, click on "Download datasets" and select country "Malawi". Please download waves: 2015-16, 2010 and 2004 as well as the HIV biomarkers datasets for the same years.

We certify that the authors of the manuscript have legitimate access to and permission to use the data used in this manuscript.

Dataset list

- **Raw data from the MLSFH**

rawdata.csv -> includes MLSFH raw data from the 2017 and 2018 waves that are used for the main analyses.

rawdata_2019.csv -> includes data for the 2019 MLSFH wave for the analyses on pregnancies (table 4).

rawdata_survival.csv -> includes data on vital status for MLSFH respondents to reproduce Figure C.4 which shows the predictive power of subjective survival probabilities.

rawdata_sexual_behavior.csv -> includes data on sexual behavior for multiple MLSFH cohorts for appendix Figure C.1.

rawdata_survival_pooled.csv -> Survival expectations data for MLSFH respondents over the years to produce figure 1B.

- Raw data from the Global Burden of Disease Collaborative Network (2016)

southerneasternafrica-lifetables.cvs -> Life table data for Malawi from the Institute for Health Metrics and Evaluation (IHME) to produce figure 1.
These data can also be downloaded at
https://ghdx.healthdata.org/record/ihme-data/gbd-2019-life-tables-1950-2019.

Software Requirements
The software required to run all the code is Stata 16 or later. The only exception is Figure 1 that requires R.

Stata packages to be installed: combomarginsplot cmp ghk2 dataout ebalance estout reghdfe ftools distinct coefplot.

R packages to be installed: foreign.

Instructions for Data Preparation and Analysis
To reproduce the results of all tables and all figures in the paper (except for figure 1), the user needs to run the dofile “master” in the code folder. The user needs to change the working directory at the beginning of the dofile before running the code. Note that figures 2, 3, 4 and table 2 are conceptual maps and lists that do not need replication. The code takes around 25 minutes to run on a Macbook pro with processor 2.3 GHz Intel Core i5.

To reproduce figure 1 only, the user needs to change the working directory at the beginning of the two R scripts and run the code. The R code takes only a few seconds to run. Below, we briefly describe all the dofiles and R files in the code folder.

Description of programs/code

master -> Creates all tables and figures by calling all other dofiles

benknow_prep -> Create the main dataset for the analysis (mostly combining 2017-2018 rounds of the MLSFH survey)

analysis_main -> Creates all the tables in the main text using the main dataset and a few tables in appendix

bootstrap_ci -> Creates confidence intervals for Table 3 Panel B

benknow_sane -> Reproduces Table 4 using data from the 2019 MLSFH survey

descriptive -> Additional descriptive analyses: summary statistics, balance, imbalance for hiv

transmission risk, attrition and various statistics reported in the main text

prevalence -> Reproduces Figure C.2: HIV prevalence over time by age group using DHS data. Before running the code, the user needs to change the working directory at the beginning of the dofile to the folder where they downloaded the DHS data.

predictors -> Reproduces Figure C.4: Predictive power of own subjective survival probabilities

KernelregressionsPG -> Reproduces Figure C.5: nonparametric estimates of update in beliefs

benknow-figure-C1A+B -> Reproduces Figure C.1

benknow-figure-1A-mort-trends.r -> Reproduces Figure 1a

mylegend.R -> This is just a function called by “benknow-figure-1A-mort-trends.r” and does not need to be run independently.

benknow-figure-1B-mort-expectations.r -> Reproduces Figure 1b

List of tables and figures
The provided code reproduces all numbers provided in text in the paper and all tables and figures. Tables are stored in the “tables” folder while figures are stored in the “figures” folder.

Figure/Table	Program	Output file	Note
Table 1	descriptive	table1_A.tex; table1_B.tex; table1_C.tex	
Table 2	n/a		no data; manually created by authors
Table 3	analysis_main; bootstrap_ci	table3panelA.tex; table3panelB.xlsx; table3panelB_confidence_intervals.xls	confidence intervals in Panel B are created by bootstrap_ci
Table 4	benknow_sane	table4.tex	
Table 5	analysis_main	table5.tex	
Table 6	analysis_main	table6_panelA.tex; table6_panelB.tex	
Table 7	analysis_main	table7.tex	
Figure 1	benknow-figure-1A-mort-trends.r; benknow-figure-1B-mort-expectations.r	figure1a.pdf; figure1b.pdf	This is R code. Need to change working directory at the beginning of the file
Figure 2	n/a		no data; manually created by authors
Figure 3	n/a		no data; manually created by authors
Figure 4	n/a		no data; manually created by authors

Online Appendix

Figure/Table	Program	Output file	Note
Table C.1	analysis_main	tableC1hiv-.csv; tableC1female.csv; tableC1male.csv	
Table C.2	analysis_main	tableC2.tex	
Table C.3	analysis_main	tableC3.tex	
Table C.4	analysis_main	tableC4.tex	
Table C.5	analysis_main	tableC5.csv	
Table C.6	analysis_main	tableC6.tex	
Table C.7	descriptive	tableC7.tex	
Table C.8	analysis_main	tableC8.tex	
Table C.9	analysis_main	tableC9.tex	
Table C.10	descriptive	tableC10panelA.tex; tableC10panelB.tex	
Table C.11	descriptive	tableC11panelA.tex; tableC11panelB.tex	
Table C.12	analysis_main	tableC12panelA.tex; tableC12panelB.tex	
Table C.13	analysis_main	tableC13.tex	
Figure C.1	benknow-figure-C 1A+B	figure-C1a.pdf; figure-C1b.pdf	
Figure C.2	prevalence	figureC2.png	Requires access to DHS data
Figure C.3	descriptive	figureC3a.pdf; figureC3b.pdf	
Figure C.4	predictors	figureC4a.png; figureC4b.png	
Figure C.5	KernelregressionsPG	figureC5	

References:

Hans-Peter Kohler, Susan C Watkins, Jere R Behrman, Philip Anglewicz, Iliana V Kohler, Rebecca L Thornton, James Mkandawire, Hastings Honde, Augustine Hawara, Ben Chilima, Chiwoza Bandawe, Victor Mwapasa, Peter Fleming, Linda Kalilani-Phiri, Cohort Profile: The Malawi Longitudinal Study of Families and Health (MLSFH), *International Journal of Epidemiology*, Volume 44, Issue 2, April 2015, Pages 394–404, https://doi.org/10.1093/ije/dyu049

Global Burden of Disease Collaborative Network. (2017). Global burden of disease study 2016 (GBD 2016) results. Seattle, United States: Institute for Health Metrics and Evaluation (IHME).
