# Supplementary material for: MORTALITY RISK INFORMATION, SURVIVAL EXPECTATIONS AND SEXUAL BEHAVIOURS
Source: Econ J (London). Author manuscript; Available in PMC 2025 May 1. (PMC11065140; doi:10.1093/ej/uead116)
Supplement: Zipped Data - Replication File [file 2017-04-penn-benefits-of-knowledge-approval.pdf › 3-replication-package/2017-04-penn-benefits-of-knowledge-approval.pdf]

University of Pennsylvania
Office of Regulatory Affairs
3624 Market St., Suite 301 S
Philadelphia, PA 19104-6006
Ph: 215-573-2540/ Fax: 215-573-9438

INSTITUTIONAL REVIEW BOARD

(Federalwide Assurance # 00004028)

13-Apr-2017

Hans-Peter Kohler
hpkohler@pop.upenn.edu

PRINCIPAL INVESTIGATOR	: Hans-Peter Kohler
TITLE	: The Benefits of Knowledge: Mortality Risks, Mental Health and Life-Cycle Behaviors
SPONSORING AGENCY	: National Institute on Aging/NIH/DHHS
PROTOCOL #	: 826828
REVIEW BOARD	: IRB #7

Dear Dr. Kohler:

The above referenced protocol and was reviewed and approved using the expedited procedure set forth in 45 CFR 46.110, category 5,7, on 12-Apr-2017. This study will be due for continuing review on or before 11-Apr-2018.

Approval by the IRB does not necessarily constitute authorization to initiate the conduct of a human subject research study. Principal investigators are responsible for assuring final approval from other applicable school, department, center or institute review committee(s) or boards has been obtained. If any of these committees require changes to the IRB-approved protocol and informed consent/assent document(s), the changes must be submitted to and approved by the IRB prior to beginning the research study.

If this protocol involves cancer research with human subjects, bio-specimens, or data, you may not begin the research until you have obtained approval or proof of exemption from the Cancer Center’s Clinical Trials Review and Monitoring Committee.

The following documents were included in this review:

- HS-ERA Initial Submission, confirmation , submitted 04/03/2017
- Cover Letter, dated 04/03/2017
- Informed Consent Form, uploaded 04/03/2017
- Study Materials and Instruments, dated 04/03/2017
- Protocol References, dated 04/03/2017
- Protocol Figures, dated 04/03/2017

When enrolling subjects at a site covered by the University of Pennsylvania's IRB, a copy of the IRB approved informed consent form with the IRB approved from/to stamp must be used unless a waiver of written documentation of consent has been granted.

If you have any questions about the information in this letter, please contact the IRB administrative staff. Contact information is available at our website: http://www.upenn.edu/IRB/directory.

Thank you for your cooperation.

Sincerely,

Amanda O'Hara

Digitally signed by Amanda O'Hara
Reason: I attest to the accuracy and integrity of this document
Date: 2017.04.13 14:29:00 -04'00'

IRB Administrator
